# The mutualistic fungi of the bark beetle *Pityokteines vorontzowi* are nutrient-rich and efficiently deplete their medium of fir chemical defenses

**DOI:** 10.1093/ismeco/ycag131

**Published:** 2026-05-13

**Authors:** Björn Lichnock, Sifat Munim Tanin, Michael Reichelt, Peter Biedermann, Jonathan Gershenzon, Maximilian Lehenberger

**Affiliations:** Max Planck Institute for Chemical Ecology, Department of Biochemistry, Hans-Knöll Straße 8, 07745 Jena, Thuringia, Germany; Chair of Forest Entomology and Protection, Institute of Forestry, University of Freiburg, Fohrenbühl 27, 79252 Stegen-Wittental, Baden-Württemberg, Germany; Max Planck Institute for Chemical Ecology, Department of Biochemistry, Hans-Knöll Straße 8, 07745 Jena, Thuringia, Germany; Chair of Forest Entomology and Protection, Institute of Forestry, University of Freiburg, Fohrenbühl 27, 79252 Stegen-Wittental, Baden-Württemberg, Germany; Max Planck Institute for Chemical Ecology, Department of Biochemistry, Hans-Knöll Straße 8, 07745 Jena, Thuringia, Germany; Faculty of Forestry and Wood Sciences, University of Life Sciences Prague, Kamýcká 129165 00 Prague - Suchdol, Czech Republic; Max Planck Institute for Chemical Ecology, Department of Biochemistry, Hans-Knöll Straße 8, 07745 Jena, Thuringia, Germany

**Keywords:** bark beetle, silver fir, phloem, mutualistic fungi, plant defenses, metabolism

## Abstract

Conifers are a challenging host for herbivores since their tissues are very low in essential nutrients but high in chemical defenses. For herbivorous insects, such as phloem-colonizing bark beetles, mutualistic fungi may improve their diet by providing a nutritious mycelium. A recent study revealed that two filamentous fungi are mutualists of the European fir engraver beetle *Pityokteines vorontzowi*, but a potential nutritional contribution of the fungi, as well as their capability to degrade plant antiherbivore defenses remains unknown. We analyzed the nutrient content of the fungal mutualists *Ophiostoma piceae* and *Geosmithia* sp. F1 and examined their ability to degrade the constitutive chemical defenses of silver fir phloem in comparison to other fungi. Both mutualists turned out to be rich in amino acids, sugars, and B vitamins and were found to efficiently deplete their phloem media of several defenses. Strikingly, *O. piceae* not only accumulated the highest amounts of the B vitamin nicotinic acid of the 17 tested fungi but also showed a high ability to deplete its medium of chemical defenses, similar to the behavior of the *Ips typographus* mutualist *Endoconidiophora polonica*. Beetle-vectored, non-mutualistic fungi isolated from *P. vorontzowi* showed similar capacities to deplete defensive compounds, whereas non-fir-associated fungi were less effective in reducing their concentrations in the phloem medium. The nutritious mycelium of *O. piceae* and *Geosmithia* sp. F1 and the ability of these fungi to deplete the medium of major fir defense compounds likely facilitates the colonization of silver fir phloem by *P. vorontzowi*.

## Introduction

A vast diversity of insects worldwide colonizes the various tissues of woody plants. Bark and ambrosia beetles, encompassing >6000 currently described species, colonize the inner bark (phloem) or sapwood (xylem), respectively, of a broad spectrum of broadleaved and coniferous trees [1, 2]. Although the majority of species colonize weakened or recently dead trees, some bark and ambrosia beetles are well-known pests in forests and plantations that kill large numbers of healthy trees every year [3–6].

Bark and ambrosia beetles manage to successfully reproduce in a challenging substrates like phloem and xylem, which usually exhibit low concentrations of essential nutrients [7, 8]. Woody vascular tissues mostly consist of cellulose, hemicelluloses, and polyphenolics such as lignin, which are scarcely accessible for insect metabolism [9]. Essential elements such as nitrogen, phosphorus, and sulfur, as well as simple sugars, amino acids, and B vitamins, are highly limited [8]. Phloem and xylem tissue is also chemically well defended as it is rich in various chemicals possessing a broad spectrum of antimicrobial and antiherbivore activities [10–12]. In conifers, a large diversity of terpenes and phenolic compounds have been described [13–15]. Some of these defenses are constitutive, while others are induced and are, in some cases, even produced in higher amounts in specialized cells during insect attack [16, 17]. For example, phenolic compounds, which are key components of conifer defense against bark beetles and their associated fungi often increase in concentration upon beetle attack or fungal infection, indicating an induced defensive role [18–23]. Furthermore, plant metabolites such as quinic acid and shikimic acid with known antimicrobial functions are present in high amounts in coniferous phloem as well [24–26], even though their impact on insect herbivores remains unclear. The large chemical arsenal of plant defenses in combination with the poor availability of nutrients in the vascular tissues of tree stems may have selected for strategies to overcome such challenges [27–29].

One way for insects to fulfill their nutritional requirements is by association with microbes such as filamentous fungi [1]. Indeed, bark and ambrosia beetles are generally associated with various fungi, of which certain species are often considered as mutualists, especially in case of ambrosia beetles [1, 30]. These fungal mutualists are believed to contribute to the survival of beetles by providing a range of nutrients such as amino acids, vitamins, sugars, ergosterol, and fatty acids [29, 31–33]. In ambrosia beetles, adults and larvae typically feed on nutrient-rich fruiting structures (e.g. conidiospores) that cover the walls of their galleries [34]. However, for many bark beetle species the preferred fungal tissue for feeding, mycelium or conidiospores, remains unknown. Mutualistic fungi have also occasionally been reported to metabolize phenolic plant defenses [13, 35]. To ensure a successful transmission of their required fungal symbionts, all ambrosia beetles, as well as many bark beetles, possess a highly specialized organ (mycetangium), allowing them to transmit mutualistic associates to new host trees [36, 37]. The evolution of mycetangia highlights the importance of transporting fungi and indicates their essential functions in the colonization of attacking beetles. Besides mutualistic fungi, most wood-dwelling beetles often vector other fungi within their guts or on their exocuticular surface, which are typically less abundant in their breeding systems compared to the mutualistic associates [38–40]. These species may naturally occur on the host tree, for instance, as endophytes or plant pathogens [39, 41–43]. In beetle galleries, they might compete for resources [44], but detailed studies have rarely been conducted.

The European fir engraver beetle *Pityokteines vorontzowi* (Jacobs) is a widespread but poorly studied bark beetle species colonizing silver fir (*Abies alba*), together with other *Pityokteines* species [45]. Although this insect is considered as a secondary bark beetle infesting weakened or recently dead trees, it can cause economic damage in combination with other species [46]. A recent study examining the fungal community of adult field-collected *P. vorontzowi* beetles reported *Ophiostoma piceae* and *Geosmithia* sp. F1 as the most frequently isolated species [47]. In the present study, we confirmed the identities of these fungi via phylogenetic analysis of the large subunit (28S rRNA, LSU) gene. Both fungal species were found to be highly attractive to beetles in both volatile and gustatory behavioral assays and were therefore considered as mutualistic fungi [47]. However, the function of both species in the mutualism remains unknown.

Because both fungi induced the tunneling of the fir engraver beetle in fungus-colonized media [47], we hypothesized they improve the diet of beetles by providing essential nutrients in their mycelium while degrading major antiherbivore constitutive defenses present in silver fir phloem. First, we analyzed the nutritional composition of fungal mycelium (free amino acids, soluble sugars, and B vitamins) grown on fir phloem medium to evaluate the potential contribution of mutualistic fungi to beetle nutrition in a host-specific context. These metabolites contribute to the nutritional requirements of bark beetles and complement the more widely studied limiting nutrients such as nitrogen, phosphorus, and sterols (see literature above). Here, we focused our analyses on mycelium, though it is currently unknown whether adult beetles and larvae of *P. vorontzowi* primarily feed on mycelium, conidiospores, or a combination of fungal tissue and phloem. Second, we quantified the major constitutive defensive compounds in uncolonized and fungus-colonized fir phloem medium and conducted inhibition assays with individual compounds on potato dextrose agar (PDA) to assess the sensitivity of different fungi to these compounds. Third, we performed experiments using individual compounds on PDA medium to determine the ability of tested fungi to deplete the medium of specific chemical defenses. Together, these experiments allowed us to compare several fungi with different life strategies and link their metabolic traits to both nutritional provisioning and chemical defense interactions relevant to the fir engraver beetle-silver fir system. Besides the two mutualists *O. piceae* and *Geosmithia* sp. F1, this study included a broad range of additional fungi of other types, some from the previous fir engraver beetle study [47]. We compared the two identified mutualists to insect (*Beauveria*) and fungal pathogens (*Trichoderma*), hereafter referred to as pathogenic fungi [48–50] isolated from *P. vorontzowi*, to other *P. vorontzowi*–vectored, non-mutualists and to mutualistic fungi associated with other bark and ambrosia beetles. Our results highlight the potential of the mutualists *O. piceae* and *Geosmithia* sp. F1 to serve as valuable food sources for adult fir engraver beetles and to metabolize fir antiherbivore defenses.

## Materials and methods

### Fungal species

We included seven different fungal species in our study that are either mutualistic associates of the fir engraver beetle *P. vorontzowi* or of beech-colonizing ambrosia beetles or the spruce bark beetle *I. typographus* [4, 47, 51–53]. Additionally, we included two fungi that might act as pathogens of insect or fungi, as well as eight further fungi that were, together with the two pathogenic species, recently isolated from adult *P. vorontzowi* beetles in minor abundance [47]. We designated this last category as beetle-vectored, non-mutualistic fungi. By comparing fungi with different life strategies (mutualists, non-mutualists, and pathogenic species) on the same culture medium, we aimed to identify traits that are specific to *P. vorontzowi* mutualists and those that may reflect more general fungal nutritional or defensive interactions. See also Suppl. Table S1 for the individual 17 species and further information, as well as SI Methods for further details.

### Sequencing and maximum likelihood analyses

**Figure 1:**
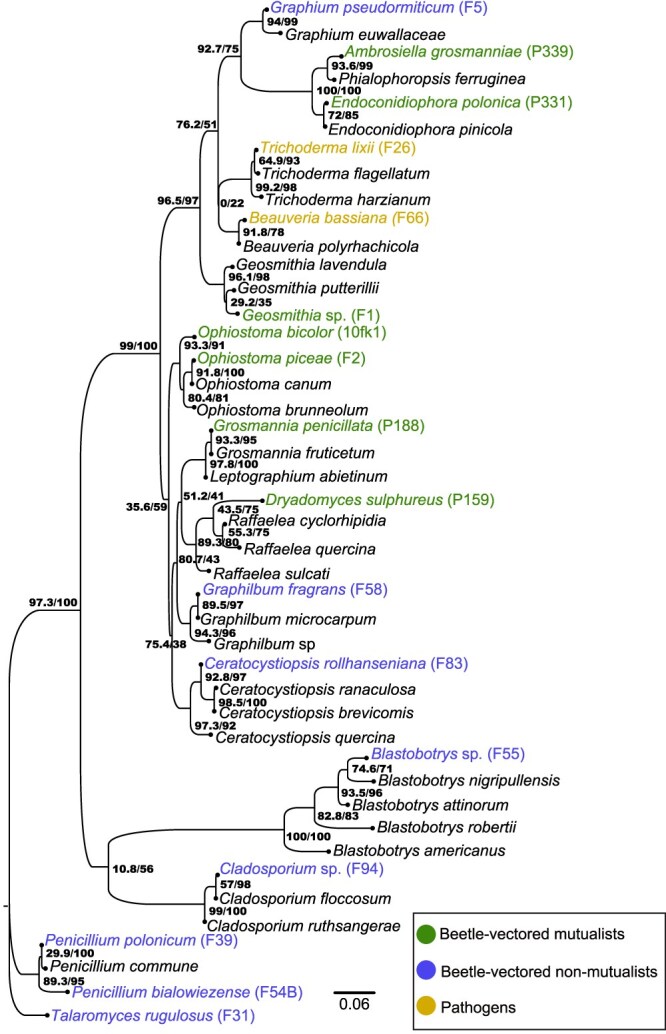
Maximum likelihood phylogeny of all fungi investigated in this study based on fungal 28S or large subunit (*LSU*) ribosomal gene sequences. Highlighted fungi were investigated in this study (*N* = 17), while sequences from remaining fungi (*N* = 27) were obtained from NCBI GenBank. The fungal species *E. polonica*, *O. bicolor*, and *G. penicillata* are vectored by *I. typographus*, while *A. grosmanniae* and *D. sulphureus* are associated with the ambrosia beetles *X. germanus* and *X. saxeseni*. All remaining highlighted fungal species were vectored by and isolated from *P. vorontzowi*. Shimodaira–Hasegawa approximate likelihood ratio test (SH-aLRT) support values (left number) and ultrafast bootstrap support values (right number) were provided in our phylogenetic replacement.

To gain an overview of the taxonomic relationships among the fungi used and to confirm the identity of fungi previously isolated from *P. vorontzowi* [47], we performed a phylogenetic analysis employing the large subunit gene (28S rRNA, *LSU*). First, we sequenced the gene for all species included. DNA was extracted from pure fungal biomass using the Qiagen Blood and Tissue DNA extraction kit, following the manufacturer’s instructions and quality-assessed with a nano-spectrophotometer (Nanodrop 2000c, Thermo Fischer Scientific). Then, we amplified *LSU* with a PCR using the common primers LROR (GTACCCGCTGAACTTAAGC) and LR-5 (ATCCTGAGGGAAACTTC, see ref. [54]) and the premixed MyTag Red Mix (Meridian Bioscience, Germany) as master mix. The PCR program started with a denaturation step of 4 min at 95°C, followed by 30 cycles of 45 s at 94°C, 30 s at 55°C and 60 s at 72°C, and a final extension step at 72°C for 6 min. The resulting products (~900 bp) were purified using the Wizard SV Gel and PCR clean-up system (Promega, Germany) and finally sequenced with a 3730XL DNA Analyzer (Applied Biosystems, Thermo Fisher Scientific). Sequences were individually inspected using the SnapGene Viewer v6.1 and aligned with the respective forward and reverse sequence for each fungal species using R software (version 4.5.1) and the packages “Biostrings” and “stringr” [55, 56]. Final sequences were BLASTed afterwards against the National Center for Biotechnology Information (NCBI) database to assign the taxonomy to species level (>99% identity match). All obtained sequences were uploaded to NCBI Genbank with the accession numbers PX884890–PX884906. For the maximum likelihood analyses (Fig. 1), we either used sequences generated in this study (*N* = 17) or downloaded phylogenetically related reference species (*N* = 27) from NCBI Genbank (Suppl. Table S2). See SI Methods for further details on the phylogenetic analyses.

### Nutritional profiling

To examine the potential nutritional value of the fungi (*N* = 17), we inoculated a plug of pure and actively growing mycelium (Ø 6 mm) from each species on silver fir (*A. alba*; hereafter referred to as fir) phloem medium (5% phloem powder and 2% agar in water). We used fir-based medium as we aimed to compare the two mutualistic fungal associates of the fir engraver beetle *P. vorontzowi* (*O. piceae*, *Geosmithia* sp. F1) with the eight fir beetle-vectored, non-mutualists in our study (*Graphium pseudormiticum*, *Talaromyces rugulosus*, *Penicillium polonicum*, *Blastobotrys* sp. F55, *P. bialowiezense*, *Graphilbum fragrans*, *Ceratocystiopsis rollhanseniana*, *Cladosporium* sp. F94), and additional mutualistic fungi associated with other beetles, including the spruce-colonizing *I. typographus* (*Endoconidiophora polonica*, *Grosmannia penicillata*, *Ophiostoma bicolor*) and the ambrosia beetles *Xyleborinus saxesenii* and *Xylosandrus germanus* (*Dryadomyces sulphureus sulphureus*, *Ambrosiella grosmanniae*). Additionally, we included two pathogenic fungi (*Trichoderma lixii*, *Beauveria bassiana*) isolated from *P. vorontzowi* in a previous study [47]. We chose this set of fungi due to their varying life history strategies (mutualists, non-mutualists, and pathogenic species). We chemically analyzed mycelium grown on fir phloem to determine whether or not they could enhance beetle nutrition in a host-tree context. This would not be reflected in standard laboratory media, which are typically rich in nutrients and so often express only a subset of their natural metabolic potential [57–59]. See SI Methods for further details.

### Free amino acids

We prepared extracts by homogenizing dried fungal biomass with 1 ml of methanol (Honeywell, Germany) and three metal beads (Ø 3 mm, Askubal) on a paint shaker for 5 min. Afterward, we centrifuged samples at 9400 rcf for 2 min and supernatant was collected for further analyses. See SI Methods for further details.

### Soluble sugars

We used the same methanolic extracts mentioned above for the quantification of soluble sugars from fungal biomass. First, extracts were 1:10 diluted in water containing 5 μg/ml ^13^C_6_-glucose (Sigma-Aldrich) and 5 μg/ml ^13^C_6_-fructose (Toronto Research Chemicals, Toronto, Canada), and analyzed by Liquid Chromatography-Tandem Mass Spectrometry (LC-MS/MS) as described [60]. See SI Methods for further details.

### B vitamins

The methanol extracts generated for the analysis of free amino acids were also used for the quantification of B vitamins. Here, 50 μl of the extract was mixed with 50 μl of methanol containing 200 ng/ml D3-thiamine (B1), 200 ng/ml ^13^C,^15^N_2_-riboflavin (B2), 200 ng/ml D4-nicotinic acid (B3), 200 ng/ml D5-pyridoxine (B6), and 200 ng/ml ^13^C_3_,^15^N-pantothenic acid (B5) (all Toronto Research Chemicals, Toronto, Canada) as internal standards. See SI Methods for further details.

### Identification of major silver fir constitutive phloem defenses

For the quantification of major constitutive defenses in fir phloem, as well as to determine fungal depletion of chemicals from the medium, we cultivated all 17 fungi on freshly prepared phloem medium and analyzed methanolic extracts of the fungus-colonized medium and in the fungal biomass by LC-MS. See SI Methods and Suppl. Table S4 for details.

### Inhibition assays

To determine if the previously identified major constitutive defense compounds in fir phloem inhibit the growth of fungi, we conducted bioassays. Besides the phenolic acids, which are well known for their antimicrobial nature [61–66], we included the metabolites, quinic acid and shikimic acid, which are abundant in phloem, as well and reported to be inhibitory toward microbes [24–26, 67, 68]. Applied concentrations were based on the highest levels identified in uncolonized fir phloem in this study (Suppl. Table S5) except for gallic acid, where we used concentrations identified in the presence of *Blastobotrys* sp. F55 as this fungus was isolated from *P. vorontzowi*. We chose a total of six representative fungi (associated with *P. vorontzowi*: *Geosmithia* sp. F1, *O. piceae*; beetle-vectored non-mutualists: *Blastobotrys s*p. F55, *P. bialowiezense*; associated with *I. typographus*: *E. polonica*; associated with *X. saxesenii*: *D. sulphureus*) to allow a direct comparison between fungi with different niches. See SI Methods for further details.

### Depletion of individual constitutive defense compounds

As the majority of fungi tested depleted the fir phloem medium of defense compounds, we tested fungi with individual compounds to explore this trend. Here, we focused our analyses on the two mutualists of the fir bark beetle (*Geosmithia* sp. F1, *O. piceae*), as well as on two beetle-vectored non-mutualists (*P. bialowiezense*, *Blastobotrys* sp. F55). Fungi were cultivated on PDA individually supplemented with six different compounds as described for the inhibition assays. See SI Methods for further details.

## Results

### The fungi classified as fir engraver beetle mutualists, *O. piceae* and *Geosmithia* sp. F1, are rich in nutrients including soluble sugars, free amino acids, and B vitamins

**Figure 2:**
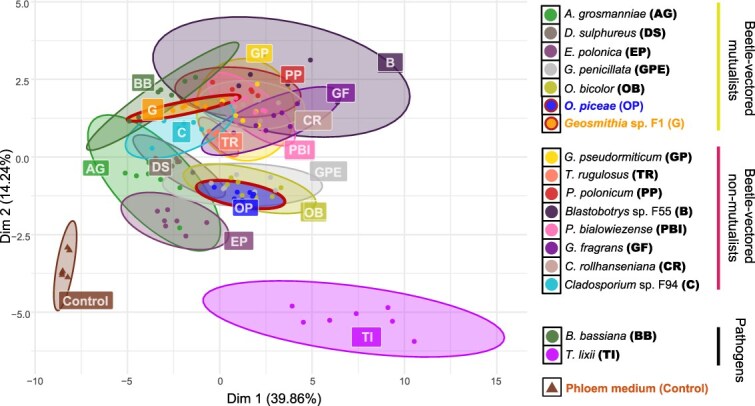
Differences in the nutrient profiles of *P. vorontzowi*–associated mutualists and other fungi when grown on fir phloem medium. The *P. vorontzowi*–associated mutualists differ from one another and from the uninfected fir phloem. The content of free amino acids, soluble sugars, and B vitamins in actively growing fungal biomass (*N* = 5–7) was analyzed from cultures inoculated on 5% fir phloem medium. Fungal biomass was generally harvested as soon as the petri dishes were completely covered or after a max. of 14 d. The nutrient content of all fungi (*N* = 17) was compared using a Principial Component Analysis (PCA) followed by pairwise PERMANOVAs (see Suppl. Table S3-3a for detailed results of statistical analyses). The mutualistic associates of the fir engraver beetle *P. vorontzowi* (*O. piceae*, *Geosmithia* sp. F1) are highlighted in the legend. Abbreviations for each fungus are given in the respective ellipses in the PCA plot and listed in the legend. See also Suppl. Figs S1, S2, and  S4 for heatmaps visualizing all individual amino acids, sugars, and B vitamins identified in fungal biomass.

**Figure 3:**
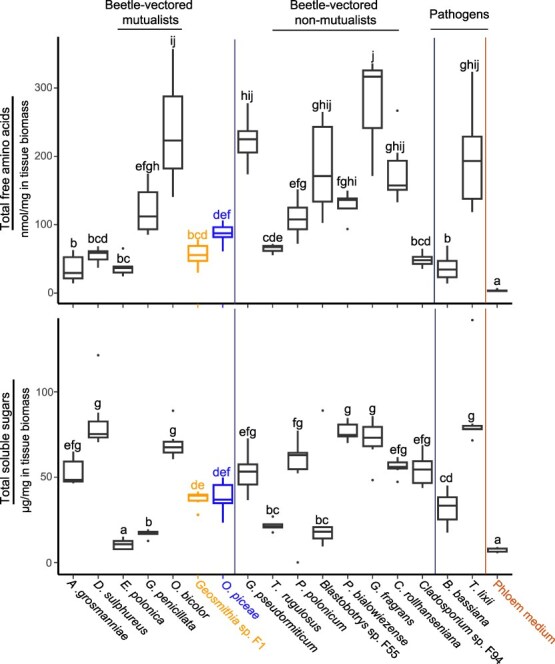
Free amino acids and soluble sugars vary across fungal species grown on fir phloem medium, with moderate levels observed in both the *P. vorontzowi*–associated mutualists. Upper panel: Boxplots comparing the total content of free amino acids (based on the sum of 19 analyzed amino acids, *N* = 5–7 per fungus) in nmol/mg. Lower panel: Boxplots comparing the total content of soluble sugars (based on the sum of four analyzed sugars and one sugar alcohol, *N* = 5–7 per fungus) in μg/mg. Letters above each boxplot indicate significant differences between all 17 fungi and fungus-free fir phloem (control), see Suppl. Table S3 for individual *P*-values (fitted GLMs with adjusted pairwise contrasts), as well as Suppl. Figure S3 for B vitamins.

The nutritional value of 17 mutualistic and nonmutualistic fungi was compared while growing on a minimal fir phloem-based medium (Figs 2 and 3 and Suppl. Figs S1–S4). A PCA analysis indicated a significant difference among the taxa analyzed (Permutational multivariate analysis of variance [PERMANOVA], *F* = 77.2, *R^2^* = 0.93, *P* = .001; Fig. 2), while pairwise PERMANOVAs indicated that all fungi significantly differed in their nutritional profile from the uncolonized fir phloem-based medium used as a control (*P* < .0031; see Suppl. Table S3a for individual *P*-values). Free amino acids and B vitamins were always more abundant in fungal tissue compared to the control (*P* < .001, fitted General Linear Model [GLM] with adjusted pairwise contrasts, Fig. 3 and Suppl. Fig. S4, see Suppl. Table S3 for individual *P*-values). For soluble sugars, all but *E. polonica* accumulated significantly higher quantities than the control (*P* < .001, Fig. 3).

The individual nutrient profiles of examined fungi showed a large diversity and differed strongly from each other (*P* < .0031; pairwise PERMANOVAs, see Suppl. Table S3a for individual *P*-values). One *P. vorontzowi* mutualist, *O. piceae*, grouped together with the major *I. typographus* mutualistic fungi such as *E. polonica*, *G. penicillata*, and *O. bicolor*, while the second *P. vorontzowi* mutualist *Geosmithia* sp. F1 differed from *O. piceae* (*P* < .0021; pairwise PERMANOVAs) and clustered with some of the fir beetle-vectored, non-mutualists such as *P. bialowiezense* and *Cladosporium* sp. F94 and the potential pathogen *B. bassiana* (Fig. 2). This separation between *O. piceae* and *Geosmithia* sp. F1 is mostly explained due to differences in the contents of alanine and gamma-aminobutyric acid (Suppl. Fig. S1), glucose and trehalose (Suppl. Fig. S2), and nicotinic acid and pyridoxine (Suppl. Fig. S4), which were all more abundant in *O. piceae* compared to *Geosmithia* sp. F1. On the other hand, *Geosmithia* sp. F1 accumulated riboflavin and the sugar alcohol mannitol at levels comparable to those in several of the other beetle-associated, fir-colonizing fungi (Suppl. Figs S2 and S4). The total content of soluble sugars in *O. piceae* and *Geosmithia* sp. F1 was significantly higher compared to other bark beetle mutualists such as *E. polonica* and *G. penicillata* (*P* < .001) due especially to glucose, trehalose, and mannitol (see also Suppl. Fig. S2). These results show that both the *P. vorontzowi* mutualists have a similar potential to serve as sources of insect nutrition, being rich in free amino acids, soluble sugars, and B vitamins.

### Most fungi associated with the fir engraver beetle decrease levels of constitutive chemical defenses in fir medium, independently of being mutualist

**Figure 4:**
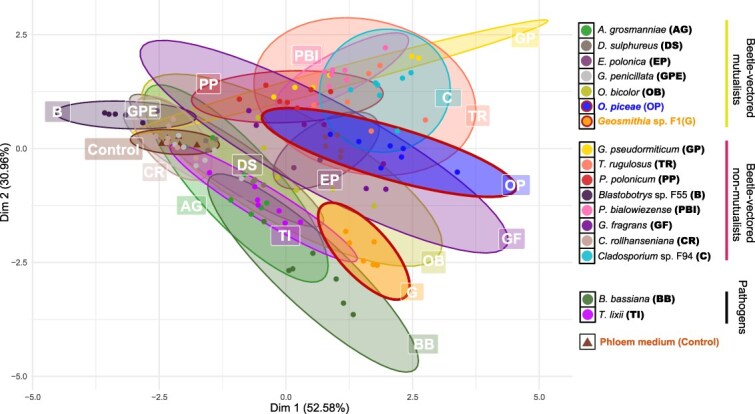
Fungal species differ in their ability to deplete the constitutive chemical defenses in fir phloem medium, with the *P. vorontzowi*–associated mutualists being different from one another. The content of five abundant chemical defenses was analyzed in 5% fir phloem medium colonized by 17 different fungi (*N* = 5–7) and compared to a control without fungal growth. Fungal biomass was harvested as soon as the petri dishes were completely covered or after a max. of 14 d and were analyzed separately (see Suppl. Fig. S5). Biomass and medium were separated by a layer of added cellophane. The amounts of chemical compounds were compared between all examined fungi (*N* = 17) using a Principal Component Analysis (PCA) followed by pairwise PERMANOVAs (see Suppl. Table S3-3b for detailed results of statistical analyses). The mutualistic associates of the fir engraver beetle *P. vorontzowi* (*O. piceae*, *Geosmithia* sp. F1) are highlighted in the legend. Abbreviations for each fungus are given in the respective ellipses in the PCA plot and further listed in the legend.

**Figure 5:**
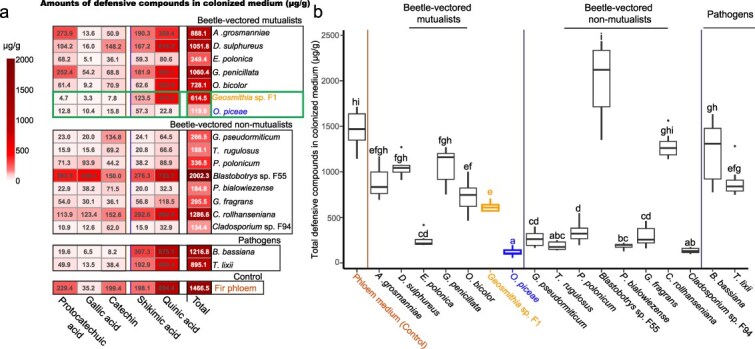
Mutualistic and nonmutualistic beetle-vectored fungi efficiently deplete the medium of fir constitutive chemical defenses. (a) Heatmaps showing the average quantities of five individual compounds (in μg/g dried substrate) detected by targeted LC-MS analyses in fungus-free fir phloem medium (Control), as well as in fir phloem medium, after fungal inoculation with 17 different fungal species (*N* = 5–7 per fungus). Fungal biomass was generally harvested as soon as the petri dishes were completely covered or after max. 14 d. Samples were only taken from areas on the petri dish that were colonized by the fungus. Medium and biomass were analyzed separately (see Suppl. Figure S5 for compounds in fungal biomass). The rightmost column contains the total amounts of defense chemicals in the medium (based on the sum of the five identified substances indicated with white letters) for each individual fungus and control was added. (b) Boxplots showing the total amounts of the five major compounds in fir phloem (Control) and their amount in fungus-colonized medium. Letters above each boxplot indicate significant differences between fungi and control (see Suppl. Table S3 For *P*-values; fitted GLMs with adjusted pairwise contrasts).

As determined by LC-MS/MS, protocatechuic acid, catechin, gallic acid, and vanillic acid were the major phenolic compounds in fir phloem medium, while shikimic acid and quinic acid were also abundant. We then inoculated 17 fungi on this medium and looked for changes in the levels of defense compounds in both the colonized medium and fungal biomass (Figs 4 and 5 and Suppl. Fig. S5).

There was a general difference between the examined species (PERMANOVA, *F* = 31, *R^2^* = 0.83, *P* = .001; Fig. 4), with fir engraver beetle-vectored, but non-mutualistic fungi being especially efficient in reducing the concentrations of fir phloem defense chemicals (Figs 4 and 5). Indeed, all nonmutualistic fungi, except *Blastobotrys* sp. F55 and *C. rollhanseniana*, were among the strongest degraders of the defense compounds tested and significantly differed from nine of the remaining fungi (*P* < .0037, fitted GLM with adjusted pairwise contrasts, Fig. 5). All six fungi significantly decreased the total contents of the fir phloem defenses (Fig. 5, *P* < .0491). Interestingly, one species of the beetle-vectored, non-mutualistic group, *Blastobotrys* sp. F55, accumulated several of the measured compounds, especially gallic acid, in the medium. Gallic acid was present in the *Blastobotrys* sp. F55 medium at a concentration over 10-fold that in the fir phloem control medium (Fig. 5). The two *P. vorontzowi* mutualists, *O. piceae* and *Geosmithia* sp. F1, differed significantly from one another in their potential to degrade the analyzed fir chemical defenses (*P* < .0025; pairwise PERMANOVA), clustering with different groups of fungal species (Fig. 4). Among the mutualistic fungi, *O. piceae*, together with *E. polonica*, were most efficient in depleting the medium from such compounds (*P* < .001, fitted GLM with adjusted pairwise contrasts, Fig. 5). The results show that both mutualistic fungi of the fir engraver beetle *P. vorontzowi* reduced the overall content of chemical defenses in fir phloem. However, the majority of the fir engraver beetle-vectored, but nonmutualistic fungi successfully depleted the medium of defense compounds as well.

### The fir engraver beetle mutualists, *O. piceae* and *Geosmithia* sp. F1, tolerate and efficiently deplete the medium of most constitutive chemical defenses in fir phloem

**Figure 6:**
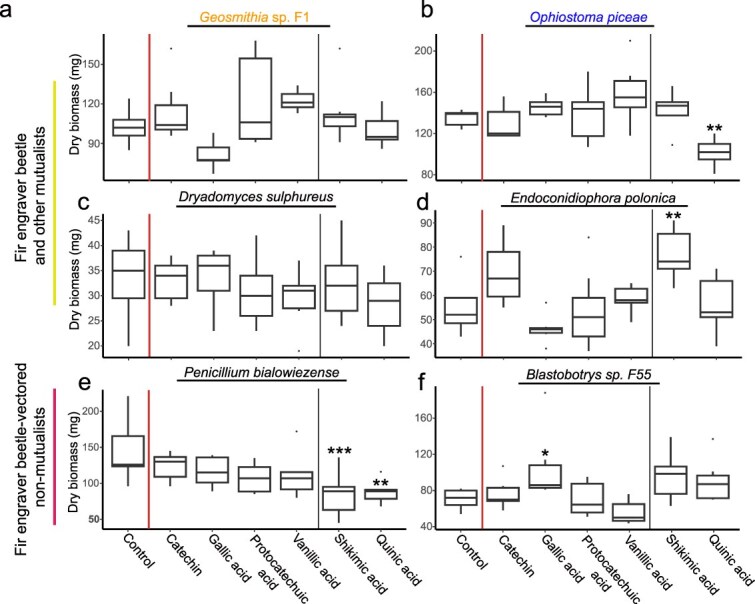
Most fir constitutive chemical defenses showed no inhibitory effect toward mutualistic and nonmutualistic beetle-vectored fungi. The growth of six filamentous fungi on PDA medium individually supplemented with the previously identified six fir chemical defenses (see Suppl. Table S5 for applied concentrations) was analyzed (*N* = 6–7 per fungus and compound). Here, we included the fir engraver beetle mutualists *Geosmithia* sp. F1 (a) and *O. piceae* (b), as well as the other mutualists, *D. sulphureus* (c) and *E. polonica* (d). Additionally, the fungi *P. bialowiezense* (e) and *Blastobotrys* sp. F55 (f) were included to allow a comparison with beetle-vectored, but nonmutualists colonizing a similar substrate (e.g. fir phloem). Asterisks above each boxplot indicate significant differences in fungal growth (mg dried biomass) on medium supplemented with compounds in comparison to a control (PDA) without any additionally added chemicals (see Suppl. Table S3 For *P*-values; fitted GLMs with adjusted pairwise contrasts).

The growth inhibition assays revealed that the majority of the fungi tolerated all six of the fir phloem defense compounds tested (Fig. 6). Of the mutualists, only *O. piceae* was significantly inhibited by one of the compounds, quinic acid (*P* < .0015; fitted GLM with adjusted pairwise contrasts). However, this species completely depleted quinic acid from its culture medium (*P* < .001) (Suppl. Fig. S6). On the other hand, one of the compounds, shikimic acid, actually enhanced the growth of the *I. typographus* mutualist *E. polonica* (*P* < .0066; Fig. 6).

After addition to the culture medium, both *P. vorontzowi* mutualists (*O. piceae* and *Geosmithia* sp. F1) efficiently depleted catechin, protocatechuic acid, gallic acid, and vanillic acid (*P* < .0042; Suppl. Fig. S6). Shikimic acid, however, was not depleted by either fir engraver beetle mutualists, and quinic acid was not degraded by *Geosmithia* sp. F1. Indeed, of the four fungi tested, only the fungus *P. bialowiezense* depleted shikimic acid, as well as the remaining five test compounds from the medium (*P* < .001; Suppl. Fig. S6). However, two compounds significantly inhibited its growth (*P* < .0031; linear model, Fig. 6). The other beetle-vectored, non-mutualistic fungus tested, *Blastobotrys* sp. F55, did not deplete any of the tested compounds from the PDA medium but accumulated all substances except catechin and quinic acid in higher concentrations compared to the control (*P* < .04), similar to what we observed in the previous experiment on fir phloem medium (Fig. 5). Moreover, *Blastobotrys* sp. F55 showed enhanced growth when inoculated on gallic acid (*P* < .044; Fig. 6), a phenolic acid previously shown to be accumulated in the medium by this fungus in the earlier experiment on fir phloem medium (Fig. 5). These results demonstrate that the two mutualistic fir engraver beetle associates tolerate high concentrations of fir chemical defenses and can deplete them from the medium, likely by metabolic degradation.

## Discussion

Phloem tissue of conifers is typically low in nutrients and chemically well defended, being rich in various phenolic compounds that act to deter herbivores, as well as protect against plant-pathogenic microbes [16, 69–71]. The ability of insects like phloem-colonizing bark beetles to survive in such challenging substrates may be promoted by specific microbes that supplement the nutrient content of woody tissue [1, 4]. Additionally, such associated microbes could degrade the chemical defense compounds of the tree, facilitating the colonization process by their insect vectors [13, 72, 73]. Recently, the little-studied fir bark beetle, *P. vorontzowi,* has been found to be associated with two mutualistic filamentous fungi, which it introduces into the phloem of silver fir, *A. alba* [47]. We studied these two mutualists, *O. piceae* and *Geosmithia* sp. F1, focusing on their nutritional potential and their capability to metabolize several constitutive phenolic defensive compounds in fir phloem in comparison with additional fungi. We showed that *O. piceae* and *Geosmithia* sp. F1 were both rich in overall nutrient content but differ in their individual nutrient profiles driven by differences in individual soluble sugars and B vitamins. The growth of both mutualists was usually not inhibited by constitutive chemical defense compounds occurring in fir phloem, and these fungi turned out to efficiently deplete such compounds from their growth medium. Here, *O. piceae* was especially proficient in such depletion, similar to other bark beetle mutualists such as *E. polonica*. The majority of fungi classified as fir engraver beetle–vectored, but non-mutualists showed similar depletion ability.

Wood-dwelling insects such as bark and ambrosia beetles often rely on specific microbes to meet their nutritional requirements when feeding on the nutrient-poor phloem and xylem tissue of trees [32, 71]. Ideal candidates for such nutrient-accumulating microbes are filamentous fungi, which are often capable of depolymerizing major plant polysaccharides allowing them to penetrate into woody tissue and acquire nutrients for their own growth [31, 74]. Both mutualists of the fir engraver beetle *P. vorontzowi* were found to be rich in their overall content of free amino acids, soluble sugars, and B vitamins; however, their individual profiles of nutrients differed. For example, *O. piceae* was especially rich in glucose and trehalose and accumulated the highest amounts for all the 17 tested fungi of the B vitamin nicotinic acid, which was almost absent in *Geosmithia* sp. F1. In turn, this *Geosmithia* sp. taxon accumulated the highest amounts of riboflavin of the mutualistic fungi. B vitamins such as nicotinic acid and riboflavin cannot be synthesized by insects and so must be acquired from the diet to ensure successful development [75, 76]. These compounds are available in only low amounts in fir phloem and the accumulation of B vitamins, as well as sugars and amino acids, in *O. piceae* and *Geosmithia* sp. F1 significantly improves the nutritional value of colonized phloem for growth and development of their beetle associate. The co-occurrence of two or more mutualistic fungi is also known from other bark and ambrosia beetles [34, 38, 77]. These mutualists may provide different but complementary functions for their beetle associate, but this has not been studied. The fir engraver beetle mutualist *O. piceae*, with its high levels of glucose and trehalose, might serve as a particularly good source of sugars for developing larvae with high nutritional requirements [78, 79]. Notably, all the beetle-vectored fungi examined, both mutualists and non-mutualists, showed a higher nutrient content than uncolonized fir phloem medium, a pattern observed previously [33], suggesting that the selection of a mutualist is not based solely on nutritional considerations. Fungi chosen as mutualists by bark and ambrosia beetles likely provide additional benefits for the insect associate. It should be noted that our analyses focused solely on mycelium. Adult beetles and larvae of *P. vorontzowi* may also consume conidiospores, which can differ in their nutrient composition [80], but see also ref. [81]. Comparing the nutrient profiles of mycelium and conidiospores across multiple fungal taxa could provide further insight into the nutritional ecology of bark and ambrosia beetles. Another challenge for insects colonizing woody tissue is to avoid negative consequences from consuming the enormous variety of constitutive plant defensive compounds present [11, 82, 83]. In conifers, various phenolic compounds are well-known defenses against herbivores, with effects on microbial pathogens as well [11, 84, 85]. Microbial associates of insects could contribute to the detoxification and degradation of phenolics as suggested for *E. polonica*, a filamentous symbiont of the Eurasian spruce bark beetle *I. typographus* [13, 35], which may facilitate the colonization success of the insect associate. Indeed, the two mutualists of the fir engraver beetle *P. vorontzowi* (*O. piceae* and *Geosmithia* sp. F1) efficiently depleted the overall amounts of phenolics and other defensive metabolites in fir phloem medium (Fig. 5). This depletion is likely due to uptake and metabolic breakdown because these substances were generally not seen to accumulate in fungal biomass (Suppl. Fig. S5). The accumulation of gallic acid in medium colonized by *Blastobotrys* sp. F55 suggests that this phenolic acid may originate from the hydrolysis of tannins present in the phloem or from the transformation of other phenolic precursors. Alternately, it may also be synthetized *de novo* by *Blastobotrys* sp. F55. However, the growth of both mutualist fungi was not significantly inhibited by any of the compounds, except *O. piceae* by quinic acid, and quinic acid was nevertheless depleted by this fungus. *O. piceae* turned out to be among the most effective species of all the mutualist fungi tested in depleting these compounds from fir phloem medium. In combination with its much faster growth on fir phloem medium compared to *Geosmithia* sp. F1, this underlines the potential of *O. piceae* to function in metabolism of fir chemical defense during the initial stages of *P. vorontzowi* colonization. All constitutive phloem defenses were depleted from the medium by *O. piceae*, while *Geosmithia* sp. F1 depleted phenolic acids but not the other tested plant metabolites.

The fir engraver beetle–vectored nonmutualists in this study also turned out to be highly efficient in depleting the medium of the majority of fir constitutive defenses and so likely metabolized them. Indeed, six out of the eight species in this category depleted not only phenolic acids but also the antimicrobial metabolites quinic acid and shikimic acid when growing on fir phloem medium. In contrast, the fungi classified as insect and fungal pathogens, as well as the majority of nonfir-associated mutualists, showed mostly limited ability to deplete these compounds. Fir-associated fungi might be expected to deplete fir chemical compounds than fungi associated more readily with insects using other host trees or using insects directly as their growth substrates. Indeed, cultivating all fungi on the same silver fir phloem medium allowed standardized comparisons, but for fungi not naturally associated with fir, this may have exposed them to atypical compounds, potentially affecting metabolite degradation compared to their natural environment. However, some of the differences among fungi might also be explained in terms of their life history with associated beetles. For instance, the *I. typographus* mutualist *E. polonica* is considered to be among the first filamentous colonizers of phloem after the initial attack by adult beetles [86, 87], and may thus encounter the highest concentrations of tree defenses. Indeed, *E. polonica* depleted all phenolic and other defense chemicals from fir phloem medium, while other *I. typographus–*associated fungi such as *G. penicillata* and *O. bicolor*, which become more abundant after the initial colonization by *E. polonica* [88], showed lower levels of depletion. However, some of the fungi classified as beetle-vectored nonmutualists belong to widespread plant pathogenic genera or may even be endophytes [42, 43, 89–92]. Thus, they likely colonize host trees in solitary fashion without beetles and so may benefit from being able to detoxify and degrade constitutive defense compounds. Metabolism of such compounds may incur costs. For example, the fungus *P. bialowiezense* was among the most effective fungi in this study at depleting constitutive defense compounds from the medium, which we infer to indicate rapid metabolism. However, its growth was significantly inhibited by shikimic and quinic acids, suggesting that metabolism was costly or did not fully detoxify these defenses [93, 94].

Taken together, we demonstrated that *O. piceae* and *Geosmithia* sp. F1, the fungal mutualists of the fir-colonizing bark beetle *P. vorontzowi*, have the potential to serve as valuable food sources for beetles and their offspring. Their high content of free amino acids, soluble sugars, and B vitamins clearly improves the nutritional quality of the native phloem substrate. Moreover, both fungal mutualists deplete the medium of constitutive plant defenses, likely due to metabolism, with little negative effects on their growth. *Ophiostoma piceae* especially was shown to be among the most efficient fungi at such depletion in this study, which would make it a good mutualist for the early colonization of fir phloem during beetle attack, similar to *E. polonica* in the *I. typographus* system [86, 87]. While our study focused on phenolic defenses, host tree terpenoids may also play an important role in the *P. vorontzowi* system. Silver fir and other fir species produce terpenoids as constitutive and induced defenses [95], and could employ these in defense against bark beetle attack as spruce does [96, 97]. Thus, terpenoids could also influence the interaction of *P. vorontzowi* and its mutualists.

## Supplementary Material

Supplementary_material_ycag131

## Data Availability

All data generated or analyzed during this study are included in this published article (and its supplementary information files).

## Materials availability

This study did not generate new unique reagents.
