# Supplementary material for: The mutualistic fungi of the bark beetle Pityokteines vorontzowi are nutrient-rich and efficiently deplete their medium of fir chemical defenses
Source: ISME Commun. 2026 May 13;6(1):ycag131. doi: 10.1093/ismeco/ycag131 (PMC13245730; doi:10.1093/ismeco/ycag131)
Supplement: Supplementary_material_ycag131 [file supplementary_material_ycag131.zip › Suppl information.pdf]

Supplementary Tables

Suppl. Tables S1: Overview of the 17 fungal isolates used in this study and additional information on their source of isolation.

Suppl. Tables S2: Overview of accession numbers for the LSU sequences used for the maximum likelihood analyses.

Suppl. Tables S3, S3a, S3b: Summary of all statistics and *P* values sorted by figure.

Suppl. Tables S4: a) Analytical details for quantification of B vitamins by LC-MS/MS [HPLC 1260 (Agilent Technologies)-QTRAP6500 (SCIEX)] in positive ionization mode. **b**) Analytical details for the quantification of soluble sugars by LC-MS/MS [HPLC 1200 (Agilent Technologies)-API3200 (Applied Biosystems)] in negative ionization mode. **c**) Analytical details for the quantification of free amino acids by LC-MS/MS [HPLC 1260 (Agilent Technologies)-QTRAP6500 (Sciex)] in positive ionization mode. **d**) Analytical details for the quantification of fir chemical defenses by targeted LC-MS/MS [HPLC 1200 (Agilent Technologies)-API3200 (Applied Biosystems)] in negative ionization mode. Here, the settings for multiple reaction monitoring of each compound, the LC retention time and the supplier of the commercial standards are listed.

Suppl. Tables S5: Overview of tested concentrations for chemical plant defenses applied in the performed inhibition assay including supplier.

Supplementary Figures

Suppl. Fig. S1: Heatmap showing the average quantity of individual free amino acids (N = 5-7) for the 17 investigated fungi in comparison to the fir phloem (Control) in nmol/mg dried biomass. Fungi were inoculated on 5% fir phloem medium until petri dish were completely covered or after a max. of 14 d.

Suppl. Fig. S2: Heatmap showing the average quantity of individual soluble sugars (N = 5-7) for the 17 investigated fungi in comparison to the fir phloem (Control) in µg/mg dried biomass. Fungi were inoculated on 5% fir phloem medium until the petri dish was completely covered or after a max. of 14 d.

Suppl. Fig. S3: The total content of B vitamins was calculated by summing all individual vitamins for each fungus (N = 17). A boxplot depicts the total content of B vitamins (based on the sum of five analyzed B vitamins, N = 5-7 per fungus) in µg/g dried biomass. Letters above each boxplot indicate significant differences between all 17 fungi and fungus-free fir phloem (control). See Suppl. Table S3 for individual *P* values (fitted GLMs with adjusted pairwise contrasts).

Suppl. Fig. S4: Heatmap showing the average quantity of individual B vitamins (N = 5-7) for the 17 investigated fungi in comparison to the fir phloem (Control) in µg/g dried biomass. Fungi were inoculated on 5% fir phloem medium until the petri dish was completely covered or after a max. of 14 d.

Suppl. Fig. S5: Heatmap showing the average quantity of six individual tree defensive chemicals in dried biomass of 17 investigated fungi in comparison to the fir phloem (Control) in µg/g (N = 5-7 per fungus). Fungi were inoculated on 5% fir phloem medium until the petri dish was completely covered or after a max. of 14 d.

Suppl. Fig. S6: Boxplots showing the abundance of added vanillic acid (**a**), catechin (**b**), protocatechuic acid (**c**), gallic acid (**d**), shikimic acid (**e**), and quinic acid (**f**) in µg/g medium. Fungi were inoculated on PDA medium and the compounds added at various concentrations (Suppl. Table 5). Culture medium was harvested and subsequently freeze dried as soon as fungi covered the entire petri dish or after max. 10 d. Letters above each boxplot indicate significant differences between fungi and a fungal-free

control (see Suppl. Table S3 for *P* values; fitted GLMs with adjusted pairwise contrasts).
