# Supplementary material for: The mutualistic fungi of the bark beetle Pityokteines vorontzowi are nutrient-rich and efficiently deplete their medium of fir chemical defenses
Source: ISME Commun. 2026 May 13;6(1):ycag131. doi: 10.1093/ismeco/ycag131 (PMC13245730; doi:10.1093/ismeco/ycag131)
Supplement: Supplementary_material_ycag131 [file supplementary_material_ycag131.zip › Suppl Methods.pdf]

Supplementary Methods

Methods

Fungal species and culture medium

All fungal examined in this study were maintained at -80°C in glycerol (80%) and glycerol / peptone (80% / 1%, Roth; Germany) at the Department of Forest Entomology and Protection (University of Freiburg, Germany) and at the Max-Planck Institute for Chemical Ecology (MPI-CE, Jena, Germany). We revived all fungi before each experiment by cultivating them on 4% potato dextrose agar (PDA; Sigma Aldrich, Germany) and maintained isolates at 4°C until they were sub-cultured on fresh PDA at room temperature for 7 d before they were used for individual experiments. All nutritional analyses as well as the detection of phenolic and related compounds were performed on silver fir phloem medium (5% freshly prepared phloem powder [phloem was removed from freshly cut tree, dried, and milled to a fine powder], 2% agar [Roth, Germany] dissolved in water). Inhibition assays as well as the depletion experiment with defense compounds were done on PDA supplemented with the individual compounds.

Maximum likelihood analyses

To identify the optimal alignment for phylogenetic analyses, we compared different multiple sequence alignment methods (MSA) using the software R (version 4.5.1). We tested four algorithms using the package “msa” (1): Clustal Omega, ClustalW, Muscle, and MAFFT, which were chosen for their robust performance in DNA sequence alignments (2-4). Gblocks Version 0.91b (5) was applied to each alignment using default parameters to filter out positions with high gaps or low conservation with the aim of improving alignment quality by removing poorly aligned or ambiguously aligned regions. Then, we assessed the quality of each alignment and its corresponding

Gblocks-filtered version using a set of four quality metrics: mean Shannon entropy per position, gap percentage, proportion of fully informative positions, and log-likelihood values obtained from maximum likelihood phylogenetic tree inference using neighbor-joining starting trees as implemented in the packages “Biostrings”, “entropy”, “seqinr”, and “phangorn” (6-13). We selected the best Gblocks-filtered version (in this case MAFFT) and used the model finder implemented in IQ-Tree Version 2.2.2 (14) to identify the best-fitting model for our sequence data (TN+F+I+G4), which was chosen for further analyses. Phylogenetic trees were inferred using maximum likelihood with robust branch support estimation, including 6,000 ultrafast bootstrap replicates and Shimodaira-Hasegawa approximate likelihood ratio test (SH-aLRT) branch tests (15). Resulting trees were rooted based on a fungal outgroup using the “ape” package (16). We applied the packages “dplyr”, “tidyverse”, and “stringr” (17-19) for data processing and filtering as well as the package “ggtree” (20) and the software FigTree (http://tree.bio.ed.ac.uk/software/figtree) to visualize the final maximum likelihood tree. Finally, the software Adobe Illustrator (version CS5) was applied for final modifications.

Nutritional profiling

After inoculation, all petri dishes (90 × 14 mm, Roth, Germany) were incubated on cellophane at 25°C and 65% humidity (N = 6 per fungus), either until they were completely covered or for a maximum of 14 d for slow-growing fungi. Collected biomass was freeze-dried and subsequently processed for further analyses. In general, data were normalized based on dry weight, which was measured for each sample. Differences in between individual fungi in free amino acids, soluble sugars, and B vitamins were determined. Statistical analyses were done using the software R (version 4.2.1). A Principal Component Analysis (PCA) was performed with individual free amino acids, soluble sugars, and B vitamins using log1p-transformed and z-scaled

data. To test for significant differences in our model, we applied global as well as pairwise Permutational Multivariate Analyses of Variances (PERMANOVA) using log1p-transformed, but non-scaled data. Bray-Curtis distances were used to quantify dissimilarities, and *P* values from pairwise comparisons were adjusted for multiple testing using the Benjamini-Hochberg method. Additionally, we tested for multivariate homogeneity of group dispersions (beta-dispersion), also with 999 permutations, to confirm that group centroids were not driven by differences in group dispersions. Here, we used the R packages “ggplot2” (21), “factoextra” (22), “ggforce” (23), “FactoMineR” (24), “RColorBrewer” (25), “pairwiseAdonis” (26), and “vegan” (27). For pairwise PERMANOVA comparisons, *P* values were adjusted for multiple testing using the Benjamini-Hochberg method. We visualized the total content of free amino acids, soluble sugars, and B vitamins for individual fungi using boxplots and applied a series of fitted generalized linear models (see ref. (28) for further details on model evaluation and used R packages) to reveal significant differences between individual fungi (see also Suppl. Table S3). Further, we visualized individual free amino acids, soluble sugars, and B vitamins with heatmaps using the packages “ggplot2” (21), “pheatmap” (29), “dplyr” (17), “tidyr” (30), “string” (19), and “reshape2” (31). Finally, all plots were modified using the software Adobe Illustrator (version CS5).

Free amino acids

Free amino acids were quantified from fungal tissue with a targeted LC-MS/MS protocol using a C18-column (XDB-C18, 50 x 4.6 mm, 1.8 µm; Agilent Technologies, Santa Clara, CA, USA) after diluting the methanol extracts 1:10 (v:v) with water containing 10 µg/ml of a mixture of U-^{15}N/^{13}C labeled amino acids (algal amino acid mix, Isotec, Miamisburg, OH, USA) and 5 uM of D5-tryptophan (Cambridge Isotope Laboratories, Inc.; Andover, MA). Further details on the chromatography and mass

spectrometry (Agilent 1260 LC system (Agilent Technologies, Santa Clara, CA, USA) coupled with a QTRAP 6500 tandem mass spectrometer (SCIEX, Darmstadt, Germany) are provided in ref. (32). The positive ionization mode was applied as the operation conditions for the mass spectrometer with multiple reaction monitoring (see Suppl. Table S4 for MRM table). Water containing 0.05% formic acid and acetonitrile was employed as mobile phases A and B, respectively. Overall, all amino acids were quantified relative to the peak area of the corresponding labeled compound, except for asparagine (using U-^{15}N/^{13}C-aspartate and a response factor of 1.0).

Soluble sugars

We used an Agilent 1200 HPLC system coupled to an API 3200 tandem mass spectrometer (AB Sciex, Darmstadt, Germany) for targeted analysis. The HPLC was equipped with a hydrophilic interaction liquid chromatography (HILIC) column (apHera-NH2 Polymer; Supelco, Bellefonte, PA, USA), while chromatographic separation was conducted using water and acetonitrile as mobile phases A and B, respectively, with a flow rate of 1.0 ml/min. We maintained the column temperature at 20°C. The mass spectrometer was equipped with a turbo spray ion source, which we operated in the negative ionization mode. The ion spray voltage was maintained at −4,200 eV, while the turbo gas temperature was set at 500°C. Nebulizing gas was set at 60 psi, curtain gas at 30 psi, heating gas at 60 psi, and collision gas at 4 psi. We used multiple reaction monitoring to analyze analyte precursor ion to product ion transitions (see also Suppl. Table S4 for MRM table). Finally, data were acquired using the software Analyst 1.5.1 while the quantification was performed using the software MultiQuant 3.0.3 (Sciex, Massachusetts, USA). We calculated the contents of sucrose, trehalose (both from Sigma-Aldrich) and mannitol (Fluka) based on external curves. For glucose and

fructose, we determined the individual concentrations relative to the internal standards of $^{13}C_6$-glucose and $^{13}C_6$-fructose, respectively.

B vitamins

Analyses were performed by LC-MS/MS on an Agilent 1260 series HPLC system (Agilent Technologies) coupled to a tandem mass spectrometer QTRAP 6500 (SCIEX, Darmstadt, Germany). We achieved the chromatographic separation using a Zorbax Eclipse XDB-C18 column (50 x 4.6 mm, 1.8 µm, Agilent Technologies). As mobile phases A and B, we used water containing 0.05% formic acid and acetonitrile, respectively. The elution profile was as follow: 0–3.0 min, 0% B; 3.0–6.0 min, 0–80% B; 6.0–6.01 min, 80–100% B; 6.01–7.0 min, 100% B; and 7.1–10.0 min, 0% B. We kept the flow rate at 1.1 mL/min while the column temperature was maintained at 25°C. The mass spectrometer was equipped with a Turbo spray ion source operated in the positive ionization mode, where the ion spray voltage was maintained at 5,500 eV. The turbo gas temperature was set at 620°C and gas was nebulized at 60 psi, curtain gas at 40 psi, heating gas at 60 psi, while collision gas was set to “medium.” We operated the mass spectrometer in multiple reaction monitoring (MRM) mode (see Suppl. Table S4 for MRM table).

Identification of silver fir defenses

All petri dishes with fungi were prepared and incubated as described above for the nutritional profiling. After the plates were covered with fungus (or after max. of 14 d), fungal biomass was removed from the added piece of cellophane. Next, we collected medium samples from fungus-colonized locations. Both biomass and medium samples for each fungus were freeze-dried and methanolic extracts were prepared as described above.

We performed chromatography on an Agilent 1200 HPLC system (Agilent Technologies, Boeblingen, Germany). Here, the separation was achieved on an Agilent Zorbax Eclipse XDB-C18 column (50 x 4.6 mm, 1.8 µm; Agilent Technologies, Santa Clara, CA, USA). We employed formic acid (0.05%) in water and acetonitrile as mobile phases A and B, respectively. The elution profile was: 0.0-0.5 min, 5% B; 0.5-6.0 min, 5-37.4% B; 6.0-6.02 min, 37.4-80% B; 6.02-7.5 min, 80-100% B; 7.5-9.5 min, 100% B and 9.5-12 min 5% B. The flow rate for the mobile phase was set to 1.1 mL/min and the column temperature was maintained at 25°C.

The HPLC was coupled to an API 3200 tandem mass spectrometer (Applied Biosystems, Darmstadt, Germany) equipped with a Turbospray ion source, which was operated in the negative ionization mode. We optimized the instrument parameters by infusion experiments with pure standards. The ion spray voltage was maintained at -4500 eV and the turbo gas temperature was set at 500°C. Nebulizing gas was set at 60 psi, curtain gas at 30 psi, heating gas at 60 psi and collision gas at 4 psi. The mass spectrometer was operated in multiple reaction monitoring (MRM) mode, and details of the instrument parameters can be found in Suppl. Table S4. We maintained both Q1 and Q3 quadrupoles at unit resolution. Analyst 1.5 software (Applied Biosystems, Darmstadt, Germany) was used for data acquisition and processing. We quantified our target compounds based on external standard curves using dilution series of commercial standards (see Suppl. Table S5 for suppliers). Samples were normalized by the individual sample dry weight.

We compared the individual defenses identified in our silver fir phloem control medium (uncolonized by fungus) among the media in which the various fungi were grown. After fungal biomass was weighed, the data of the most abundant compounds in biomass and medium were first visualized by heatmaps as described above. Next, we performed a Principal Component Analysis (PCA) with the five most abundant

compounds in colonized culture medium using log1p-transformed and z-scaled data. Statistical differences were revealed by both global and pairwise PERMANOVAs as described above using log1p-transformed, but non-scaled data. Bray-Curtis distances were used to quantify dissimilarities, and *P* values from pairwise comparisons were adjusted for multiple testing using the Benjamini–Hochberg method. Further, we performed tests for multivariate homogeneity of group dispersions (beta-dispersion) with 999 permutations as described previously. Then, we compared the sum of the major identified compounds (total) in silver fir phloem in the presence of different fungi. Here, we generated boxplots using the R package “ggplot2” (21) and applied fitted GLMs to reveal significant differences (see above). All plots were modified using the software Adobe Illustrator (version CS5).

Inhibition assays

Solid PDA medium was supplemented with individual substances (gallic acid, vanillic acid, protocatechuic acid, catechin, shikimic acid, quinic acid; N = 6-7 per substance and fungus) with the applied concentrations based on our previous findings in silver fir phloem medium with and without fungal colonization (see Suppl. Table S5 for tested concentrations). Although gallic acid and vanillic acid were only present in low amounts in uncolonized silver fir phloem or were not detectable (in case of vanillic acid), they showed higher concentrations in the presence of certain fungi and were therefore included to our inhibition assays. Compounds were first dissolved in DMSO (Roth, Germany) and were added to medium after autoclaving. As a control, we used PDA that was supplemented with DMSO using the same concentration as above (final concentration: 0.25%). To start the experiment, a plug (Ø 6 mm) of pure actively growing mycelium from each tested fungus was inoculated on plates of freshly prepared medium. Here, all fungi were added at the same day on a circular piece of

sterilized cellophane covering the entire petri dish to ensure the complete separation of biomass and medium. Plates were incubated at 25°C and 60% humidity until one of the treatments (substances or control) for each individual fungus was completely overgrown. Then, the experiment was stopped for the respective fungus and all available biomass was removed and subsequently free-dried for 4 d to record the dry weight of fungal biomass.

To identify significant effects on fungal growth, as measured in mg dry weight for each of the six tested compounds, we applied a series of fitted GLMs, where we specifically focused on pairwise contrasts comparing individual fungal growth on the control with each of the tested compounds (see ref. (28) for further details). *P* values were adjusted for multiple testing using Dunnett´s correction. Data were visualized using boxplots applying the package "ggplot2" (21) and further modified using the software Adobe Illustrator (version CS5).

Depletion of individual compounds by tested fungi

The experimental setup including individual tested compounds, replicates, and applied concentrations was as described above. Here, we prepared separate controls for each examined compound (N = 6-7 per compound) without a fungal inoculation to allow comparison between the abundance of a substance with and without fungal growth. The experiment was stopped as soon as the individual plates of each fungus with each substance were completely colonized or after a max. of 14 d. Then, we collected medium samples from fungus-colonized locations and freeze-dried them for 4 d to record medium dry weight. Individual compounds were quantified as described above using methanolic extracts and targeted LC-MS/MS analyses.

To reveal differences in the depletion ability of the examined fungi with a fungus-free control, we first generated boxplots with the package "ggplot2" (21) and analyzed

data using fitted GLMs as described above. The software Adobe Illustrator (version CS5) was used for plot modifications.

References

1. Bodenhofer U, Bonatesta E, Horejš-Kainrath C, and Hochreiter S. msa: an R package for multiple sequence alignment. *Bioinformatics*. 2015. **31**(24): p. 3997–3999.
2. Sievers F, Wilm A, Dineen D, Gibson TJ, Karplus K, Li W, et al. Fast, scalable generation of high-quality protein multiple sequence alignments using Clustal Omega. *Molecular systems biology*. 2011. **7**(1): p. 539.
3. Katoh K and Standley DM. MAFFT multiple sequence alignment software version 7: improvements in performance and usability. *Molecular biology and evolution*. 2013. **30**(4): p. 772–780.
4. Larkin MA, Blackshields G, Brown NP, Chenna R, McGettigan PA, McWilliam H, et al. Clustal W and Clustal X version 2.0. *bioinformatics*. 2007. **23**(21): p. 2947–2948.
5. Castresana J. Selection of conserved blocks from multiple alignments for their use in phylogenetic analysis. *Molecular biology and evolution*. 2000. **17**(4): p. 540–552.
6. Schneider TD and Stephens RM. Sequence logos: a new way to display consensus sequences. *Nucleic acids research*. 1990. **18**(20): p. 6097–6100.
7. Shannon CE. A mathematical theory of communication. *The Bell system technical journal*. 1948. **27**(3): p. 379–423.
8. Yang Z. PAML 4: phylogenetic analysis by maximum likelihood. *Molecular biology and evolution*. 2007. **24**(8): p. 1586–1591.
9. Paradis E, Claude J, and Strimmer K. APE: analyses of phylogenetics and evolution in R language. *Bioinformatics*. 2004. **20**(2): p. 289–290.
10. Pages H, Aboyoun P, Gentleman R, DebRoy S, Pagès MH, DataImport D, et al. Package 'Biostrings'. *Bioconductor*. 2013. **18129**: p. 501.
11. Charif D, Lobry JR, Necsulea A, Palmeira L, Penel S, Perriere G, et al. Package 'seqinr'. *Biological Sequences Retrieval and Analysis*. 2017.
12. Schliep KP. phangorn: phylogenetic analysis in R. *Bioinformatics*. 2011. **27**(4): p. 592–593.
13. Hausser J, Strimmer K, and Strimmer MK. Package 'entropy'. *R Foundation for Statistical Computing: Vienna, Austria*. 2012.
14. Minh BQ, Schmidt HA, Chernomor O, Schrempf D, Woodhams MD, Von Haeseler A, et al. IQ-TREE 2: new models and efficient methods for phylogenetic inference in the genomic era. *Molecular biology and evolution*. 2020. **37**(5): p. 1530–1534.
15. Hoang DT, Chernomor O, Von Haeseler A, Minh BQ, and Vinh LS. UFBoot2: improving the ultrafast bootstrap approximation. *Molecular biology and evolution*. 2018. **35**(2): p. 518–522.
16. Paradis E, Blomberg S, Bolker B, Brown J, Claude J, Cuong HS, et al. Package 'ape'. *Analyses of phylogenetics and evolution, version*. 2019. **2**(4): p. 47.
17. Wickham H and Wickham MH. dplyr: A Grammar of Data Manipulation. *R package version 0.8.5*. 2020.

18. Wickham H, Averick M, Bryan J, Chang W, McGowan LDA, François R, et al. Welcome to the Tidyverse. *Journal of open source software*. 2019. **4**(43): p. 1686.
19. Wickham H, *stringr: simple, consistent wrappers for common string operations. R package version 1.4. 0*. 2019.
20. Yu G, Smith DK, Zhu H, Guan Y, and Lam TTY. ggtree: an R package for visualization and annotation of phylogenetic trees with their covariates and other associated data. *Methods in ecology and evolution*. 2017. **8**(1): p. 28–36.
21. Wickham H, *ggplot2: Elegant Graphics for Data Analysis*. 2016: Springer-Verlag New York.
22. Kassambara A and Mundt F. Factoextra: extract and visualize the results of multivariate data analyses. *CRAN: Contributed Packages*. 2016.
23. Pedersen TL, *ggforce: Accelerating “ggplot2.” R package version 0.3. 3*. 2020.
24. Lê S, Josse J, and Husson F. FactoMineR: an R package for multivariate analysis. *Journal of statistical software*. 2008. **25**: p. 1–18.
25. Neuwirth E. RColorBrewer: colorbrewer palettes. 2014.
26. Martinez Arbizu P. pairwiseAdonis: Pairwise multilevel comparison using adonis. R package. *Version 0.4. Available on URL: https://github. com/pmartinezarbizu/pairwiseAdonis*. 2020.
27. Oksanen J, Kindt R, and Simpson GL. vegan3d: static and dynamic 3D plots for the ‘vegan’package. *R package version*. 2017. **1**.
28. Lehenberger M, Pan Y, Ungerer S, Reichelt M, Pemp D, Paetz C, et al. Fungal symbiont of an ambrosia beetle possesses high nutrient content and suppresses competing fungi with antimicrobial compounds. *The ISME Journal*. 2025. **19**(1): p. wraf258.
29. Kolde R, *pheatmap: Pretty Heatmaps. R package version 1.0. 12*. 2019.
30. Wickham H and Vaughan D, *Girlich, M. tidyr: tidy messy data. R package version 1.3. 1*. 2024.
31. Wickham H. Reshaping data with the reshape package. *Journal of statistical software*. 2007. **21**: p. 1–20.
32. Crocoll C, Mirza N, Reichelt M, Gershenzon J, and Halkier BA. Optimization of engineered production of the glucoraphanin precursor dihomomethionine in Nicotiana benthamiana. *Frontiers in Bioengineering and Biotechnology*. 2016. **4**: p. 14.
