# Supplementary material for: The mutualistic fungi of the bark beetle Pityokteines vorontzowi are nutrient-rich and efficiently deplete their medium of fir chemical defenses
Source: ISME Commun. 2026 May 13;6(1):ycag131. doi: 10.1093/ismeco/ycag131 (PMC13245730; doi:10.1093/ismeco/ycag131)
Supplement: Supplementary_material_ycag131 [file supplementary_material_ycag131.zip › Suppl. Fig. S1.pdf]

Amount of free amino acids in tissue biomass (noml/mg)

noml/mg: 0, 20, 40, 60, 80, 100

	Ala	Ser	Pro	Val	Thr	Ile	Leu	Asp	Glu	Met	His	Phe	Arg	Tyr	Trp	Asn	Gln	GABA	Lys
A .grosmanniae	5.1	1.3	0.6	0.7	0.6	0.4	0.4	1.2	8.9	0.0	0.9	0.2	0.0	0.3	0.2	0.0	9.1	6.5	0.0
D. sulphureus	11.0	2.1	0.7	1.1	1.1	0.3	0.5	1.1	19.7	0.1	0.4	0.2	0.1	0.3	0.2	1.4	6.9	7.7	0.0
E. polonica	11.4	1.4	0.6	1.4	0.9	0.9	1.6	0.9	5.9	0.0	0.6	0.5	0.0	0.6	0.4	0.0	4.2	6.4	0.0
G. penicillata	32.1	3.5	2.5	3.9	3.7	2.1	4.2	1.3	20.6	0.0	1.4	0.7	0.1	0.8	0.8	0.1	26.7	17.5	0.0
O. bicolor	50.3	3.9	1.2	2.9	2.5	2.1	1.9	1.3	25.0	0.1	0.6	0.5	1.7	1.2	0.5	0.3	91.5	49.7	0.2
Geosmithia sp. F1	6.3	1.4	4.7	1.0	1.6	0.5	0.9	0.3	21.9	0.0	0.3	0.5	0.1	0.6	0.4	0.0	13.7	1.9	0.0
O. piceae	13.3	4.1	1.0	2.0	2.1	1.2	2.4	2.2	18.2	0.0	0.4	1.1	0.0	2.2	1.0	0.0	12.5	23.5	0.0
G. pseudormiticum	16.2	4.3	0.9	1.4	2.8	1.0	1.9	4.5	113.9	0.1	0.3	0.6	1.0	2.2	0.7	0.6	57.4	12.9	0.8
T. rugulosus	6.5	3.0	1.7	1.6	2.2	1.0	2.1	3.3	22.2	0.1	0.5	0.5	0.1	1.1	0.6	0.5	12.0	6.2	0.1
P. polonicum	5.4	3.9	1.7	1.4	2.2	0.6	1.4	4.8	40.7	0.1	0.5	0.6	0.4	4.9	8.4	0.8	30.4	1.5	0.1
Blastobotrys sp. F55	30.3	9.3	13.2	1.6	3.5	1.1	1.8	17.1	62.8	0.2	1.8	0.9	0.4	2.1	1.1	2.2	32.3	1.2	0.3
P. bialowiezense	5.7	4.5	2.3	1.9	4.1	1.0	2.1	4.6	49.0	0.2	0.7	1.1	0.1	1.6	0.5	0.9	41.2	7.4	0.1
G. fragrans	34.2	4.5	1.3	2.1	3.1	1.4	2.1	2.0	84.5	0.0	0.2	1.0	1.4	2.3	2.9	0.1	68.5	67.6	0.5
C. rollhanseniana	20.9	2.2	1.5	1.7	2.3	0.8	1.5	4.2	43.2	0.2	0.8	0.7	1.6	1.1	0.7	2.2	51.2	41.2	0.1
Cladosporium sp. F94	3.5	2.2	0.4	0.9	0.8	0.6	1.0	2.3	22.7	0.1	0.2	0.6	0.0	0.8	0.5	0.5	7.6	4.0	0.0
B. bassiana	3.1	0.9	7.0	0.7	0.7	0.3	0.4	1.3	19.6	0.0	0.2	0.2	0.0	0.5	0.2	0.2	1.4	0.2	0.0
T. lixii	31.4	10.2	5.0	8.7	6.1	6.1	13.6	1.8	4.7	0.2	28.6	4.6	0.7	3.9	1.3	1.2	15.7	51.6	0.3
Phloem medium (Control)	0.2	0.1	0.0	0.0	0.0	0.0	0.0	0.1	0.3	0.0	0.2	0.0	0.0	0.0	0.0	0.1	0.0	2.3	0.0
