# Supplementary material for: The mutualistic fungi of the bark beetle Pityokteines vorontzowi are nutrient-rich and efficiently deplete their medium of fir chemical defenses
Source: ISME Commun. 2026 May 13;6(1):ycag131. doi: 10.1093/ismeco/ycag131 (PMC13245730; doi:10.1093/ismeco/ycag131)

Amount of soluble sugars in tissue biomass (µg/mg)

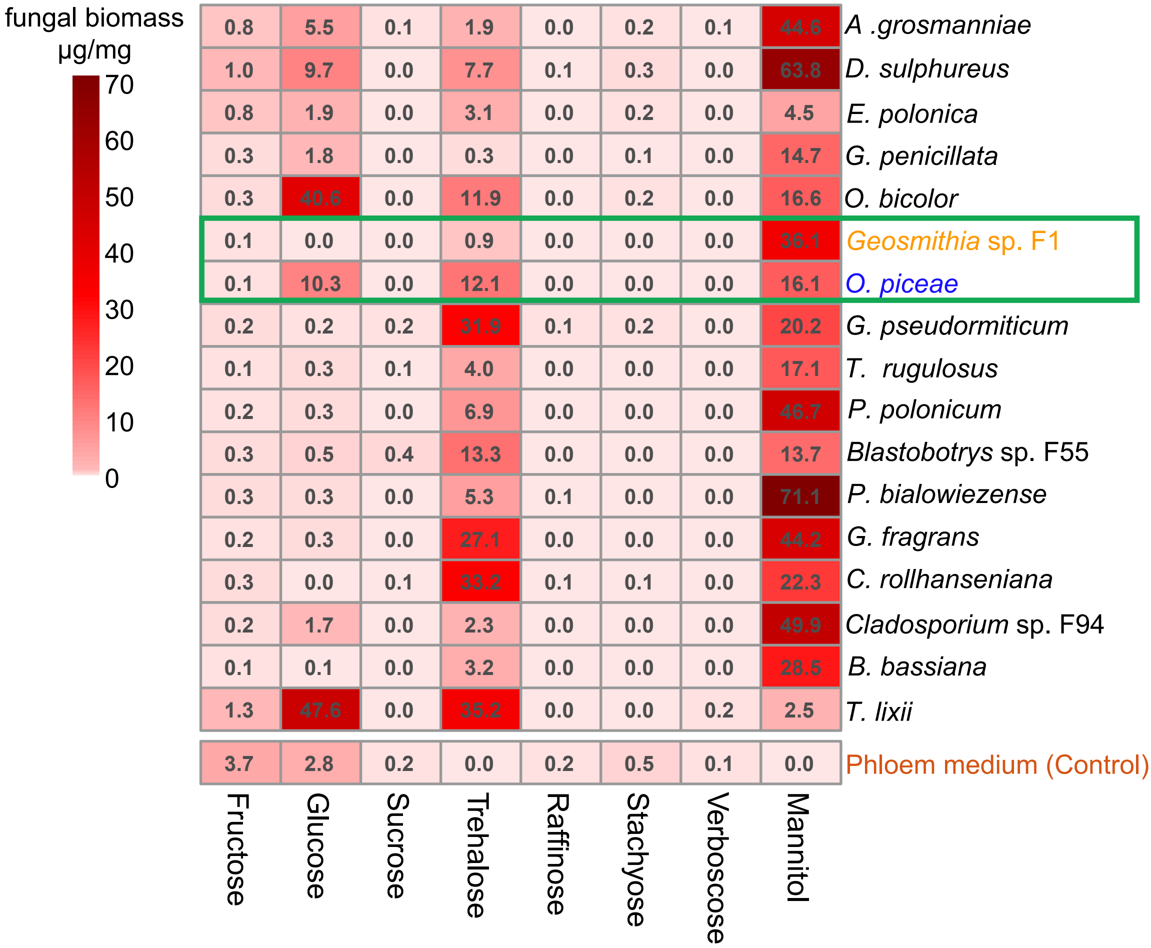

Supplement: Supplementary_material_ycag131 [file supplementary_material_ycag131.zip › Suppl. Fig. S2.pdf]
