# Supplementary material for: The mutualistic fungi of the bark beetle Pityokteines vorontzowi are nutrient-rich and efficiently deplete their medium of fir chemical defenses
Source: ISME Commun. 2026 May 13;6(1):ycag131. doi: 10.1093/ismeco/ycag131 (PMC13245730; doi:10.1093/ismeco/ycag131)
Supplement: Supplementary_material_ycag131 [file supplementary_material_ycag131.zip › Suppl. Fig. S3.pdf]

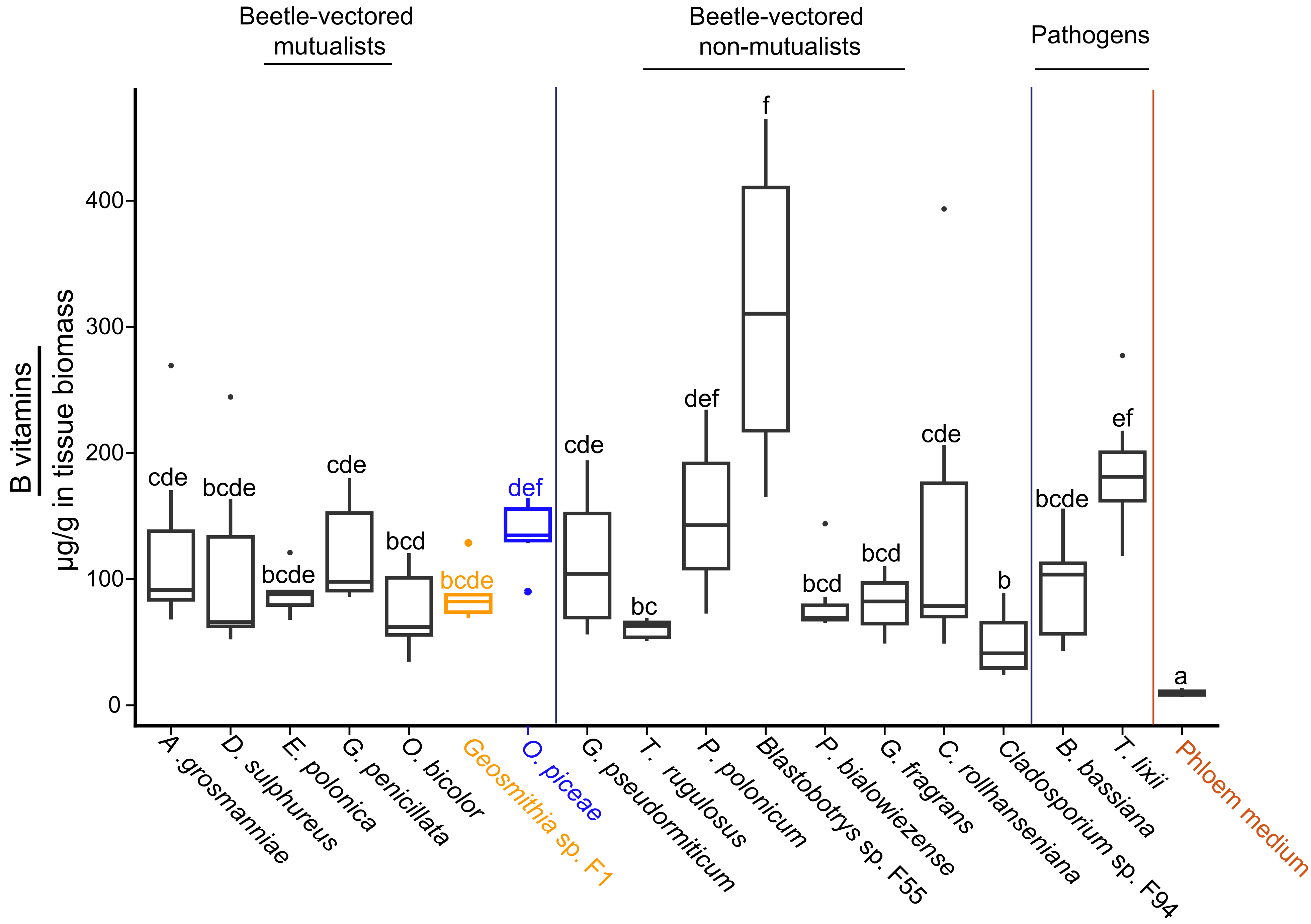

Beetle-vectored mutualists
Beetle-vectored non-mutualists
Pathogens
B vitamins
μg/g in tissue biomass
0
100
200
300
400
cde
bcde
bcde
cde
bcd
bcde
def
cde
bc
def
f
bcd
bcd
cde
b
bcde
ef
a
A .grosmanniae
D. sulphureus
E. polonica
G. penicillata
O. bicolor
Geosmithia sp. F1
O. piceae
G. pseudormiticum
T. rugulosus
P. polonicum
Blastobotrys sp. F55
P. bialowiezense
G. fragrans
C. rollhanseniana
Cladosporium sp. F94
B. bassiana
T. lixii
Phloem medium
