# Supplementary material for: The mutualistic fungi of the bark beetle Pityokteines vorontzowi are nutrient-rich and efficiently deplete their medium of fir chemical defenses
Source: ISME Commun. 2026 May 13;6(1):ycag131. doi: 10.1093/ismeco/ycag131 (PMC13245730; doi:10.1093/ismeco/ycag131)
Supplement: Supplementary_material_ycag131 [file supplementary_material_ycag131.zip › Suppl. Fig. S5.pdf]

Amount in tissue biomass (µg/g)

µg/g: 2000, 1500, 1000, 500, 0

	Protocatechuic acid	Vanillic acid	Gallic acid	Catechin	Shikimic acid	Quinic acid
A .grosmanniae	34.5	27.1	8.2	8.9	47.7	260.6
D. sulphureus	124.5	38.4	6.6	16.5	106.1	897.6
E. polonica	34.4	0.0	1.1	8.1	16.1	22.2
G. penicillata	19.8	0.0	2.8	4.2	83.5	557.9
O. bicolor	14.1	0.0	1.9	11.4	14.5	629.8
Geosmithia sp. F1	4.4	2.6	1.7	3.8	8.1	195.3
O. piceae	34.3	2.6	0.8	3.2	17.3	18.4
G. pseudormiticum	9.3	0.0	15.5	43.0	15.5	72.7
T. rugulosus	0.9	0.0	0.7	6.9	3.0	122.9
P. polonicum	3.1	0.0	4.2	0.0	0.0	0.0
Blastobotrys sp. F55	196.8	0.0	328.7	58.3	147.6	852.5
P. bialowiezense	2.6	1.1	3.9	7.2	2.2	56.6
G. fragrans	0.2	0.7	0.4	0.5	1.6	76.0
C. rollhanseniana	3.5	4.4	3.4	54.5	66.0	2301.2
Cladosporium sp. F94	73.1	5.9	3.1	25.9	27.5	35.3
B. bassiana	4.1	3.6	2.2	1.2	54.8	353.2
T. lixii	22.2	0.0	0.0	12.1	48.1	229.5
Phloem medium (Control)	229.4	0.0	35.2	199.4	198.1	804.4
