# Supplementary material for: The mutualistic fungi of the bark beetle Pityokteines vorontzowi are nutrient-rich and efficiently deplete their medium of fir chemical defenses
Source: ISME Commun. 2026 May 13;6(1):ycag131. doi: 10.1093/ismeco/ycag131 (PMC13245730; doi:10.1093/ismeco/ycag131)
Supplement: Supplementary_material_ycag131 [file supplementary_material_ycag131.zip › Suppl. Table S3.docx]

**Overview statistics and p-values**

**ID** **Fungus**

| F1 | *Geosmithia* sp. |
| --- | --- |
| F2 | *Ophiostoma piceae* |
| F5 | *Graphilbum pseudormiticum* |
| F31 | *Talaromyces rrugulosus* |
| F39 | *Penicillium brevicompactum* |
| F55 | *Blastobotrys* sp. |
| F54B | *Penicillium bialowiezense* |
| F58 | *Graphilbum fragrans* |
| F83 | *Ceratocystiopsis rollhanseniana* |
| F94 | *Cladosporium* sp. |
| F26 | *Trichoderma lixii* |
| F66 | *Beauveria bassiana* |
| P331 | *Endoconidiophora polonica* |
| P188 | *Grosmania penicillata* |
| 10FK1 | *Ophiostoma bicolor* |
| P159 | *Dryadomyces sulphureus* |
| P339 | *Ambrosiella grosmanniae* |

**ns - P > 0.05, * - P < 0.05, ** - P < 0.01, *** - P < 0.001**

If necessary, data was either log or square root (sqrt) transformed. We added this information by providing the respective R code.

Statistical analyses are sorted by the respective figure in this supplementary document.

**Nutritional analyses (Fig. 2)**

**PERMANOVA**

Globale PERMANOVA:

Permutation test for adonis under reduced model

Permutation: free

Number of permutations: 999

adonis2(formula = dist_final ~ Sample, data = data, permutations = 999)

Df SumOfSqs R2 F Pr(>F)

Model 17 6.0745 0.92722 77.186 0.001 ***

Residual 103 0.4768 0.07278

Total 120 6.5513 1.00000

---

Signif. codes: 0 ‘***’ 0.001 ‘**’ 0.01 ‘*’ 0.05 ‘.’ 0.1 ‘ ’ 1

See Suppl. Table S3a for pairwise comparison.

**Nutritional analyses (Fig. 3 and Suppl. Fig. S3) Total nutrients**

**Total soluble sugars**

| Modell |
| --- |
| Normal_log |

| Resid. Df | Resid. Dev | Df | Deviance | Pr(>Chi) | p.value_formatted | Significance |
| --- | --- | --- | --- | --- | --- | --- |
| 120 | 66.48166 |  |  |  | NA |  |
| 103 | 6.68695 | 17 | 59.79471 | 6.9E-185 | 6.8542183167e-185 | *** |

| contrast | estimate | SE | df | t.ratio | p.value | p.value | Significance |
| --- | --- | --- | --- | --- | --- | --- | --- |
| 1_339B - 2_159B | -0.43369 | 0.136195 | 103 | -3.18431 | 0.143939 | 1.4393885648e-01 |  |
| 1_339B - 3_331B | 1.638726 | 0.136195 | 103 | 12.0322 | 1.5E-14 | 1.4988010832e-14 | *** |
| 1_339B - 4_188B | 1.136475 | 0.136195 | 103 | 8.344467 | 5.24E-11 | 5.2442605813e-11 | *** |
| 1_339B - 5_O.bicolor | -0.27141 | 0.136195 | 103 | -1.99279 | 0.865701 | 8.6570128609e-01 |  |
| 1_339B - 6_F1 | 0.357456 | 0.141756 | 103 | 2.52162 | 0.520051 | 5.2005068058e-01 |  |
| 1_339B - 7_F2 | 0.336264 | 0.136195 | 103 | 2.468985 | 0.558718 | 5.5871771991e-01 |  |
| 1_339B - 8_F5 | 0.01815 | 0.141756 | 103 | 0.128037 | 1 | 1.0000000000e+00 |  |
| 1_339B - 9_F31 | 0.896932 | 0.136195 | 103 | 6.585644 | 2.92E-07 | 2.9179344685e-07 | *** |
| 1_339B - 91_F39 | -0.17105 | 0.141756 | 103 | -1.20663 | 0.999073 | 9.9907253031e-01 |  |
| 1_339B - 92_F55 | 0.93632 | 0.141756 | 103 | 6.605139 | 2.66E-07 | 2.6616087723e-07 | *** |
| 1_339B - 93_F54 | -0.37702 | 0.136195 | 103 | -2.76822 | 0.349353 | 3.4935298711e-01 |  |
| 1_339B - 94_F58 | -0.29391 | 0.136195 | 103 | -2.15803 | 0.77538 | 7.7538004982e-01 |  |
| 1_339B - 95_F83 | -0.05878 | 0.136195 | 103 | -0.43157 | 1 | 9.9999999980e-01 |  |
| 1_339B - 96_F94 | -0.01408 | 0.136195 | 103 | -0.10336 | 1 | 1.0000000000e+00 |  |
| 1_339B - 97_F66 | 0.548512 | 0.136195 | 103 | 4.0274 | 0.012221 | 1.2220872335e-02 | * |
| 1_339B - 98_F26B | -0.47101 | 0.136195 | 103 | -3.45835 | 0.07049 | 7.0490353022e-02 | . |
| 1_339B - 99_Control | 1.985803 | 0.141756 | 103 | 14.00857 | 0 | 0.0000000000e+00 | *** |
| 2_159B - 3_331B | 2.072413 | 0.136195 | 103 | 15.21651 | 0 | 0.0000000000e+00 | *** |
| 2_159B - 4_188B | 1.570163 | 0.136195 | 103 | 11.52878 | 4.72E-14 | 4.7184478547e-14 | *** |
| 2_159B - 5_O.bicolor | 0.16228 | 0.136195 | 103 | 1.191523 | 0.999207 | 9.9920704895e-01 |  |
| 2_159B - 6_F1 | 0.791143 | 0.141756 | 103 | 5.581007 | 2.81E-05 | 2.8073774621e-05 | *** |
| 2_159B - 7_F2 | 0.769951 | 0.136195 | 103 | 5.653295 | 2.05E-05 | 2.0474475638e-05 | *** |
| 2_159B - 8_F5 | 0.451837 | 0.141756 | 103 | 3.187424 | 0.142854 | 1.4285364017e-01 |  |
| 2_159B - 9_F31 | 1.33062 | 0.136195 | 103 | 9.769955 | 1.99E-13 | 1.9928503292e-13 | *** |
| 2_159B - 91_F39 | 0.26264 | 0.141756 | 103 | 1.852759 | 0.922025 | 9.2202526788e-01 |  |
| 2_159B - 92_F55 | 1.370008 | 0.141756 | 103 | 9.664526 | 2.19E-13 | 2.1904700276e-13 | *** |
| 2_159B - 93_F54 | 0.05667 | 0.136195 | 103 | 0.416094 | 1 | 9.9999999989e-01 |  |
| 2_159B - 94_F58 | 0.139775 | 0.136195 | 103 | 1.026284 | 0.999887 | 9.9988663004e-01 |  |
| 2_159B - 95_F83 | 0.374909 | 0.136195 | 103 | 2.752737 | 0.35924 | 3.5924000777e-01 |  |
| 2_159B - 96_F94 | 0.41961 | 0.136195 | 103 | 3.08095 | 0.18367 | 1.8367009268e-01 |  |
| 2_159B - 97_F66 | 0.982199 | 0.136195 | 103 | 7.211711 | 1.45E-08 | 1.4499588419e-08 | *** |
| 2_159B - 98_F26B | -0.03732 | 0.136195 | 103 | -0.27404 | 1 | 1.0000000000e+00 |  |
| 2_159B - 99_Control | 2.41949 | 0.141756 | 103 | 17.06795 | 0 | 0.0000000000e+00 | *** |
| 3_331B - 4_188B | -0.50225 | 0.136195 | 103 | -3.68773 | 0.036212 | 3.6212191974e-02 | * |
| 3_331B - 5_O.bicolor | -1.91013 | 0.136195 | 103 | -14.025 | 0 | 0.0000000000e+00 | *** |
| 3_331B - 6_F1 | -1.28127 | 0.141756 | 103 | -9.03854 | 1.7E-12 | 1.7036372313e-12 | *** |
| 3_331B - 7_F2 | -1.30246 | 0.136195 | 103 | -9.56321 | 2.64E-13 | 2.6423307986e-13 | *** |
| 3_331B - 8_F5 | -1.62058 | 0.141756 | 103 | -11.4321 | 5.36E-14 | 5.3623772089e-14 | *** |
| 3_331B - 9_F31 | -0.74179 | 0.136195 | 103 | -5.44655 | 5.02E-05 | 5.0183850252e-05 | *** |
| 3_331B - 91_F39 | -1.80977 | 0.141756 | 103 | -12.7668 | 0 | 0.0000000000e+00 | *** |
| 3_331B - 92_F55 | -0.70241 | 0.141756 | 103 | -4.95502 | 0.000389 | 3.8856489199e-04 | *** |
| 3_331B - 93_F54 | -2.01574 | 0.136195 | 103 | -14.8004 | 0 | 0.0000000000e+00 | *** |
| 3_331B - 94_F58 | -1.93264 | 0.136195 | 103 | -14.1902 | 0 | 0.0000000000e+00 | *** |
| 3_331B - 95_F83 | -1.6975 | 0.136195 | 103 | -12.4638 | 2.55E-15 | 2.5535129566e-15 | *** |
| 3_331B - 96_F94 | -1.6528 | 0.136195 | 103 | -12.1356 | 1.25E-14 | 1.2545520178e-14 | *** |
| 3_331B - 97_F66 | -1.09021 | 0.136195 | 103 | -8.00479 | 2.88E-10 | 2.8823043952e-10 | *** |
| 3_331B - 98_F26B | -2.10974 | 0.136195 | 103 | -15.4905 | 0 | 0.0000000000e+00 | *** |
| 3_331B - 99_Control | 0.347077 | 0.141756 | 103 | 2.448407 | 0.573868 | 5.7386789268e-01 |  |
| 4_188B - 5_O.bicolor | -1.40788 | 0.136195 | 103 | -10.3373 | 1.4E-13 | 1.3999912341e-13 | *** |
| 4_188B - 6_F1 | -0.77902 | 0.141756 | 103 | -5.49548 | 4.07E-05 | 4.0660545441e-05 | *** |
| 4_188B - 7_F2 | -0.80021 | 0.136195 | 103 | -5.87548 | 7.65E-06 | 7.6531804947e-06 | *** |
| 4_188B - 8_F5 | -1.11833 | 0.141756 | 103 | -7.88907 | 5.14E-10 | 5.1369897225e-10 | *** |
| 4_188B - 9_F31 | -0.23954 | 0.136195 | 103 | -1.75882 | 0.949257 | 9.4925696681e-01 |  |
| 4_188B - 91_F39 | -1.30752 | 0.141756 | 103 | -9.22373 | 7.61E-13 | 7.6061379417e-13 | *** |
| 4_188B - 92_F55 | -0.20016 | 0.141756 | 103 | -1.41197 | 0.99415 | 9.9415019765e-01 |  |
| 4_188B - 93_F54 | -1.51349 | 0.136195 | 103 | -11.1127 | 8.12E-14 | 8.1157303100e-14 | *** |
| 4_188B - 94_F58 | -1.43039 | 0.136195 | 103 | -10.5025 | 1.29E-13 | 1.2889689316e-13 | *** |
| 4_188B - 95_F83 | -1.19525 | 0.136195 | 103 | -8.77604 | 6.04E-12 | 6.0367266741e-12 | *** |
| 4_188B - 96_F94 | -1.15055 | 0.136195 | 103 | -8.44783 | 3.12E-11 | 3.1189939520e-11 | *** |
| 4_188B - 97_F66 | -0.58796 | 0.136195 | 103 | -4.31707 | 0.004458 | 4.4582672393e-03 | ** |
| 4_188B - 98_F26B | -1.60749 | 0.136195 | 103 | -11.8028 | 2.79E-14 | 2.7866597918e-14 | *** |
| 4_188B - 99_Control | 0.849328 | 0.141756 | 103 | 5.991461 | 4.54E-06 | 4.5431966123e-06 | *** |
| 5_O.bicolor - 6_F1 | 0.628863 | 0.141756 | 103 | 4.436228 | 0.002888 | 2.8877221205e-03 | ** |
| 5_O.bicolor - 7_F2 | 0.607671 | 0.136195 | 103 | 4.461773 | 0.002627 | 2.6273881986e-03 | ** |
| 5_O.bicolor - 8_F5 | 0.289558 | 0.141756 | 103 | 2.042646 | 0.841034 | 8.4103439637e-01 |  |
| 5_O.bicolor - 9_F31 | 1.16834 | 0.136195 | 103 | 8.578432 | 1.62E-11 | 1.6181278539e-11 | *** |
| 5_O.bicolor - 91_F39 | 0.100361 | 0.141756 | 103 | 0.707981 | 0.999999 | 9.9999947617e-01 |  |
| 5_O.bicolor - 92_F55 | 1.207728 | 0.141756 | 103 | 8.519748 | 2.17E-11 | 2.1729840149e-11 | *** |
| 5_O.bicolor - 93_F54 | -0.10561 | 0.136195 | 103 | -0.77543 | 0.999998 | 9.9999793088e-01 |  |
| 5_O.bicolor - 94_F58 | -0.0225 | 0.136195 | 103 | -0.16524 | 1 | 1.0000000000e+00 |  |
| 5_O.bicolor - 95_F83 | 0.21263 | 0.136195 | 103 | 1.561214 | 0.983215 | 9.8321470752e-01 |  |
| 5_O.bicolor - 96_F94 | 0.257331 | 0.136195 | 103 | 1.889427 | 0.909136 | 9.0913569550e-01 |  |
| 5_O.bicolor - 97_F66 | 0.81992 | 0.136195 | 103 | 6.020188 | 3.99E-06 | 3.9895333562e-06 | *** |
| 5_O.bicolor - 98_F26B | -0.1996 | 0.136195 | 103 | -1.46556 | 0.991247 | 9.9124735666e-01 |  |
| 5_O.bicolor - 99_Control | 2.257211 | 0.141756 | 103 | 15.92317 | 0 | 0.0000000000e+00 | *** |
| 6_F1 - 7_F2 | -0.02119 | 0.141756 | 103 | -0.1495 | 1 | 1.0000000000e+00 |  |
| 6_F1 - 8_F5 | -0.33931 | 0.147107 | 103 | -2.30651 | 0.676687 | 6.7668713515e-01 |  |
| 6_F1 - 9_F31 | 0.539477 | 0.141756 | 103 | 3.805662 | 0.025154 | 2.5153734682e-02 | * |
| 6_F1 - 91_F39 | -0.5285 | 0.147107 | 103 | -3.59263 | 0.048068 | 4.8068249693e-02 | * |
| 6_F1 - 92_F55 | 0.578865 | 0.147107 | 103 | 3.934978 | 0.016604 | 1.6603691793e-02 | * |
| 6_F1 - 93_F54 | -0.73447 | 0.141756 | 103 | -5.18124 | 0.000154 | 1.5390083968e-04 | *** |
| 6_F1 - 94_F58 | -0.65137 | 0.141756 | 103 | -4.59498 | 0.001593 | 1.5931565036e-03 | ** |
| 6_F1 - 95_F83 | -0.41623 | 0.141756 | 103 | -2.93626 | 0.251867 | 2.5186682522e-01 |  |
| 6_F1 - 96_F94 | -0.37153 | 0.141756 | 103 | -2.62093 | 0.448472 | 4.4847249059e-01 |  |
| 6_F1 - 97_F66 | 0.191056 | 0.141756 | 103 | 1.347781 | 0.99653 | 9.9652966376e-01 |  |
| 6_F1 - 98_F26B | -0.82847 | 0.141756 | 103 | -5.84429 | 8.8E-06 | 8.7975690405e-06 | *** |
| 6_F1 - 99_Control | 1.628347 | 0.147107 | 103 | 11.0691 | 8.43E-14 | 8.4265927569e-14 | *** |
| 7_F2 - 8_F5 | -0.31811 | 0.141756 | 103 | -2.24409 | 0.71975 | 7.1974978946e-01 |  |
| 7_F2 - 9_F31 | 0.560669 | 0.136195 | 103 | 4.116659 | 0.009024 | 9.0243576646e-03 | ** |
| 7_F2 - 91_F39 | -0.50731 | 0.141756 | 103 | -3.57875 | 0.050055 | 5.0055463982e-02 | . |
| 7_F2 - 92_F55 | 0.600057 | 0.141756 | 103 | 4.233015 | 0.006016 | 6.0163771782e-03 | ** |
| 7_F2 - 93_F54 | -0.71328 | 0.136195 | 103 | -5.2372 | 0.000122 | 1.2186244548e-04 | *** |
| 7_F2 - 94_F58 | -0.63018 | 0.136195 | 103 | -4.62701 | 0.00141 | 1.4100160793e-03 | ** |
| 7_F2 - 95_F83 | -0.39504 | 0.136195 | 103 | -2.90056 | 0.270984 | 2.7098371536e-01 |  |
| 7_F2 - 96_F94 | -0.35034 | 0.136195 | 103 | -2.57235 | 0.483167 | 4.8316674434e-01 |  |
| 7_F2 - 97_F66 | 0.212249 | 0.136195 | 103 | 1.558416 | 0.983512 | 9.8351233290e-01 |  |
| 7_F2 - 98_F26B | -0.80727 | 0.136195 | 103 | -5.92733 | 6.07E-06 | 6.0655243429e-06 | *** |
| 7_F2 - 99_Control | 1.649539 | 0.141756 | 103 | 11.63644 | 3.65E-14 | 3.6526337510e-14 | *** |
| 8_F5 - 9_F31 | 0.878782 | 0.141756 | 103 | 6.199245 | 1.76E-06 | 1.7628584155e-06 | *** |
| 8_F5 - 91_F39 | -0.1892 | 0.147107 | 103 | -1.28612 | 0.997989 | 9.9798929825e-01 |  |
| 8_F5 - 92_F55 | 0.91817 | 0.147107 | 103 | 6.241492 | 1.45E-06 | 1.4515183295e-06 | *** |
| 8_F5 - 93_F54 | -0.39517 | 0.141756 | 103 | -2.78765 | 0.33714 | 3.3713985120e-01 |  |
| 8_F5 - 94_F58 | -0.31206 | 0.141756 | 103 | -2.2014 | 0.747958 | 7.4795805746e-01 |  |
| 8_F5 - 95_F83 | -0.07693 | 0.141756 | 103 | -0.54268 | 1 | 9.9999999180e-01 |  |
| 8_F5 - 96_F94 | -0.03223 | 0.141756 | 103 | -0.22734 | 1 | 1.0000000000e+00 |  |
| 8_F5 - 97_F66 | 0.530362 | 0.141756 | 103 | 3.741364 | 0.030736 | 3.0735993820e-02 | * |
| 8_F5 - 98_F26B | -0.48916 | 0.141756 | 103 | -3.45071 | 0.071997 | 7.1997175321e-02 | . |
| 8_F5 - 99_Control | 1.967653 | 0.147107 | 103 | 13.37561 | 0 | 0.0000000000e+00 | *** |
| 9_F31 - 91_F39 | -1.06798 | 0.141756 | 103 | -7.53391 | 2.99E-09 | 2.9898937770e-09 | *** |
| 9_F31 - 92_F55 | 0.039388 | 0.141756 | 103 | 0.277857 | 1 | 1.0000000000e+00 |  |
| 9_F31 - 93_F54 | -1.27395 | 0.136195 | 103 | -9.35386 | 4.71E-13 | 4.7062354014e-13 | *** |
| 9_F31 - 94_F58 | -1.19084 | 0.136195 | 103 | -8.74367 | 7.09E-12 | 7.0915495698e-12 | *** |
| 9_F31 - 95_F83 | -0.95571 | 0.136195 | 103 | -7.01722 | 3.72E-08 | 3.7220407756e-08 | *** |
| 9_F31 - 96_F94 | -0.91101 | 0.136195 | 103 | -6.689 | 1.79E-07 | 1.7899215987e-07 | *** |
| 9_F31 - 97_F66 | -0.34842 | 0.136195 | 103 | -2.55824 | 0.493365 | 4.9336484803e-01 |  |
| 9_F31 - 98_F26B | -1.36794 | 0.136195 | 103 | -10.044 | 1.61E-13 | 1.6142642778e-13 | *** |
| 9_F31 - 99_Control | 1.088871 | 0.141756 | 103 | 7.681284 | 1.44E-09 | 1.4430551198e-09 | *** |
| 91_F39 - 92_F55 | 1.107367 | 0.147107 | 103 | 7.527607 | 3.08E-09 | 3.0842602916e-09 | *** |
| 91_F39 - 93_F54 | -0.20597 | 0.141756 | 103 | -1.45299 | 0.992016 | 9.9201616919e-01 |  |
| 91_F39 - 94_F58 | -0.12287 | 0.141756 | 103 | -0.86674 | 0.999989 | 9.9998937565e-01 |  |
| 91_F39 - 95_F83 | 0.112269 | 0.141756 | 103 | 0.791985 | 0.999997 | 9.9999716619e-01 |  |
| 91_F39 - 96_F94 | 0.15697 | 0.141756 | 103 | 1.107322 | 0.999689 | 9.9968883341e-01 |  |
| 91_F39 - 97_F66 | 0.719559 | 0.141756 | 103 | 5.076029 | 0.000238 | 2.3759752692e-04 | *** |
| 91_F39 - 98_F26B | -0.29996 | 0.141756 | 103 | -2.11604 | 0.80057 | 8.0057030473e-01 |  |
| 91_F39 - 99_Control | 2.15685 | 0.147107 | 103 | 14.66173 | 0 | 0.0000000000e+00 | *** |
| 92_F55 - 93_F54 | -1.31334 | 0.141756 | 103 | -9.26476 | 6.51E-13 | 6.5103478164e-13 | *** |
| 92_F55 - 94_F58 | -1.23023 | 0.141756 | 103 | -8.6785 | 9.81E-12 | 9.8135943816e-12 | *** |
| 92_F55 - 95_F83 | -0.9951 | 0.141756 | 103 | -7.01978 | 3.68E-08 | 3.6762757838e-08 | *** |
| 92_F55 - 96_F94 | -0.9504 | 0.141756 | 103 | -6.70444 | 1.66E-07 | 1.6634868294e-07 | *** |
| 92_F55 - 97_F66 | -0.38781 | 0.141756 | 103 | -2.73574 | 0.370256 | 3.7025590752e-01 |  |
| 92_F55 - 98_F26B | -1.40733 | 0.141756 | 103 | -9.92781 | 1.63E-13 | 1.6331380692e-13 | *** |
| 92_F55 - 99_Control | 1.049483 | 0.147107 | 103 | 7.13412 | 2.11E-08 | 2.1140954432e-08 | *** |
| 93_F54 - 94_F58 | 0.083105 | 0.136195 | 103 | 0.610191 | 1 | 9.9999994754e-01 |  |
| 93_F54 - 95_F83 | 0.318239 | 0.136195 | 103 | 2.336643 | 0.655292 | 6.5529222432e-01 |  |
| 93_F54 - 96_F94 | 0.36294 | 0.136195 | 103 | 2.664856 | 0.417831 | 4.1783089822e-01 |  |
| 93_F54 - 97_F66 | 0.92553 | 0.136195 | 103 | 6.795617 | 1.08E-07 | 1.0779311754e-07 | *** |
| 93_F54 - 98_F26B | -0.09399 | 0.136195 | 103 | -0.69013 | 1 | 9.9999964556e-01 |  |
| 93_F54 - 99_Control | 2.36282 | 0.141756 | 103 | 16.66818 | 0 | 0.0000000000e+00 | *** |
| 94_F58 - 95_F83 | 0.235134 | 0.136195 | 103 | 1.726453 | 0.956823 | 9.5682255786e-01 |  |
| 94_F58 - 96_F94 | 0.279835 | 0.136195 | 103 | 2.054665 | 0.834738 | 8.3473811498e-01 |  |
| 94_F58 - 97_F66 | 0.842425 | 0.136195 | 103 | 6.185426 | 1.88E-06 | 1.8783037986e-06 | *** |
| 94_F58 - 98_F26B | -0.1771 | 0.136195 | 103 | -1.30032 | 0.997711 | 9.9771070097e-01 |  |
| 94_F58 - 99_Control | 2.279715 | 0.141756 | 103 | 16.08193 | 0 | 0.0000000000e+00 | *** |
| 95_F83 - 96_F94 | 0.044701 | 0.136195 | 103 | 0.328213 | 1 | 1.0000000000e+00 |  |
| 95_F83 - 97_F66 | 0.60729 | 0.136195 | 103 | 4.458974 | 0.002655 | 2.6547900773e-03 | ** |
| 95_F83 - 98_F26B | -0.41223 | 0.136195 | 103 | -3.02677 | 0.207459 | 2.0745863817e-01 |  |
| 95_F83 - 99_Control | 2.044581 | 0.141756 | 103 | 14.42321 | 0 | 0.0000000000e+00 | *** |
| 96_F94 - 97_F66 | 0.562589 | 0.136195 | 103 | 4.130761 | 0.008597 | 8.5968310284e-03 | ** |
| 96_F94 - 98_F26B | -0.45693 | 0.136195 | 103 | -3.35499 | 0.093297 | 9.3297133494e-02 | . |
| 96_F94 - 99_Control | 1.99988 | 0.141756 | 103 | 14.10787 | 0 | 0.0000000000e+00 | *** |
| 97_F66 - 98_F26B | -1.01952 | 0.136195 | 103 | -7.48575 | 3.79E-09 | 3.7904992478e-09 | *** |
| 97_F66 - 99_Control | 1.437291 | 0.141756 | 103 | 10.13916 | 1.5E-13 | 1.4954704142e-13 | *** |
| 98_F26B - 99_Control | 2.456813 | 0.141756 | 103 | 17.33124 | 0 | 0.0000000000e+00 | *** |

| Sample | emmean | SE | df | lower.CL | upper.CL | .group |
| --- | --- | --- | --- | --- | --- | --- |
| 99_Control | 1.978841 | 0.104021 | 103 | 1.660819 | 2.296862 | a |
| 3_331B | 2.325918 | 0.096304 | 103 | 2.031487 | 2.620349 | a |
| 4_188B | 2.828168 | 0.096304 | 103 | 2.533738 | 3.122599 | b |
| 92_F55 | 3.028323 | 0.104021 | 103 | 2.710302 | 3.346345 | bc |
| 9_F31 | 3.067711 | 0.096304 | 103 | 2.773281 | 3.362142 | bc |
| 97_F66 | 3.416132 | 0.096304 | 103 | 3.121701 | 3.710562 | cd |
| 6_F1 | 3.607188 | 0.104021 | 103 | 3.289167 | 3.92521 | de |
| 7_F2 | 3.62838 | 0.096304 | 103 | 3.333949 | 3.922811 | def |
| 8_F5 | 3.946494 | 0.104021 | 103 | 3.628472 | 4.264515 | efg |
| 1_339B | 3.964644 | 0.096304 | 103 | 3.670213 | 4.259074 | efg |
| 96_F94 | 3.978721 | 0.096304 | 103 | 3.68429 | 4.273152 | efg |
| 95_F83 | 4.023422 | 0.096304 | 103 | 3.728991 | 4.317853 | efg |
| 91_F39 | 4.135691 | 0.104021 | 103 | 3.817669 | 4.453712 | fg |
| 5_O.bicolor | 4.236052 | 0.096304 | 103 | 3.941621 | 4.530482 | g |
| 94_F58 | 4.258556 | 0.096304 | 103 | 3.964125 | 4.552987 | g |
| 93_F54 | 4.341661 | 0.096304 | 103 | 4.04723 | 4.636092 | g |
| 2_159B | 4.398331 | 0.096304 | 103 | 4.1039 | 4.692762 | g |
| 98_F26B | 4.435653 | 0.096304 | 103 | 4.141223 | 4.730084 | g |

**Total free amino acids**

| Modell |
| --- |
| Normal_log |

| Resid. Df | Resid. Dev | Df | Deviance | Pr(>Chi) | p.value_formatted | Significance |
| --- | --- | --- | --- | --- | --- | --- |
| 120 | 128.9471 |  |  |  | NA |  |
| 103 | 10.36632 | 17 | 118.5808 | 6.1E-240 | 6.1268280110e-240 | *** |

| contrast | estimate | SE | df | t.ratio | p.value | p.value | Significance |
| --- | --- | --- | --- | --- | --- | --- | --- |
| 1_339B - 2_159B | -0.53761 | 0.169574 | 103 | -3.17038 | 0.148873 | 1.4887305323e-01 |  |
| 1_339B - 3_331B | -0.13082 | 0.169574 | 103 | -0.77147 | 0.999998 | 9.9999808308e-01 |  |
| 1_339B - 4_188B | -1.31605 | 0.169574 | 103 | -7.76093 | 9.72E-10 | 9.7198860072e-10 | *** |
| 1_339B - 5_Obicolor | -1.96987 | 0.169574 | 103 | -11.6166 | 3.67E-14 | 3.6748382115e-14 | *** |
| 1_339B - 6_F1 | -0.52845 | 0.176498 | 103 | -2.99406 | 0.222834 | 2.2283391521e-01 |  |
| 1_339B - 7_F2 | -1.00229 | 0.169574 | 103 | -5.91064 | 6.54E-06 | 6.5376671095e-06 | *** |
| 1_339B - 8_F5 | -1.94531 | 0.176498 | 103 | -11.0217 | 8.43E-14 | 8.4265927569e-14 | *** |
| 1_339B - 9_F31 | -0.7193 | 0.169574 | 103 | -4.24183 | 0.005832 | 5.8317926546e-03 | ** |
| 1_339B - 91_F39 | -1.25236 | 0.176498 | 103 | -7.09558 | 2.55E-08 | 2.5483144150e-08 | *** |
| 1_339B - 92_F55 | -1.69575 | 0.176498 | 103 | -9.60775 | 2.46E-13 | 2.4613644456e-13 | *** |
| 1_339B - 93_F54 | -1.39774 | 0.169574 | 103 | -8.24263 | 8.75E-11 | 8.7498119861e-11 | *** |
| 1_339B - 94_F58 | -2.15486 | 0.169574 | 103 | -12.7075 | 0 | 0.0000000000e+00 | *** |
| 1_339B - 95_F83 | -1.7028 | 0.169574 | 103 | -10.0416 | 1.62E-13 | 1.6187051699e-13 | *** |
| 1_339B - 96_F94 | -0.41084 | 0.169574 | 103 | -2.4228 | 0.592696 | 5.9269562319e-01 |  |
| 1_339B - 97_F66 | -0.02888 | 0.169574 | 103 | -0.1703 | 1 | 1.0000000000e+00 |  |
| 1_339B - 98_F26B | -1.76467 | 0.169574 | 103 | -10.4065 | 1.41E-13 | 1.4110934643e-13 | *** |
| 1_339B - 99_Control | 2.181079 | 0.176498 | 103 | 12.3575 | 3.89E-15 | 3.8857805862e-15 | *** |
| 2_159B - 3_331B | 0.406793 | 0.169574 | 103 | 2.398907 | 0.610205 | 6.1020493082e-01 |  |
| 2_159B - 4_188B | -0.77844 | 0.169574 | 103 | -4.59055 | 0.00162 | 1.6202150155e-03 | ** |
| 2_159B - 5_Obicolor | -1.43226 | 0.169574 | 103 | -8.4462 | 3.14E-11 | 3.1445845927e-11 | *** |
| 2_159B - 6_F1 | 0.009168 | 0.176498 | 103 | 0.051941 | 1 | 1.0000000000e+00 |  |
| 2_159B - 7_F2 | -0.46468 | 0.169574 | 103 | -2.74026 | 0.367309 | 3.6730894139e-01 |  |
| 2_159B - 8_F5 | -1.40769 | 0.176498 | 103 | -7.97568 | 3.33E-10 | 3.3339331296e-10 | *** |
| 2_159B - 9_F31 | -0.18169 | 0.169574 | 103 | -1.07145 | 0.999798 | 9.9979815647e-01 |  |
| 2_159B - 91_F39 | -0.71474 | 0.176498 | 103 | -4.04958 | 0.011341 | 1.1341255888e-02 | * |
| 2_159B - 92_F55 | -1.15814 | 0.176498 | 103 | -6.56175 | 3.27E-07 | 3.2655293680e-07 | *** |
| 2_159B - 93_F54 | -0.86012 | 0.169574 | 103 | -5.07225 | 0.000241 | 2.4130934882e-04 | *** |
| 2_159B - 94_F58 | -1.61724 | 0.169574 | 103 | -9.53707 | 2.85E-13 | 2.8455016121e-13 | *** |
| 2_159B - 95_F83 | -1.16519 | 0.169574 | 103 | -6.87126 | 7.51E-08 | 7.5083177564e-08 | *** |
| 2_159B - 96_F94 | 0.12677 | 0.169574 | 103 | 0.747579 | 0.999999 | 9.9999880451e-01 |  |
| 2_159B - 97_F66 | 0.508735 | 0.169574 | 103 | 3.000075 | 0.21995 | 2.1994977786e-01 |  |
| 2_159B - 98_F26B | -1.22706 | 0.169574 | 103 | -7.23612 | 1.29E-08 | 1.2874352584e-08 | *** |
| 2_159B - 99_Control | 2.718693 | 0.176498 | 103 | 15.4035 | 0 | 0.0000000000e+00 | *** |
| 3_331B - 4_188B | -1.18523 | 0.169574 | 103 | -6.98946 | 4.26E-08 | 4.2551126733e-08 | *** |
| 3_331B - 5_Obicolor | -1.83905 | 0.169574 | 103 | -10.8451 | 1.06E-13 | 1.0558220964e-13 | *** |
| 3_331B - 6_F1 | -0.39762 | 0.176498 | 103 | -2.25285 | 0.713822 | 7.1382228780e-01 |  |
| 3_331B - 7_F2 | -0.87147 | 0.169574 | 103 | -5.13917 | 0.000183 | 1.8321780129e-04 | *** |
| 3_331B - 8_F5 | -1.81449 | 0.176498 | 103 | -10.2805 | 1.46E-13 | 1.4621637234e-13 | *** |
| 3_331B - 9_F31 | -0.58848 | 0.169574 | 103 | -3.47035 | 0.068175 | 6.8175388072e-02 | . |
| 3_331B - 91_F39 | -1.12154 | 0.176498 | 103 | -6.35438 | 8.61E-07 | 8.6110583453e-07 | *** |
| 3_331B - 92_F55 | -1.56493 | 0.176498 | 103 | -8.86654 | 3.87E-12 | 3.8654635048e-12 | *** |
| 3_331B - 93_F54 | -1.26691 | 0.169574 | 103 | -7.47115 | 4.07E-09 | 4.0727129447e-09 | *** |
| 3_331B - 94_F58 | -2.02403 | 0.169574 | 103 | -11.936 | 1.65E-14 | 1.6542323067e-14 | *** |
| 3_331B - 95_F83 | -1.57198 | 0.169574 | 103 | -9.27017 | 6.39E-13 | 6.3893335067e-13 | *** |
| 3_331B - 96_F94 | -0.28002 | 0.169574 | 103 | -1.65133 | 0.971156 | 9.7115644100e-01 |  |
| 3_331B - 97_F66 | 0.101943 | 0.169574 | 103 | 0.601168 | 1 | 9.9999995848e-01 |  |
| 3_331B - 98_F26B | -1.63385 | 0.169574 | 103 | -9.63502 | 2.35E-13 | 2.3459012510e-13 | *** |
| 3_331B - 99_Control | 2.3119 | 0.176498 | 103 | 13.09871 | 0 | 0.0000000000e+00 | *** |
| 4_188B - 5_Obicolor | -0.65382 | 0.169574 | 103 | -3.85565 | 0.021463 | 2.1463342206e-02 | * |
| 4_188B - 6_F1 | 0.787607 | 0.176498 | 103 | 4.462402 | 0.002621 | 2.6212616509e-03 | ** |
| 4_188B - 7_F2 | 0.313762 | 0.169574 | 103 | 1.850292 | 0.922846 | 9.2284580051e-01 |  |
| 4_188B - 8_F5 | -0.62925 | 0.176498 | 103 | -3.56522 | 0.052062 | 5.2061870316e-02 | . |
| 4_188B - 9_F31 | 0.596749 | 0.169574 | 103 | 3.519105 | 0.05943 | 5.9429680156e-02 | . |
| 4_188B - 91_F39 | 0.063695 | 0.176498 | 103 | 0.360879 | 1 | 9.9999999999e-01 |  |
| 4_188B - 92_F55 | -0.3797 | 0.176498 | 103 | -2.15129 | 0.779516 | 7.7951562690e-01 |  |
| 4_188B - 93_F54 | -0.08168 | 0.169574 | 103 | -0.48169 | 1 | 9.9999999879e-01 |  |
| 4_188B - 94_F58 | -0.8388 | 0.169574 | 103 | -4.94652 | 0.000402 | 4.0209784903e-04 | *** |
| 4_188B - 95_F83 | -0.38675 | 0.169574 | 103 | -2.28071 | 0.694718 | 6.9471789740e-01 |  |
| 4_188B - 96_F94 | 0.905209 | 0.169574 | 103 | 5.338132 | 7.97E-05 | 7.9668153308e-05 | *** |
| 4_188B - 97_F66 | 1.287174 | 0.169574 | 103 | 7.590628 | 2.26E-09 | 2.2598730665e-09 | *** |
| 4_188B - 98_F26B | -0.44862 | 0.169574 | 103 | -2.64556 | 0.43119 | 4.3118989304e-01 |  |
| 4_188B - 99_Control | 3.497132 | 0.176498 | 103 | 19.81396 | 0 | 0.0000000000e+00 | *** |
| 5_Obicolor - 6_F1 | 1.441425 | 0.176498 | 103 | 8.166791 | 1.28E-10 | 1.2802992000e-10 | *** |
| 5_Obicolor - 7_F2 | 0.96758 | 0.169574 | 103 | 5.705942 | 1.62E-05 | 1.6246280762e-05 | *** |
| 5_Obicolor - 8_F5 | 0.024564 | 0.176498 | 103 | 0.139172 | 1 | 1.0000000000e+00 |  |
| 5_Obicolor - 9_F31 | 1.250568 | 0.169574 | 103 | 7.374755 | 6.54E-09 | 6.5381272618e-09 | *** |
| 5_Obicolor - 91_F39 | 0.717513 | 0.176498 | 103 | 4.065269 | 0.010755 | 1.0754824772e-02 | * |
| 5_Obicolor - 92_F55 | 0.27412 | 0.176498 | 103 | 1.5531 | 0.984066 | 9.8406609641e-01 |  |
| 5_Obicolor - 93_F54 | 0.572136 | 0.169574 | 103 | 3.373957 | 0.088705 | 8.8704578401e-02 | . |
| 5_Obicolor - 94_F58 | -0.18498 | 0.169574 | 103 | -1.09087 | 0.999744 | 9.9974417202e-01 |  |
| 5_Obicolor - 95_F83 | 0.267069 | 0.169574 | 103 | 1.574942 | 0.981693 | 9.8169303373e-01 |  |
| 5_Obicolor - 96_F94 | 1.559028 | 0.169574 | 103 | 9.193782 | 8.56E-13 | 8.5564888508e-13 | *** |
| 5_Obicolor - 97_F66 | 1.940993 | 0.169574 | 103 | 11.44628 | 5.14E-14 | 5.1403326040e-14 | *** |
| 5_Obicolor - 98_F26B | 0.205199 | 0.169574 | 103 | 1.210086 | 0.999039 | 9.9903908788e-01 |  |
| 5_Obicolor - 99_Control | 4.150951 | 0.176498 | 103 | 23.51835 | 0 | 0.0000000000e+00 | *** |
| 6_F1 - 7_F2 | -0.47384 | 0.176498 | 103 | -2.6847 | 0.404262 | 4.0426244564e-01 |  |
| 6_F1 - 8_F5 | -1.41686 | 0.183161 | 103 | -7.73561 | 1.1E-09 | 1.1022416313e-09 | *** |
| 6_F1 - 9_F31 | -0.19086 | 0.176498 | 103 | -1.08136 | 0.999772 | 9.9977203221e-01 |  |
| 6_F1 - 91_F39 | -0.72391 | 0.183161 | 103 | -3.95233 | 0.015685 | 1.5684799526e-02 | * |
| 6_F1 - 92_F55 | -1.16731 | 0.183161 | 103 | -6.37311 | 7.89E-07 | 7.8930631109e-07 | *** |
| 6_F1 - 93_F54 | -0.86929 | 0.176498 | 103 | -4.9252 | 0.000438 | 4.3807288300e-04 | *** |
| 6_F1 - 94_F58 | -1.62641 | 0.176498 | 103 | -9.21486 | 7.88E-13 | 7.8836936979e-13 | *** |
| 6_F1 - 95_F83 | -1.17436 | 0.176498 | 103 | -6.65364 | 2.12E-07 | 2.1164434993e-07 | *** |
| 6_F1 - 96_F94 | 0.117602 | 0.176498 | 103 | 0.666309 | 1 | 9.9999979359e-01 |  |
| 6_F1 - 97_F66 | 0.499568 | 0.176498 | 103 | 2.830437 | 0.311084 | 3.1108355178e-01 |  |
| 6_F1 - 98_F26B | -1.23623 | 0.176498 | 103 | -7.00418 | 3.96E-08 | 3.9636654736e-08 | *** |
| 6_F1 - 99_Control | 2.709525 | 0.183161 | 103 | 14.79314 | 0 | 0.0000000000e+00 | *** |
| 7_F2 - 8_F5 | -0.94302 | 0.176498 | 103 | -5.34292 | 7.81E-05 | 7.8067764837e-05 | *** |
| 7_F2 - 9_F31 | 0.282988 | 0.169574 | 103 | 1.668813 | 0.968199 | 9.6819887914e-01 |  |
| 7_F2 - 91_F39 | -0.25007 | 0.176498 | 103 | -1.41682 | 0.993925 | 9.9392530962e-01 |  |
| 7_F2 - 92_F55 | -0.69346 | 0.176498 | 103 | -3.92899 | 0.016932 | 1.6931950838e-02 | * |
| 7_F2 - 93_F54 | -0.39544 | 0.169574 | 103 | -2.33198 | 0.658622 | 6.5862182315e-01 |  |
| 7_F2 - 94_F58 | -1.15256 | 0.169574 | 103 | -6.79681 | 1.07E-07 | 1.0718051102e-07 | *** |
| 7_F2 - 95_F83 | -0.70051 | 0.169574 | 103 | -4.131 | 0.00859 | 8.5897460704e-03 | ** |
| 7_F2 - 96_F94 | 0.591447 | 0.169574 | 103 | 3.48784 | 0.06492 | 6.4920199215e-02 | . |
| 7_F2 - 97_F66 | 0.973413 | 0.169574 | 103 | 5.740336 | 1.4E-05 | 1.3958827199e-05 | *** |
| 7_F2 - 98_F26B | -0.76238 | 0.169574 | 103 | -4.49586 | 0.002314 | 2.3144866730e-03 | ** |
| 7_F2 - 99_Control | 3.18337 | 0.176498 | 103 | 18.03626 | 0 | 0.0000000000e+00 | *** |
| 8_F5 - 9_F31 | 1.226004 | 0.176498 | 103 | 6.946264 | 5.24E-08 | 5.2385757843e-08 | *** |
| 8_F5 - 91_F39 | 0.69295 | 0.183161 | 103 | 3.783282 | 0.026984 | 2.6983951751e-02 | * |
| 8_F5 - 92_F55 | 0.249556 | 0.183161 | 103 | 1.362495 | 0.996073 | 9.9607272767e-01 |  |
| 8_F5 - 93_F54 | 0.547572 | 0.176498 | 103 | 3.102422 | 0.174814 | 1.7481365145e-01 |  |
| 8_F5 - 94_F58 | -0.20955 | 0.176498 | 103 | -1.18725 | 0.999242 | 9.9924191414e-01 |  |
| 8_F5 - 95_F83 | 0.242506 | 0.176498 | 103 | 1.373984 | 0.995682 | 9.9568177843e-01 |  |
| 8_F5 - 96_F94 | 1.534464 | 0.176498 | 103 | 8.693928 | 9.09E-12 | 9.0853990997e-12 | *** |
| 8_F5 - 97_F66 | 1.916429 | 0.176498 | 103 | 10.85806 | 1.04E-13 | 1.0369483050e-13 | *** |
| 8_F5 - 98_F26B | 0.180636 | 0.176498 | 103 | 1.023441 | 0.999891 | 9.9989080976e-01 |  |
| 8_F5 - 99_Control | 4.126387 | 0.183161 | 103 | 22.52874 | 0 | 0.0000000000e+00 | *** |
| 9_F31 - 91_F39 | -0.53305 | 0.176498 | 103 | -3.02017 | 0.210502 | 2.1050224588e-01 |  |
| 9_F31 - 92_F55 | -0.97645 | 0.176498 | 103 | -5.53234 | 3.47E-05 | 3.4676172286e-05 | *** |
| 9_F31 - 93_F54 | -0.67843 | 0.169574 | 103 | -4.0008 | 0.013359 | 1.3358540551e-02 | * |
| 9_F31 - 94_F58 | -1.43555 | 0.169574 | 103 | -8.46562 | 2.85E-11 | 2.8518409856e-11 | *** |
| 9_F31 - 95_F83 | -0.9835 | 0.169574 | 103 | -5.79981 | 1.07E-05 | 1.0724514720e-05 | *** |
| 9_F31 - 96_F94 | 0.30846 | 0.169574 | 103 | 1.819027 | 0.932742 | 9.3274242038e-01 |  |
| 9_F31 - 97_F66 | 0.690425 | 0.169574 | 103 | 4.071523 | 0.010529 | 1.0528922104e-02 | * |
| 9_F31 - 98_F26B | -1.04537 | 0.169574 | 103 | -6.16467 | 2.07E-06 | 2.0658179687e-06 | *** |
| 9_F31 - 99_Control | 2.900383 | 0.176498 | 103 | 16.43292 | 0 | 0.0000000000e+00 | *** |
| 91_F39 - 92_F55 | -0.44339 | 0.183161 | 103 | -2.42079 | 0.594175 | 5.9417468957e-01 |  |
| 91_F39 - 93_F54 | -0.14538 | 0.176498 | 103 | -0.82368 | 0.999995 | 9.9999494271e-01 |  |
| 91_F39 - 94_F58 | -0.9025 | 0.176498 | 103 | -5.11334 | 0.000204 | 2.0382203750e-04 | *** |
| 91_F39 - 95_F83 | -0.45044 | 0.176498 | 103 | -2.55211 | 0.497813 | 4.9781331024e-01 |  |
| 91_F39 - 96_F94 | 0.841514 | 0.176498 | 103 | 4.767831 | 0.000818 | 8.1755688504e-04 | *** |
| 91_F39 - 97_F66 | 1.223479 | 0.176498 | 103 | 6.93196 | 5.61E-08 | 5.6114242253e-08 | *** |
| 91_F39 - 98_F26B | -0.51231 | 0.176498 | 103 | -2.90266 | 0.269836 | 2.6983587372e-01 |  |
| 91_F39 - 99_Control | 3.433437 | 0.183161 | 103 | 18.74546 | 0 | 0.0000000000e+00 | *** |
| 92_F55 - 93_F54 | 0.298016 | 0.176498 | 103 | 1.688494 | 0.964603 | 9.6460273559e-01 |  |
| 92_F55 - 94_F58 | -0.4591 | 0.176498 | 103 | -2.60117 | 0.462488 | 4.6248842368e-01 |  |
| 92_F55 - 95_F83 | -0.00705 | 0.176498 | 103 | -0.03994 | 1 | 1.0000000000e+00 |  |
| 92_F55 - 96_F94 | 1.284908 | 0.176498 | 103 | 7.28 | 1.04E-08 | 1.0393500172e-08 | *** |
| 92_F55 - 97_F66 | 1.666873 | 0.176498 | 103 | 9.444129 | 3.53E-13 | 3.5293989953e-13 | *** |
| 92_F55 - 98_F26B | -0.06892 | 0.176498 | 103 | -0.39049 | 1 | 9.9999999996e-01 |  |
| 92_F55 - 99_Control | 3.876831 | 0.183161 | 103 | 21.16625 | 0 | 0.0000000000e+00 | *** |
| 93_F54 - 94_F58 | -0.75712 | 0.169574 | 103 | -4.46483 | 0.002598 | 2.5977919177e-03 | ** |
| 93_F54 - 95_F83 | -0.30507 | 0.169574 | 103 | -1.79902 | 0.938596 | 9.3859619846e-01 |  |
| 93_F54 - 96_F94 | 0.986892 | 0.169574 | 103 | 5.819825 | 9.81E-06 | 9.8111950783e-06 | *** |
| 93_F54 - 97_F66 | 1.368857 | 0.169574 | 103 | 8.072321 | 2.06E-10 | 2.0557211489e-10 | *** |
| 93_F54 - 98_F26B | -0.36694 | 0.169574 | 103 | -2.16387 | 0.771764 | 7.7176369959e-01 |  |
| 93_F54 - 99_Control | 3.578815 | 0.176498 | 103 | 20.27676 | 0 | 0.0000000000e+00 | *** |
| 94_F58 - 95_F83 | 0.452053 | 0.169574 | 103 | 2.665811 | 0.417173 | 4.1717344831e-01 |  |
| 94_F58 - 96_F94 | 1.744011 | 0.169574 | 103 | 10.28465 | 1.45E-13 | 1.4488410471e-13 | *** |
| 94_F58 - 97_F66 | 2.125976 | 0.169574 | 103 | 12.53715 | 0 | 0.0000000000e+00 | *** |
| 94_F58 - 98_F26B | 0.390182 | 0.169574 | 103 | 2.300955 | 0.680596 | 6.8059597351e-01 |  |
| 94_F58 - 99_Control | 4.335934 | 0.176498 | 103 | 24.56643 | 0 | 0.0000000000e+00 | *** |
| 95_F83 - 96_F94 | 1.291958 | 0.169574 | 103 | 7.61884 | 1.97E-09 | 1.9657461214e-09 | *** |
| 95_F83 - 97_F66 | 1.673923 | 0.169574 | 103 | 9.871336 | 1.76E-13 | 1.7641443861e-13 | *** |
| 95_F83 - 98_F26B | -0.06187 | 0.169574 | 103 | -0.36486 | 1 | 9.9999999999e-01 |  |
| 95_F83 - 99_Control | 3.883881 | 0.176498 | 103 | 22.0052 | 0 | 0.0000000000e+00 | *** |
| 96_F94 - 97_F66 | 0.381965 | 0.169574 | 103 | 2.252497 | 0.714064 | 7.1406449926e-01 |  |
| 96_F94 - 98_F26B | -1.35383 | 0.169574 | 103 | -7.9837 | 3.2E-10 | 3.2029656705e-10 | *** |
| 96_F94 - 99_Control | 2.591923 | 0.176498 | 103 | 14.68525 | 0 | 0.0000000000e+00 | *** |
| 97_F66 - 98_F26B | -1.73579 | 0.169574 | 103 | -10.2362 | 1.44E-13 | 1.4410694860e-13 | *** |
| 97_F66 - 99_Control | 2.209958 | 0.176498 | 103 | 12.52113 | 0 | 0.0000000000e+00 | *** |
| 98_F26B - 99_Control | 3.945751 | 0.176498 | 103 | 22.35574 | 0 | 0.0000000000e+00 | *** |

| Sample | emmean | SE | df | lower.CL | upper.CL | .group |
| --- | --- | --- | --- | --- | --- | --- |
| 99_Control | 1.272885 | 0.129514 | 103 | 0.876922 | 1.668848 | a |
| 1_339B | 3.453964 | 0.119907 | 103 | 3.087373 | 3.820555 | b |
| 97_F66 | 3.482843 | 0.119907 | 103 | 3.116253 | 3.849434 | b |
| 3_331B | 3.584786 | 0.119907 | 103 | 3.218195 | 3.951376 | bc |
| 96_F94 | 3.864808 | 0.119907 | 103 | 3.498218 | 4.231399 | bcd |
| 6_F1 | 3.982411 | 0.129514 | 103 | 3.586448 | 4.378374 | bcd |
| 2_159B | 3.991578 | 0.119907 | 103 | 3.624988 | 4.358169 | bcd |
| 9_F31 | 4.173268 | 0.119907 | 103 | 3.806678 | 4.539859 | cde |
| 7_F2 | 4.456256 | 0.119907 | 103 | 4.089665 | 4.822846 | def |
| 91_F39 | 4.706323 | 0.129514 | 103 | 4.31036 | 5.102286 | efg |
| 4_188B | 4.770017 | 0.119907 | 103 | 4.403427 | 5.136608 | efgh |
| 93_F54 | 4.8517 | 0.119907 | 103 | 4.485109 | 5.218291 | fghi |
| 92_F55 | 5.149716 | 0.129514 | 103 | 4.753753 | 5.54568 | ghij |
| 95_F83 | 5.156767 | 0.119907 | 103 | 4.790176 | 5.523357 | ghij |
| 98_F26B | 5.218637 | 0.119907 | 103 | 4.852046 | 5.585227 | ghij |
| 8_F5 | 5.399272 | 0.129514 | 103 | 5.003309 | 5.795235 | hij |
| 5_Obicolor | 5.423836 | 0.119907 | 103 | 5.057245 | 5.790427 | ij |
| 94_F58 | 5.608819 | 0.119907 | 103 | 5.242229 | 5.97541 | j |

**Total B vitamins**

| Modell |
| --- |
| Normal_log |

| Resid. Df | Resid. Dev | Df | Deviance | Pr(>Chi) | p.value_formatted | Significance |
| --- | --- | --- | --- | --- | --- | --- |
| 120 | 67.68448 |  |  |  | NA |  |
| 103 | 17.18623 | 17 | 50.49825 | 3.2E-54 | 3.2029866799e-54 | *** |

| contrast | estimate | SE | df | t.ratio | p.value | p.value_formatted | Significance |
| --- | --- | --- | --- | --- | --- | --- | --- |
| 1_339B - 2_159B | 0.18961 | 0.218342 | 103 | 0.868406 | 0.999989 | 9.9998907621e-01 |  |
| 1_339B - 3_331B | 0.247766 | 0.218342 | 103 | 1.13476 | 0.999573 | 9.9957285295e-01 |  |
| 1_339B - 4_188B | -0.04431 | 0.218342 | 103 | -0.20292 | 1 | 1.0000000000e+00 |  |
| 1_339B - 5_O.bicolor | 0.470217 | 0.218342 | 103 | 2.153578 | 0.778115 | 7.7811457612e-01 |  |
| 1_339B - 6_F1 | 0.266838 | 0.227258 | 103 | 1.174164 | 0.99934 | 9.9934038948e-01 |  |
| 1_339B - 7_F2 | -0.1944 | 0.218342 | 103 | -0.89035 | 0.999984 | 9.9998435877e-01 |  |
| 1_339B - 8_F5 | 0.078497 | 0.227258 | 103 | 0.345411 | 1 | 9.9999999999e-01 |  |
| 1_339B - 9_F31 | 0.617606 | 0.218342 | 103 | 2.828613 | 0.312171 | 3.1217062818e-01 |  |
| 1_339B - 91_F39 | -0.2194 | 0.227258 | 103 | -0.96541 | 0.999951 | 9.9995098803e-01 |  |
| 1_339B - 92_F55 | -0.96681 | 0.227258 | 103 | -4.25426 | 0.00558 | 5.5803334094e-03 | ** |
| 1_339B - 93_F54 | 0.345597 | 0.218342 | 103 | 1.582824 | 0.980772 | 9.8077164230e-01 |  |
| 1_339B - 94_F58 | 0.356886 | 0.218342 | 103 | 1.634525 | 0.973797 | 9.7379710814e-01 |  |
| 1_339B - 95_F83 | -0.00973 | 0.218342 | 103 | -0.04455 | 1 | 1.0000000000e+00 |  |
| 1_339B - 96_F94 | 0.922503 | 0.218342 | 103 | 4.225033 | 0.006188 | 6.1882625528e-03 | ** |
| 1_339B - 97_F66 | 0.292104 | 0.218342 | 103 | 1.337826 | 0.996813 | 9.9681270700e-01 |  |
| 1_339B - 98_F26B | -0.48428 | 0.218342 | 103 | -2.21801 | 0.73712 | 7.3711968129e-01 |  |
| 1_339B - 99_Control | 2.442093 | 0.227258 | 103 | 10.74592 | 1.12E-13 | 1.1224354779e-13 | *** |
| 2_159B - 3_331B | 0.058156 | 0.218342 | 103 | 0.266354 | 1 | 1.0000000000e+00 |  |
| 2_159B - 4_188B | -0.23392 | 0.218342 | 103 | -1.07132 | 0.999798 | 9.9979846686e-01 |  |
| 2_159B - 5_O.bicolor | 0.280607 | 0.218342 | 103 | 1.285172 | 0.998007 | 9.9800672296e-01 |  |
| 2_159B - 6_F1 | 0.077228 | 0.227258 | 103 | 0.339826 | 1 | 1.0000000000e+00 |  |
| 2_159B - 7_F2 | -0.38401 | 0.218342 | 103 | -1.75876 | 0.949272 | 9.4927242418e-01 |  |
| 2_159B - 8_F5 | -0.11111 | 0.227258 | 103 | -0.48893 | 1 | 9.9999999846e-01 |  |
| 2_159B - 9_F31 | 0.427996 | 0.218342 | 103 | 1.960207 | 0.880526 | 8.8052589749e-01 |  |
| 2_159B - 91_F39 | -0.40901 | 0.227258 | 103 | -1.79975 | 0.938387 | 9.3838748847e-01 |  |
| 2_159B - 92_F55 | -1.15642 | 0.227258 | 103 | -5.0886 | 0.000226 | 2.2565429184e-04 | *** |
| 2_159B - 93_F54 | 0.155988 | 0.218342 | 103 | 0.714418 | 0.999999 | 9.9999939870e-01 |  |
| 2_159B - 94_F58 | 0.167276 | 0.218342 | 103 | 0.766119 | 0.999998 | 9.9999827276e-01 |  |
| 2_159B - 95_F83 | -0.19934 | 0.218342 | 103 | -0.91296 | 0.999978 | 9.9997763906e-01 |  |
| 2_159B - 96_F94 | 0.732893 | 0.218342 | 103 | 3.356628 | 0.092892 | 9.2892256640e-02 | . |
| 2_159B - 97_F66 | 0.102494 | 0.218342 | 103 | 0.46942 | 1 | 9.9999999920e-01 |  |
| 2_159B - 98_F26B | -0.67389 | 0.218342 | 103 | -3.08641 | 0.181387 | 1.8138663051e-01 |  |
| 2_159B - 99_Control | 2.252483 | 0.227258 | 103 | 9.911578 | 1.69E-13 | 1.6864287744e-13 | *** |
| 3_331B - 4_188B | -0.29207 | 0.218342 | 103 | -1.33768 | 0.996817 | 9.9681677424e-01 |  |
| 3_331B - 5_O.bicolor | 0.222451 | 0.218342 | 103 | 1.018818 | 0.999897 | 9.9989731240e-01 |  |
| 3_331B - 6_F1 | 0.019072 | 0.227258 | 103 | 0.083922 | 1 | 1.0000000000e+00 |  |
| 3_331B - 7_F2 | -0.44217 | 0.218342 | 103 | -2.02511 | 0.849977 | 8.4997665913e-01 |  |
| 3_331B - 8_F5 | -0.16927 | 0.227258 | 103 | -0.74483 | 0.999999 | 9.9999886906e-01 |  |
| 3_331B - 9_F31 | 0.36984 | 0.218342 | 103 | 1.693853 | 0.963573 | 9.6357276315e-01 |  |
| 3_331B - 91_F39 | -0.46716 | 0.227258 | 103 | -2.05566 | 0.834213 | 8.3421346378e-01 |  |
| 3_331B - 92_F55 | -1.21458 | 0.227258 | 103 | -5.3445 | 7.75E-05 | 7.7545151998e-05 | *** |
| 3_331B - 93_F54 | 0.097831 | 0.218342 | 103 | 0.448064 | 1 | 9.9999999963e-01 |  |
| 3_331B - 94_F58 | 0.10912 | 0.218342 | 103 | 0.499765 | 1 | 9.9999999781e-01 |  |
| 3_331B - 95_F83 | -0.25749 | 0.218342 | 103 | -1.17931 | 0.999303 | 9.9930307249e-01 |  |
| 3_331B - 96_F94 | 0.674737 | 0.218342 | 103 | 3.090274 | 0.179785 | 1.7978478439e-01 |  |
| 3_331B - 97_F66 | 0.044338 | 0.218342 | 103 | 0.203066 | 1 | 1.0000000000e+00 |  |
| 3_331B - 98_F26B | -0.73205 | 0.218342 | 103 | -3.35277 | 0.093847 | 9.3847310266e-02 | . |
| 3_331B - 99_Control | 2.194327 | 0.227258 | 103 | 9.655674 | 2.23E-13 | 2.2304380565e-13 | *** |
| 4_188B - 5_O.bicolor | 0.514522 | 0.218342 | 103 | 2.356496 | 0.641026 | 6.4102560911e-01 |  |
| 4_188B - 6_F1 | 0.311143 | 0.227258 | 103 | 1.369121 | 0.995851 | 9.9585106057e-01 |  |
| 4_188B - 7_F2 | -0.1501 | 0.218342 | 103 | -0.68744 | 1 | 9.9999966620e-01 |  |
| 4_188B - 8_F5 | 0.122803 | 0.227258 | 103 | 0.540369 | 1 | 9.9999999234e-01 |  |
| 4_188B - 9_F31 | 0.661911 | 0.218342 | 103 | 3.031531 | 0.205286 | 2.0528631075e-01 |  |
| 4_188B - 91_F39 | -0.17509 | 0.227258 | 103 | -0.77046 | 0.999998 | 9.9999812049e-01 |  |
| 4_188B - 92_F55 | -0.92251 | 0.227258 | 103 | -4.05931 | 0.010974 | 1.0974353961e-02 | * |
| 4_188B - 93_F54 | 0.389903 | 0.218342 | 103 | 1.785742 | 0.942277 | 9.4227664194e-01 |  |
| 4_188B - 94_F58 | 0.401191 | 0.218342 | 103 | 1.837443 | 0.927025 | 9.2702522822e-01 |  |
| 4_188B - 95_F83 | 0.034578 | 0.218342 | 103 | 0.158368 | 1 | 1.0000000000e+00 |  |
| 4_188B - 96_F94 | 0.966809 | 0.218342 | 103 | 4.427951 | 0.002977 | 2.9771882659e-03 | ** |
| 4_188B - 97_F66 | 0.336409 | 0.218342 | 103 | 1.540744 | 0.985296 | 9.8529643020e-01 |  |
| 4_188B - 98_F26B | -0.43998 | 0.218342 | 103 | -2.01509 | 0.854961 | 8.5496072653e-01 |  |
| 4_188B - 99_Control | 2.486398 | 0.227258 | 103 | 10.94087 | 8.95E-14 | 8.9483975785e-14 | *** |
| 5_O.bicolor - 6_F1 | -0.20338 | 0.227258 | 103 | -0.89493 | 0.999983 | 9.9998316940e-01 |  |
| 5_O.bicolor - 7_F2 | -0.66462 | 0.218342 | 103 | -3.04393 | 0.199699 | 1.9969908732e-01 |  |
| 5_O.bicolor - 8_F5 | -0.39172 | 0.227258 | 103 | -1.72368 | 0.95743 | 9.5743004591e-01 |  |
| 5_O.bicolor - 9_F31 | 0.147389 | 0.218342 | 103 | 0.675035 | 1 | 9.9999974771e-01 |  |
| 5_O.bicolor - 91_F39 | -0.68961 | 0.227258 | 103 | -3.0345 | 0.203937 | 2.0393677032e-01 |  |
| 5_O.bicolor - 92_F55 | -1.43703 | 0.227258 | 103 | -6.32335 | 9.94E-07 | 9.9438637824e-07 | *** |
| 5_O.bicolor - 93_F54 | -0.12462 | 0.218342 | 103 | -0.57075 | 1 | 9.9999998171e-01 |  |
| 5_O.bicolor - 94_F58 | -0.11333 | 0.218342 | 103 | -0.51905 | 1 | 9.9999999597e-01 |  |
| 5_O.bicolor - 95_F83 | -0.47994 | 0.218342 | 103 | -2.19813 | 0.750074 | 7.5007377889e-01 |  |
| 5_O.bicolor - 96_F94 | 0.452286 | 0.218342 | 103 | 2.071456 | 0.825722 | 8.2572197331e-01 |  |
| 5_O.bicolor - 97_F66 | -0.17811 | 0.218342 | 103 | -0.81575 | 0.999996 | 9.9999561263e-01 |  |
| 5_O.bicolor - 98_F26B | -0.9545 | 0.218342 | 103 | -4.37158 | 0.00366 | 3.6597853118e-03 | ** |
| 5_O.bicolor - 99_Control | 1.971876 | 0.227258 | 103 | 8.676825 | 9.9E-12 | 9.8963059969e-12 | *** |
| 6_F1 - 7_F2 | -0.46124 | 0.227258 | 103 | -2.02959 | 0.847722 | 8.4772182946e-01 |  |
| 6_F1 - 8_F5 | -0.18834 | 0.235837 | 103 | -0.79861 | 0.999997 | 9.9999679392e-01 |  |
| 6_F1 - 9_F31 | 0.350768 | 0.227258 | 103 | 1.54348 | 0.985031 | 9.8503071965e-01 |  |
| 6_F1 - 91_F39 | -0.48624 | 0.235837 | 103 | -2.06175 | 0.830966 | 8.3096581355e-01 |  |
| 6_F1 - 92_F55 | -1.23365 | 0.235837 | 103 | -5.23096 | 0.000125 | 1.2508516801e-04 | *** |
| 6_F1 - 93_F54 | 0.078759 | 0.227258 | 103 | 0.346564 | 1 | 9.9999999999e-01 |  |
| 6_F1 - 94_F58 | 0.090048 | 0.227258 | 103 | 0.396237 | 1 | 9.9999999995e-01 |  |
| 6_F1 - 95_F83 | -0.27656 | 0.227258 | 103 | -1.21697 | 0.998969 | 9.9896943183e-01 |  |
| 6_F1 - 96_F94 | 0.655665 | 0.227258 | 103 | 2.885117 | 0.279527 | 2.7952669821e-01 |  |
| 6_F1 - 97_F66 | 0.025266 | 0.227258 | 103 | 0.111178 | 1 | 1.0000000000e+00 |  |
| 6_F1 - 98_F26B | -0.75112 | 0.227258 | 103 | -3.30515 | 0.106298 | 1.0629821306e-01 |  |
| 6_F1 - 99_Control | 2.175255 | 0.235837 | 103 | 9.223571 | 7.61E-13 | 7.6116890568e-13 | *** |
| 7_F2 - 8_F5 | 0.272899 | 0.227258 | 103 | 1.200837 | 0.999126 | 9.9912627710e-01 |  |
| 7_F2 - 9_F31 | 0.812008 | 0.218342 | 103 | 3.718968 | 0.032926 | 3.2925893842e-02 | * |
| 7_F2 - 91_F39 | -0.025 | 0.227258 | 103 | -0.10999 | 1 | 1.0000000000e+00 |  |
| 7_F2 - 92_F55 | -0.77241 | 0.227258 | 103 | -3.39884 | 0.082967 | 8.2966936407e-02 | . |
| 7_F2 - 93_F54 | 0.539999 | 0.218342 | 103 | 2.473179 | 0.55563 | 5.5563028578e-01 |  |
| 7_F2 - 94_F58 | 0.551288 | 0.218342 | 103 | 2.52488 | 0.517666 | 5.1766592125e-01 |  |
| 7_F2 - 95_F83 | 0.184675 | 0.218342 | 103 | 0.845805 | 0.999993 | 9.9999254783e-01 |  |
| 7_F2 - 96_F94 | 1.116905 | 0.218342 | 103 | 5.115388 | 0.000202 | 2.0211088917e-04 | *** |
| 7_F2 - 97_F66 | 0.486506 | 0.218342 | 103 | 2.228181 | 0.730392 | 7.3039180222e-01 |  |
| 7_F2 - 98_F26B | -0.28988 | 0.218342 | 103 | -1.32765 | 0.997082 | 9.9708160808e-01 |  |
| 7_F2 - 99_Control | 2.636495 | 0.227258 | 103 | 11.60134 | 3.92E-14 | 3.9190872769e-14 | *** |
| 8_F5 - 9_F31 | 0.539108 | 0.227258 | 103 | 2.372232 | 0.629637 | 6.2963742506e-01 |  |
| 8_F5 - 91_F39 | -0.2979 | 0.235837 | 103 | -1.26314 | 0.998379 | 9.9837854645e-01 |  |
| 8_F5 - 92_F55 | -1.04531 | 0.235837 | 103 | -4.43236 | 0.002929 | 2.9292485297e-03 | ** |
| 8_F5 - 93_F54 | 0.2671 | 0.227258 | 103 | 1.175317 | 0.999332 | 9.9933218359e-01 |  |
| 8_F5 - 94_F58 | 0.278388 | 0.227258 | 103 | 1.224989 | 0.998883 | 9.9888270918e-01 |  |
| 8_F5 - 95_F83 | -0.08822 | 0.227258 | 103 | -0.38821 | 1 | 9.9999999996e-01 |  |
| 8_F5 - 96_F94 | 0.844006 | 0.227258 | 103 | 3.71387 | 0.033443 | 3.3443424852e-02 | * |
| 8_F5 - 97_F66 | 0.213606 | 0.227258 | 103 | 0.93993 | 0.999966 | 9.9996626898e-01 |  |
| 8_F5 - 98_F26B | -0.56278 | 0.227258 | 103 | -2.4764 | 0.553257 | 5.5325718745e-01 |  |
| 8_F5 - 99_Control | 2.363595 | 0.235837 | 103 | 10.02218 | 1.64E-13 | 1.6442402995e-13 | *** |
| 9_F31 - 91_F39 | -0.837 | 0.227258 | 103 | -3.68306 | 0.036728 | 3.6727682504e-02 | * |
| 9_F31 - 92_F55 | -1.58442 | 0.227258 | 103 | -6.97191 | 4.63E-08 | 4.6305225987e-08 | *** |
| 9_F31 - 93_F54 | -0.27201 | 0.218342 | 103 | -1.24579 | 0.998628 | 9.9862793534e-01 |  |
| 9_F31 - 94_F58 | -0.26072 | 0.218342 | 103 | -1.19409 | 0.999185 | 9.9918547419e-01 |  |
| 9_F31 - 95_F83 | -0.62733 | 0.218342 | 103 | -2.87316 | 0.286253 | 2.8625340180e-01 |  |
| 9_F31 - 96_F94 | 0.304897 | 0.218342 | 103 | 1.39642 | 0.994824 | 9.9482404548e-01 |  |
| 9_F31 - 97_F66 | -0.3255 | 0.218342 | 103 | -1.49079 | 0.989522 | 9.8952211639e-01 |  |
| 9_F31 - 98_F26B | -1.10189 | 0.218342 | 103 | -5.04662 | 0.000268 | 2.6797322683e-04 | *** |
| 9_F31 - 99_Control | 1.824487 | 0.227258 | 103 | 8.028272 | 2.56E-10 | 2.5629443012e-10 | *** |
| 91_F39 - 92_F55 | -0.74742 | 0.235837 | 103 | -3.16921 | 0.149292 | 1.4929163268e-01 |  |
| 91_F39 - 93_F54 | 0.564995 | 0.227258 | 103 | 2.486142 | 0.546091 | 5.4609092390e-01 |  |
| 91_F39 - 94_F58 | 0.576283 | 0.227258 | 103 | 2.535814 | 0.509678 | 5.0967828608e-01 |  |
| 91_F39 - 95_F83 | 0.209671 | 0.227258 | 103 | 0.922612 | 0.999974 | 9.9997404477e-01 |  |
| 91_F39 - 96_F94 | 1.141901 | 0.227258 | 103 | 5.024695 | 0.000293 | 2.9302579620e-04 | *** |
| 91_F39 - 97_F66 | 0.511502 | 0.227258 | 103 | 2.250755 | 0.715245 | 7.1524479227e-01 |  |
| 91_F39 - 98_F26B | -0.26489 | 0.227258 | 103 | -1.16558 | 0.999399 | 9.9939878247e-01 |  |
| 91_F39 - 99_Control | 2.66149 | 0.235837 | 103 | 11.28532 | 6.24E-14 | 6.2394533984e-14 | *** |
| 92_F55 - 93_F54 | 1.312411 | 0.227258 | 103 | 5.774991 | 1.2E-05 | 1.1973704861e-05 | *** |
| 92_F55 - 94_F58 | 1.3237 | 0.227258 | 103 | 5.824663 | 9.6E-06 | 9.6020599127e-06 | *** |
| 92_F55 - 95_F83 | 0.957087 | 0.227258 | 103 | 4.211461 | 0.006491 | 6.4911242296e-03 | ** |
| 92_F55 - 96_F94 | 1.889317 | 0.227258 | 103 | 8.313544 | 6.13E-11 | 6.1260885253e-11 | *** |
| 92_F55 - 97_F66 | 1.258918 | 0.227258 | 103 | 5.539604 | 3.36E-05 | 3.3601714293e-05 | *** |
| 92_F55 - 98_F26B | 0.48253 | 0.227258 | 103 | 2.123272 | 0.796335 | 7.9633474282e-01 |  |
| 92_F55 - 99_Control | 3.408907 | 0.235837 | 103 | 14.45453 | 0 | 0.0000000000e+00 | *** |
| 93_F54 - 94_F58 | 0.011288 | 0.218342 | 103 | 0.051701 | 1 | 1.0000000000e+00 |  |
| 93_F54 - 95_F83 | -0.35532 | 0.218342 | 103 | -1.62737 | 0.974863 | 9.7486305184e-01 |  |
| 93_F54 - 96_F94 | 0.576906 | 0.218342 | 103 | 2.64221 | 0.433529 | 4.3352922018e-01 |  |
| 93_F54 - 97_F66 | -0.05349 | 0.218342 | 103 | -0.245 | 1 | 1.0000000000e+00 |  |
| 93_F54 - 98_F26B | -0.82988 | 0.218342 | 103 | -3.80083 | 0.025539 | 2.5539193461e-02 | * |
| 93_F54 - 99_Control | 2.096496 | 0.227258 | 103 | 9.225188 | 7.55E-13 | 7.5528472365e-13 | *** |
| 94_F58 - 95_F83 | -0.36661 | 0.218342 | 103 | -1.67907 | 0.96636 | 9.6635993098e-01 |  |
| 94_F58 - 96_F94 | 0.565617 | 0.218342 | 103 | 2.590509 | 0.470109 | 4.7010934356e-01 |  |
| 94_F58 - 97_F66 | -0.06478 | 0.218342 | 103 | -0.2967 | 1 | 1.0000000000e+00 |  |
| 94_F58 - 98_F26B | -0.84117 | 0.218342 | 103 | -3.85253 | 0.021678 | 2.1678472796e-02 | * |
| 94_F58 - 99_Control | 2.085207 | 0.227258 | 103 | 9.175515 | 9.23E-13 | 9.2337248958e-13 | *** |
| 95_F83 - 96_F94 | 0.93223 | 0.218342 | 103 | 4.269583 | 0.005284 | 5.2844846129e-03 | ** |
| 95_F83 - 97_F66 | 0.301831 | 0.218342 | 103 | 1.382376 | 0.995376 | 9.9537605795e-01 |  |
| 95_F83 - 98_F26B | -0.47456 | 0.218342 | 103 | -2.17346 | 0.765779 | 7.6577943548e-01 |  |
| 95_F83 - 99_Control | 2.45182 | 0.227258 | 103 | 10.78872 | 1.03E-13 | 1.0302869669e-13 | *** |
| 96_F94 - 97_F66 | -0.6304 | 0.218342 | 103 | -2.88721 | 0.278361 | 2.7836059877e-01 |  |
| 96_F94 - 98_F26B | -1.40679 | 0.218342 | 103 | -6.44304 | 5.7E-07 | 5.6978102136e-07 | *** |
| 96_F94 - 99_Control | 1.51959 | 0.227258 | 103 | 6.686635 | 1.81E-07 | 1.8101515031e-07 | *** |
| 97_F66 - 98_F26B | -0.77639 | 0.218342 | 103 | -3.55583 | 0.053494 | 5.3493847380e-02 | . |
| 97_F66 - 99_Control | 2.149989 | 0.227258 | 103 | 9.460574 | 3.38E-13 | 3.3761882179e-13 | *** |
| 98_F26B - 99_Control | 2.926377 | 0.227258 | 103 | 12.87691 | 0 | 0.0000000000e+00 | *** |

| Sample | emmean | SE | df | lower.CL | upper.CL | .group |
| --- | --- | --- | --- | --- | --- | --- |
| 99_Control | 2.269447 | 0.166762 | 103 | 1.759608 | 2.779286 | a |
| 96_F94 | 3.789037 | 0.154391 | 103 | 3.317018 | 4.261055 | b |
| 9_F31 | 4.093934 | 0.154391 | 103 | 3.621915 | 4.565953 | bc |
| 5_O.bicolor | 4.241323 | 0.154391 | 103 | 3.769304 | 4.713342 | bcd |
| 94_F58 | 4.354654 | 0.154391 | 103 | 3.882635 | 4.826673 | bcd |
| 93_F54 | 4.365942 | 0.154391 | 103 | 3.893924 | 4.837961 | bcd |
| 97_F66 | 4.419436 | 0.154391 | 103 | 3.947417 | 4.891455 | bcde |
| 6_F1 | 4.444702 | 0.166762 | 103 | 3.934863 | 4.954541 | bcde |
| 3_331B | 4.463774 | 0.154391 | 103 | 3.991755 | 4.935793 | bcde |
| 2_159B | 4.52193 | 0.154391 | 103 | 4.049911 | 4.993949 | bcde |
| 8_F5 | 4.633042 | 0.166762 | 103 | 4.123204 | 5.142881 | cde |
| 1_339B | 4.71154 | 0.154391 | 103 | 4.239521 | 5.183559 | cde |
| 95_F83 | 4.721267 | 0.154391 | 103 | 4.249248 | 5.193286 | cde |
| 4_188B | 4.755845 | 0.154391 | 103 | 4.283826 | 5.227864 | cde |
| 7_F2 | 4.905942 | 0.154391 | 103 | 4.433923 | 5.377961 | def |
| 91_F39 | 4.930937 | 0.166762 | 103 | 4.421099 | 5.440776 | def |
| 98_F26B | 5.195824 | 0.154391 | 103 | 4.723805 | 5.667843 | ef |
| 92_F55 | 5.678354 | 0.166762 | 103 | 5.168515 | 6.188193 | f |

**Possible metabolism of phenolic and related compounds (Fig. 4)**

| \| Permutation test for adonis under reduced model  Permutation: free  Number of permutations: 999  adonis2(formula = dist_final ~ Sample, data = data, permutations = 999)  Df SumOfSqs R2 F Pr(>F)  Model 17 1.86017 0.83419 30.779 0.001 ***  Residual 104 0.36973 0.16581  Total 121 2.22990 1.00000  ---  Signif. codes: 0 ‘***’ 0.001 ‘**’ 0.01 ‘*’ 0.05 ‘.’ 0.1 ‘ ’ 1 \| \| --- \| \|  \| \| \| > \| \| --- \| \| |
| --- | --- | --- | --- | --- |

See Suppl. Table S3b for pairwise comparison.

**Possible metabolism of phenolic and related compounds (Fig. 5)**

| Modell |
| --- |
| Normal_log |

| Resid. Df | Resid. Dev | Df | Deviance | Pr(>Chi) | p.value_formatted | Significance |
| --- | --- | --- | --- | --- | --- | --- |
| 121 | 99.99727 |  |  |  | NA |  |
| 104 | 6.531622 | 17 | 93.46565 | 1.7E-306 | 1.7128188313e-306 | *** |

| contrast | estimate | SE | df | t.ratio | p.value | p.value_formatted | Significance |
| --- | --- | --- | --- | --- | --- | --- | --- |
| 1_339M - 2_159M | -0.15977 | 0.133955 | 104 | -1.19274 | 0.999199 | 9.9919906707e-01 |  |
| 1_339M - 3_331M | 1.294805 | 0.133955 | 104 | 9.665949 | 2.58E-13 | 2.5834889783e-13 | *** |
| 1_339M - 4_188M | -0.14779 | 0.133955 | 104 | -1.10326 | 0.999704 | 9.9970429773e-01 |  |
| 1_339M - 5_O.Micolor | 0.242941 | 0.133955 | 104 | 1.8136 | 0.934424 | 9.3442426165e-01 |  |
| 1_339M - 6_F1 | 0.385648 | 0.139425 | 104 | 2.765991 | 0.350624 | 3.5062363214e-01 |  |
| 1_339M - 7_F2 | 2.071977 | 0.133955 | 104 | 15.46768 | 3.51E-14 | 3.5083047578e-14 | *** |
| 1_339M - 8_F5 | 1.240906 | 0.139425 | 104 | 8.900161 | 3.12E-12 | 3.1222802122e-12 | *** |
| 1_339M - 9_F31 | 1.595864 | 0.133955 | 104 | 11.91341 | 6.35E-14 | 6.3504757009e-14 | *** |
| 1_339M - 91_F39 | 0.994814 | 0.133955 | 104 | 7.426465 | 4.87E-09 | 4.8683338472e-09 | *** |
| 1_339M - 92_F55 | -0.78933 | 0.139425 | 104 | -5.66131 | 1.94E-05 | 1.9429919888e-05 | *** |
| 1_339M - 93_F54 | 1.527596 | 0.133955 | 104 | 11.40377 | 9.68E-14 | 9.6811447747e-14 | *** |
| 1_339M - 94_F58 | 1.176116 | 0.133955 | 104 | 8.779915 | 5.6E-12 | 5.6035176499e-12 | *** |
| 1_339M - 95_F83 | -0.34796 | 0.133955 | 104 | -2.59755 | 0.464985 | 4.6498531490e-01 |  |
| 1_339M - 96_F94 | 1.901656 | 0.133955 | 104 | 14.1962 | 3.51E-14 | 3.5083047578e-14 | *** |
| 1_339M - 97_F66 | -0.28497 | 0.133955 | 104 | -2.12739 | 0.793962 | 7.9396212835e-01 |  |
| 1_339M - 98_F26M | 0.02506 | 0.133955 | 104 | 0.187079 | 1 | 1.0000000000e+00 |  |
| 1_339M - 99_Control | -0.47472 | 0.139425 | 104 | -3.40482 | 0.081477 | 8.1477139322e-02 | . |
| 2_159M - 3_331M | 1.454578 | 0.133955 | 104 | 10.85869 | 1.49E-13 | 1.4854784069e-13 | *** |
| 2_159M - 4_188M | 0.011986 | 0.133955 | 104 | 0.089481 | 1 | 1.0000000000e+00 |  |
| 2_159M - 5_O.Micolor | 0.402715 | 0.133955 | 104 | 3.006337 | 0.216791 | 2.1679057799e-01 |  |
| 2_159M - 6_F1 | 0.545422 | 0.139425 | 104 | 3.911935 | 0.017829 | 1.7829145065e-02 | * |
| 2_159M - 7_F2 | 2.231751 | 0.133955 | 104 | 16.66042 | 3.51E-14 | 3.5083047578e-14 | *** |
| 2_159M - 8_F5 | 1.400679 | 0.139425 | 104 | 10.04611 | 2.05E-13 | 2.0472512574e-13 | *** |
| 2_159M - 9_F31 | 1.755637 | 0.133955 | 104 | 13.10614 | 3.73E-14 | 3.7303493627e-14 | *** |
| 2_159M - 91_F39 | 1.154588 | 0.133955 | 104 | 8.619201 | 1.24E-11 | 1.2428613694e-11 | *** |
| 2_159M - 92_F55 | -0.62956 | 0.139425 | 104 | -4.51537 | 0.002136 | 2.1357122222e-03 | ** |
| 2_159M - 93_F54 | 1.687369 | 0.133955 | 104 | 12.59651 | 4.26E-14 | 4.2632564146e-14 | *** |
| 2_159M - 94_F58 | 1.335889 | 0.133955 | 104 | 9.972652 | 2.05E-13 | 2.0516921495e-13 | *** |
| 2_159M - 95_F83 | -0.18818 | 0.133955 | 104 | -1.40481 | 0.99448 | 9.9447980830e-01 |  |
| 2_159M - 96_F94 | 2.061429 | 0.133955 | 104 | 15.38894 | 3.51E-14 | 3.5083047578e-14 | *** |
| 2_159M - 97_F66 | -0.1252 | 0.133955 | 104 | -0.93465 | 0.999969 | 9.9996894770e-01 |  |
| 2_159M - 98_F26M | 0.184834 | 0.133955 | 104 | 1.379816 | 0.995481 | 9.9548061530e-01 |  |
| 2_159M - 99_Control | -0.31494 | 0.139425 | 104 | -2.25887 | 0.709759 | 7.0975872889e-01 |  |
| 3_331M - 4_188M | -1.44259 | 0.133955 | 104 | -10.7692 | 1.51E-13 | 1.5099033135e-13 | *** |
| 3_331M - 5_O.Micolor | -1.05186 | 0.133955 | 104 | -7.85235 | 5.88E-10 | 5.8761318034e-10 | *** |
| 3_331M - 6_F1 | -0.90916 | 0.139425 | 104 | -6.52075 | 3.85E-07 | 3.8520865409e-07 | *** |
| 3_331M - 7_F2 | 0.777172 | 0.133955 | 104 | 5.801731 | 1.04E-05 | 1.0436344889e-05 | *** |
| 3_331M - 8_F5 | -0.0539 | 0.139425 | 104 | -0.38658 | 1 | 9.9999999997e-01 |  |
| 3_331M - 9_F31 | 0.301059 | 0.133955 | 104 | 2.247457 | 0.71751 | 7.1751049702e-01 |  |
| 3_331M - 91_F39 | -0.29999 | 0.133955 | 104 | -2.23948 | 0.722881 | 7.2288082701e-01 |  |
| 3_331M - 92_F55 | -2.08413 | 0.139425 | 104 | -14.9481 | 3.51E-14 | 3.5083047578e-14 | *** |
| 3_331M - 93_F54 | 0.232791 | 0.133955 | 104 | 1.737825 | 0.954314 | 9.5431376859e-01 |  |
| 3_331M - 94_F58 | -0.11869 | 0.133955 | 104 | -0.88603 | 0.999985 | 9.9998546631e-01 |  |
| 3_331M - 95_F83 | -1.64276 | 0.133955 | 104 | -12.2635 | 5.04E-14 | 5.0404125318e-14 | *** |
| 3_331M - 96_F94 | 0.606851 | 0.133955 | 104 | 4.530249 | 0.00202 | 2.0195542809e-03 | ** |
| 3_331M - 97_F66 | -1.57978 | 0.133955 | 104 | -11.7933 | 7.39E-14 | 7.3940853440e-14 | *** |
| 3_331M - 98_F26M | -1.26974 | 0.133955 | 104 | -9.47887 | 3.5E-13 | 3.4994229736e-13 | *** |
| 3_331M - 99_Control | -1.76952 | 0.139425 | 104 | -12.6916 | 4.15E-14 | 4.1522341121e-14 | *** |
| 4_188M - 5_O.Micolor | 0.390728 | 0.133955 | 104 | 2.916856 | 0.261972 | 2.6197200044e-01 |  |
| 4_188M - 6_F1 | 0.533435 | 0.139425 | 104 | 3.825965 | 0.023507 | 2.3506727671e-02 | * |
| 4_188M - 7_F2 | 2.219764 | 0.133955 | 104 | 16.57094 | 3.51E-14 | 3.5083047578e-14 | *** |
| 4_188M - 8_F5 | 1.388693 | 0.139425 | 104 | 9.960136 | 2.02E-13 | 2.0194956818e-13 | *** |
| 4_188M - 9_F31 | 1.743651 | 0.133955 | 104 | 13.01666 | 3.8E-14 | 3.7969627442e-14 | *** |
| 4_188M - 91_F39 | 1.142601 | 0.133955 | 104 | 8.529721 | 1.95E-11 | 1.9485968394e-11 | *** |
| 4_188M - 92_F55 | -0.64154 | 0.139425 | 104 | -4.60134 | 0.001543 | 1.5427066897e-03 | ** |
| 4_188M - 93_F54 | 1.675383 | 0.133955 | 104 | 12.50703 | 4.42E-14 | 4.4186876380e-14 | *** |
| 4_188M - 94_F58 | 1.323903 | 0.133955 | 104 | 9.883171 | 2.2E-13 | 2.1993518118e-13 | *** |
| 4_188M - 95_F83 | -0.20017 | 0.133955 | 104 | -1.49429 | 0.98928 | 9.8928044337e-01 |  |
| 4_188M - 96_F94 | 2.049443 | 0.133955 | 104 | 15.29945 | 3.51E-14 | 3.5083047578e-14 | *** |
| 4_188M - 97_F66 | -0.13719 | 0.133955 | 104 | -1.02413 | 0.99989 | 9.9989016771e-01 |  |
| 4_188M - 98_F26M | 0.172847 | 0.133955 | 104 | 1.290335 | 0.997915 | 9.9791469822e-01 |  |
| 4_188M - 99_Control | -0.32693 | 0.139425 | 104 | -2.34484 | 0.649419 | 6.4941911046e-01 |  |
| 5_O.Micolor - 6_F1 | 0.142707 | 0.139425 | 104 | 1.02354 | 0.999891 | 9.9989102323e-01 |  |
| 5_O.Micolor - 7_F2 | 1.829036 | 0.133955 | 104 | 13.65408 | 3.57E-14 | 3.5749181393e-14 | *** |
| 5_O.Micolor - 8_F5 | 0.997964 | 0.139425 | 104 | 7.157711 | 1.82E-08 | 1.8171383620e-08 | *** |
| 5_O.Micolor - 9_F31 | 1.352922 | 0.133955 | 104 | 10.09981 | 1.98E-13 | 1.9784174299e-13 | *** |
| 5_O.Micolor - 91_F39 | 0.751873 | 0.133955 | 104 | 5.612864 | 2.4E-05 | 2.4030044164e-05 | *** |
| 5_O.Micolor - 92_F55 | -1.03227 | 0.139425 | 104 | -7.40377 | 5.44E-09 | 5.4442349517e-09 | *** |
| 5_O.Micolor - 93_F54 | 1.284654 | 0.133955 | 104 | 9.590174 | 2.91E-13 | 2.9087843245e-13 | *** |
| 5_O.Micolor - 94_F58 | 0.933175 | 0.133955 | 104 | 6.966315 | 4.6E-08 | 4.5983064800e-08 | *** |
| 5_O.Micolor - 95_F83 | -0.5909 | 0.133955 | 104 | -4.41115 | 0.003146 | 3.1456231343e-03 | ** |
| 5_O.Micolor - 96_F94 | 1.658714 | 0.133955 | 104 | 12.3826 | 4.82E-14 | 4.8183679269e-14 | *** |
| 5_O.Micolor - 97_F66 | -0.52792 | 0.133955 | 104 | -3.94099 | 0.016213 | 1.6213095451e-02 | * |
| 5_O.Micolor - 98_F26M | -0.21788 | 0.133955 | 104 | -1.62652 | 0.975022 | 9.7502163339e-01 |  |
| 5_O.Micolor - 99_Control | -0.71766 | 0.139425 | 104 | -5.14727 | 0.000175 | 1.7502030020e-04 | *** |
| 6_F1 - 7_F2 | 1.686329 | 0.139425 | 104 | 12.09488 | 5.91E-14 | 5.9063864910e-14 | *** |
| 6_F1 - 8_F5 | 0.855257 | 0.144688 | 104 | 5.911035 | 6.4E-06 | 6.3972967583e-06 | *** |
| 6_F1 - 9_F31 | 1.210215 | 0.139425 | 104 | 8.68004 | 9.18E-12 | 9.1836538374e-12 | *** |
| 6_F1 - 91_F39 | 0.609166 | 0.139425 | 104 | 4.369126 | 0.003669 | 3.6688352689e-03 | ** |
| 6_F1 - 92_F55 | -1.17498 | 0.144688 | 104 | -8.12075 | 1.53E-10 | 1.5291534705e-10 | *** |
| 6_F1 - 93_F54 | 1.141947 | 0.139425 | 104 | 8.190401 | 1.08E-10 | 1.0769451997e-10 | *** |
| 6_F1 - 94_F58 | 0.790467 | 0.139425 | 104 | 5.669479 | 1.87E-05 | 1.8744636856e-05 | *** |
| 6_F1 - 95_F83 | -0.7336 | 0.139425 | 104 | -5.26164 | 0.000109 | 1.0854661222e-04 | *** |
| 6_F1 - 96_F94 | 1.516007 | 0.139425 | 104 | 10.87328 | 1.45E-13 | 1.4499512702e-13 | *** |
| 6_F1 - 97_F66 | -0.67062 | 0.139425 | 104 | -4.80992 | 0.000686 | 6.8638116120e-04 | *** |
| 6_F1 - 98_F26M | -0.36059 | 0.139425 | 104 | -2.58625 | 0.473078 | 4.7307807951e-01 |  |
| 6_F1 - 99_Control | -0.86037 | 0.144688 | 104 | -5.94634 | 5.46E-06 | 5.4563646460e-06 | *** |
| 7_F2 - 8_F5 | -0.83107 | 0.139425 | 104 | -5.9607 | 5.11E-06 | 5.1136773704e-06 | *** |
| 7_F2 - 9_F31 | -0.47611 | 0.133955 | 104 | -3.55427 | 0.053603 | 5.3603071958e-02 | . |
| 7_F2 - 91_F39 | -1.07716 | 0.133955 | 104 | -8.04122 | 2.28E-10 | 2.2807200573e-10 | *** |
| 7_F2 - 92_F55 | -2.86131 | 0.139425 | 104 | -20.5222 | 3.51E-14 | 3.5083047578e-14 | *** |
| 7_F2 - 93_F54 | -0.54438 | 0.133955 | 104 | -4.06391 | 0.010754 | 1.0753583701e-02 | * |
| 7_F2 - 94_F58 | -0.89586 | 0.133955 | 104 | -6.68776 | 1.75E-07 | 1.7475582192e-07 | *** |
| 7_F2 - 95_F83 | -2.41993 | 0.133955 | 104 | -18.0652 | 3.51E-14 | 3.5083047578e-14 | *** |
| 7_F2 - 96_F94 | -0.17032 | 0.133955 | 104 | -1.27148 | 0.99825 | 9.9824975324e-01 |  |
| 7_F2 - 97_F66 | -2.35695 | 0.133955 | 104 | -17.5951 | 3.51E-14 | 3.5083047578e-14 | *** |
| 7_F2 - 98_F26M | -2.04692 | 0.133955 | 104 | -15.2806 | 3.51E-14 | 3.5083047578e-14 | *** |
| 7_F2 - 99_Control | -2.54669 | 0.139425 | 104 | -18.2657 | 3.51E-14 | 3.5083047578e-14 | *** |
| 8_F5 - 9_F31 | 0.354958 | 0.139425 | 104 | 2.545869 | 0.502284 | 5.0228406754e-01 |  |
| 8_F5 - 91_F39 | -0.24609 | 0.139425 | 104 | -1.76504 | 0.947752 | 9.4775231123e-01 |  |
| 8_F5 - 92_F55 | -2.03023 | 0.144688 | 104 | -14.0318 | 3.53E-14 | 3.5305092183e-14 | *** |
| 8_F5 - 93_F54 | 0.28669 | 0.139425 | 104 | 2.05623 | 0.833975 | 8.3397474861e-01 |  |
| 8_F5 - 94_F58 | -0.06479 | 0.139425 | 104 | -0.46469 | 1 | 9.9999999933e-01 |  |
| 8_F5 - 95_F83 | -1.58886 | 0.139425 | 104 | -11.3958 | 9.77E-14 | 9.7699626167e-14 | *** |
| 8_F5 - 96_F94 | 0.66075 | 0.139425 | 104 | 4.739105 | 0.000906 | 9.0647893678e-04 | *** |
| 8_F5 - 97_F66 | -1.52588 | 0.139425 | 104 | -10.9441 | 1.33E-13 | 1.3322676296e-13 | *** |
| 8_F5 - 98_F26M | -1.21585 | 0.139425 | 104 | -8.72042 | 7.51E-12 | 7.5092154717e-12 | *** |
| 8_F5 - 99_Control | -1.71562 | 0.144688 | 104 | -11.8574 | 6.93E-14 | 6.9277916737e-14 | *** |
| 9_F31 - 91_F39 | -0.60105 | 0.133955 | 104 | -4.48694 | 0.002375 | 2.3754964118e-03 | ** |
| 9_F31 - 92_F55 | -2.38519 | 0.139425 | 104 | -17.1073 | 3.51E-14 | 3.5083047578e-14 | *** |
| 9_F31 - 93_F54 | -0.06827 | 0.133955 | 104 | -0.50963 | 1 | 9.9999999701e-01 |  |
| 9_F31 - 94_F58 | -0.41975 | 0.133955 | 104 | -3.13349 | 0.162378 | 1.6237790257e-01 |  |
| 9_F31 - 95_F83 | -1.94382 | 0.133955 | 104 | -14.511 | 3.51E-14 | 3.5083047578e-14 | *** |
| 9_F31 - 96_F94 | 0.305792 | 0.133955 | 104 | 2.282792 | 0.693298 | 6.9329799498e-01 |  |
| 9_F31 - 97_F66 | -1.88084 | 0.133955 | 104 | -14.0408 | 3.53E-14 | 3.5305092183e-14 | *** |
| 9_F31 - 98_F26M | -1.5708 | 0.133955 | 104 | -11.7263 | 7.51E-14 | 7.5051076465e-14 | *** |
| 9_F31 - 99_Control | -2.07058 | 0.139425 | 104 | -14.8508 | 3.51E-14 | 3.5083047578e-14 | *** |
| 91_F39 - 92_F55 | -1.78414 | 0.139425 | 104 | -12.7964 | 4.04E-14 | 4.0412118096e-14 | *** |
| 91_F39 - 93_F54 | 0.532782 | 0.133955 | 104 | 3.977309 | 0.014381 | 1.4381221312e-02 | * |
| 91_F39 - 94_F58 | 0.181302 | 0.133955 | 104 | 1.35345 | 0.996367 | 9.9636711648e-01 |  |
| 91_F39 - 95_F83 | -1.34277 | 0.133955 | 104 | -10.024 | 2.1E-13 | 2.1027624086e-13 | *** |
| 91_F39 - 96_F94 | 0.906842 | 0.133955 | 104 | 6.769734 | 1.18E-07 | 1.1822734136e-07 | *** |
| 91_F39 - 97_F66 | -1.27979 | 0.133955 | 104 | -9.55385 | 3.07E-13 | 3.0697666631e-13 | *** |
| 91_F39 - 98_F26M | -0.96975 | 0.133955 | 104 | -7.23939 | 1.22E-08 | 1.2196627597e-08 | *** |
| 91_F39 - 99_Control | -1.46953 | 0.139425 | 104 | -10.5399 | 1.65E-13 | 1.6475709685e-13 | *** |
| 92_F55 - 93_F54 | 2.316925 | 0.139425 | 104 | 16.61771 | 3.51E-14 | 3.5083047578e-14 | *** |
| 92_F55 - 94_F58 | 1.965445 | 0.139425 | 104 | 14.09678 | 3.53E-14 | 3.5305092183e-14 | *** |
| 92_F55 - 95_F83 | 0.441374 | 0.139425 | 104 | 3.165668 | 0.150386 | 1.5038604652e-01 |  |
| 92_F55 - 96_F94 | 2.690985 | 0.139425 | 104 | 19.30058 | 3.51E-14 | 3.5083047578e-14 | *** |
| 92_F55 - 97_F66 | 0.504354 | 0.139425 | 104 | 3.617387 | 0.044573 | 4.4573089704e-02 | * |
| 92_F55 - 98_F26M | 0.814389 | 0.139425 | 104 | 5.841054 | 8.76E-06 | 8.7562854929e-06 | *** |
| 92_F55 - 99_Control | 0.314612 | 0.144688 | 104 | 2.174414 | 0.765229 | 7.6522874655e-01 |  |
| 93_F54 - 94_F58 | -0.35148 | 0.133955 | 104 | -2.62386 | 0.446306 | 4.4630561479e-01 |  |
| 93_F54 - 95_F83 | -1.87555 | 0.133955 | 104 | -14.0013 | 3.53E-14 | 3.5305092183e-14 | *** |
| 93_F54 - 96_F94 | 0.37406 | 0.133955 | 104 | 2.792425 | 0.334028 | 3.3402782288e-01 |  |
| 93_F54 - 97_F66 | -1.81257 | 0.133955 | 104 | -13.5312 | 3.57E-14 | 3.5749181393e-14 | *** |
| 93_F54 - 98_F26M | -1.50254 | 0.133955 | 104 | -11.2167 | 1.14E-13 | 1.1413092693e-13 | *** |
| 93_F54 - 99_Control | -2.00231 | 0.139425 | 104 | -14.3612 | 3.51E-14 | 3.5083047578e-14 | *** |
| 94_F58 - 95_F83 | -1.52407 | 0.133955 | 104 | -11.3775 | 1.02E-13 | 1.0191847366e-13 | *** |
| 94_F58 - 96_F94 | 0.72554 | 0.133955 | 104 | 5.416284 | 5.63E-05 | 5.6291792767e-05 | *** |
| 94_F58 - 97_F66 | -1.46109 | 0.133955 | 104 | -10.9073 | 1.41E-13 | 1.4099832413e-13 | *** |
| 94_F58 - 98_F26M | -1.15106 | 0.133955 | 104 | -8.59284 | 1.42E-11 | 1.4188317188e-11 | *** |
| 94_F58 - 99_Control | -1.65083 | 0.139425 | 104 | -11.8403 | 7.15E-14 | 7.1498362786e-14 | *** |
| 95_F83 - 96_F94 | 2.249611 | 0.133955 | 104 | 16.79375 | 3.51E-14 | 3.5083047578e-14 | *** |
| 95_F83 - 97_F66 | 0.062981 | 0.133955 | 104 | 0.470164 | 1 | 9.9999999919e-01 |  |
| 95_F83 - 98_F26M | 0.373016 | 0.133955 | 104 | 2.78463 | 0.338878 | 3.3887751816e-01 |  |
| 95_F83 - 99_Control | -0.12676 | 0.139425 | 104 | -0.90917 | 0.999979 | 9.9997899660e-01 |  |
| 96_F94 - 97_F66 | -2.18663 | 0.133955 | 104 | -16.3236 | 3.51E-14 | 3.5083047578e-14 | *** |
| 96_F94 - 98_F26M | -1.8766 | 0.133955 | 104 | -14.0091 | 3.53E-14 | 3.5305092183e-14 | *** |
| 96_F94 - 99_Control | -2.37637 | 0.139425 | 104 | -17.0441 | 3.51E-14 | 3.5083047578e-14 | *** |
| 97_F66 - 98_F26M | 0.310035 | 0.133955 | 104 | 2.314466 | 0.671089 | 6.7108924338e-01 |  |
| 97_F66 - 99_Control | -0.18974 | 0.139425 | 104 | -1.36089 | 0.996133 | 9.9613318456e-01 |  |
| 98_F26M - 99_Control | -0.49978 | 0.139425 | 104 | -3.58456 | 0.049089 | 4.9089134897e-02 | * |

| Sample | emmean | SE | df | lower.CL | upper.CL | .group |
| --- | --- | --- | --- | --- | --- | --- |
| 7_F2 | 4.734482 | 0.094721 | 104 | 4.444961 | 5.024002 | a |
| 96_F94 | 4.904803 | 0.094721 | 104 | 4.615283 | 5.194324 | ab |
| 9_F31 | 5.210595 | 0.094721 | 104 | 4.921075 | 5.500116 | abc |
| 93_F54 | 5.278863 | 0.094721 | 104 | 4.989343 | 5.568384 | bc |
| 3_331M | 5.511654 | 0.094721 | 104 | 5.222134 | 5.801175 | cd |
| 8_F5 | 5.565553 | 0.10231 | 104 | 5.252836 | 5.878271 | cd |
| 94_F58 | 5.630343 | 0.094721 | 104 | 5.340823 | 5.919863 | cd |
| 91_F39 | 5.811645 | 0.094721 | 104 | 5.522124 | 6.101165 | d |
| 6_F1 | 6.420811 | 0.10231 | 104 | 6.108093 | 6.733528 | e |
| 5_O.Micolor | 6.563518 | 0.094721 | 104 | 6.273997 | 6.853038 | ef |
| 98_F26M | 6.781399 | 0.094721 | 104 | 6.491878 | 7.070919 | efg |
| 1_339M | 6.806459 | 0.094721 | 104 | 6.516939 | 7.095979 | efgh |
| 4_188M | 6.954246 | 0.094721 | 104 | 6.664726 | 7.243766 | fgh |
| 2_159M | 6.966232 | 0.094721 | 104 | 6.676712 | 7.255753 | fgh |
| 97_F66 | 7.091434 | 0.094721 | 104 | 6.801913 | 7.380954 | gh |
| 95_F83 | 7.154415 | 0.094721 | 104 | 6.864894 | 7.443935 | ghi |
| 99_Control | 7.281176 | 0.10231 | 104 | 6.968458 | 7.593894 | hi |
| 92_F55 | 7.595788 | 0.10231 | 104 | 7.28307 | 7.908506 | i |

**Inhibition assays (Fig. 6)**

***R. sulphurea***

| **Modell** |
| --- |
| Normal_log |

| **contrast** | **estimate** | **SE** | **df** | **t.ratio** | **p.value** | **p.value_formatted** |
| --- | --- | --- | --- | --- | --- | --- |
| 159 Catechin - DMSO_Control | 0.005787 | 0.11279 | 42 | 0.051308 | 0.999959 | 9.9995889861e-01 |
| 159 Gallic acid - DMSO_Control | 0.019183 | 0.11279 | 42 | 0.170075 | 0.998518 | 9.9851773976e-01 |
| 159 Protocatechuic acid - DMSO_Control | -0.0802 | 0.11279 | 42 | -0.71105 | 0.906874 | 9.0687365741e-01 |
| 159 Quinic acid - DMSO_Control | -0.16185 | 0.11279 | 42 | -1.43496 | 0.511925 | 5.1192490910e-01 |
| 159 Shikimic acid - DMSO_Control | -0.02609 | 0.11279 | 42 | -0.23129 | 0.996305 | 9.9630487522e-01 |
| 159 Vanillic acid - DMSO_Control | -0.12022 | 0.11279 | 42 | -1.06588 | 0.739013 | 7.3901317926e-01 |

***E. polonica***

| **Modell** |
| --- |
| Normal_log |

| **contrast** | **estimate** | **SE** | **df** | **t.ratio** | **p.value** | **p.value_formatted** | **Signif** |
| --- | --- | --- | --- | --- | --- | --- | --- |
| 331 Catechin - DMSO_Control | 0.231633 | 0.099293 | 41 | 2.332827 | 0.112469 | 1.1246941646e-01 |  |
| 331 Gallic acid - DMSO_Control | -0.16854 | 0.099293 | 41 | -1.69743 | 0.358171 | 3.5817084225e-01 |  |
| 331 Protocatechuic acid - DMSO_Control | -0.04668 | 0.099293 | 41 | -0.47016 | 0.970635 | 9.7063522367e-01 |  |
| 331 Quinic acid - DMSO_Control | 0.025694 | 0.099293 | 41 | 0.258768 | 0.994848 | 9.9484778987e-01 |  |
| 331 Shikimic acid - DMSO_Control | 0.345256 | 0.099293 | 41 | 3.477155 | 0.006551 | 6.5510942964e-03 | ** |
| 331 Vanillic acid - DMSO_Control | 0.070686 | 0.103347 | 41 | 0.683964 | 0.916171 | 9.1617051649e-01 |  |

***Penicillium bialowiezense***

| **Modell** |
| --- |
| Normal_log |

| **contrast** | **estimate** | **SE** | **df** | **t.ratio** | **p.value** | **p.value_formatted** | **Signif** |
| --- | --- | --- | --- | --- | --- | --- | --- |
| F54B Catechin - DMSO_Control | -0.14492 | 0.131423 | 42 | -1.10267 | 0.717651 | 7.1765053279e-01 |  |
| F54B Gallic acid - DMSO_Control | -0.20415 | 0.131423 | 42 | -1.5534 | 0.439764 | 4.3976440135e-01 |  |
| F54B Protocatechuic acid - DMSO_Control | -0.29161 | 0.131423 | 42 | -2.2189 | 0.141782 | 1.4178212097e-01 |  |
| F54B Quinic acid - DMSO_Control | -0.49107 | 0.131423 | 42 | -3.73655 | 0.003069 | 3.0687353040e-03 | ** |
| F54B Shikimic acid - DMSO_Control | -0.58304 | 0.131423 | 42 | -4.43635 | 0.00037 | 3.6968760179e-04 | *** |
| F54B Vanillic acid - DMSO_Control | -0.27278 | 0.131423 | 42 | -2.0756 | 0.187647 | 1.8764676899e-01 |  |

***O. piceae***

| **Modell** |
| --- |
| Normal_log |

| **contrast** | **estimate** | **SE** | **df** | **t.ratio** | **p.value** | **p.value_formatted** | **Signif** |
| --- | --- | --- | --- | --- | --- | --- | --- |
| F2 Catechin - DMSO_Control | -0.03789 | 0.071593 | 42 | -0.52924 | 0.958894 | 9.5889374889e-01 |  |
| F2 Gallic acid - DMSO_Control | 0.077536 | 0.071593 | 42 | 1.083012 | 0.729133 | 7.2913269776e-01 |  |
| F2 Protocatechuic acid - DMSO_Control | 0.011114 | 0.071593 | 42 | 0.155231 | 0.998871 | 9.9887094918e-01 |  |
| F2 Quinic acid - DMSO_Control | -0.28577 | 0.071593 | 42 | -3.99163 | 0.001444 | 1.4435450446e-03 | ** |
| F2 Shikimic acid - DMSO_Control | 0.05092 | 0.071593 | 42 | 0.711237 | 0.906807 | 9.0680738159e-01 |  |
| F2 Vanillic acid - DMSO_Control | 0.155633 | 0.071593 | 42 | 2.17385 | 0.155143 | 1.5514330948e-01 |  |

***Geosmithia* sp*.***

| Modell |
| --- |
| Normal_log |

| contrast | estimate | SE | df | t.ratio | p.value | p.value_formatted | Signif |
| --- | --- | --- | --- | --- | --- | --- | --- |
| F1 Catechin - DMSO_Control | 0.098876 | 0.090371 | 41 | 1.094112 | 0.722735 | 7.2273547006e-01 |  |
| F1 Gallic acid - DMSO_Control | -0.23604 | 0.094062 | 41 | -2.50946 | 0.076585 | 7.6585135451e-02 | . |
| F1 Protocatechuic acid - DMSO_Control | 0.153933 | 0.090371 | 41 | 1.703341 | 0.354986 | 3.5498569618e-01 |  |
| F1 Quinic acid - DMSO_Control | -0.02202 | 0.090371 | 41 | -0.24366 | 0.995687 | 9.9568690923e-01 |  |
| F1 Shikimic acid - DMSO_Control | 0.089665 | 0.090371 | 41 | 0.992186 | 0.780054 | 7.8005424321e-01 |  |
| F1 Vanillic acid - DMSO_Control | 0.181822 | 0.090371 | 41 | 2.011944 | 0.211703 | 2.1170343331e-01 |  |

***Blastobotrys* sp.**

| **Modell** |
| --- |
| Normal_log |

| **contrast** | **estimate** | **SE** | **df** | **t.ratio** | **p.value** | **p.value_formatted** | **Signif** |
| --- | --- | --- | --- | --- | --- | --- | --- |
| F55 Catechin - DMSO_Control | 0.074939 | 0.131516 | 40 | 0.569807 | 0.949377 | 9.4937659344e-01 |  |
| F55 Gallic acid - DMSO_Control | 0.362229 | 0.131516 | 40 | 2.754251 | 0.043647 | 4.3646572855e-02 | * |
| F55 Protocatechuic acid - DMSO_Control | -0.02584 | 0.136886 | 40 | -0.1888 | 0.997975 | 9.9797549452e-01 |  |
| F55 Quinic acid - DMSO_Control | 0.223247 | 0.131516 | 40 | 1.697487 | 0.358519 | 3.5851915728e-01 |  |
| F55 Shikimic acid - DMSO_Control | 0.280824 | 0.136886 | 40 | 2.051509 | 0.197263 | 1.9726257341e-01 |  |
| F55 Vanillic acid - DMSO_Control | -0.24481 | 0.131516 | 40 | -1.86146 | 0.276129 | 2.7612855509e-01 |  |

**Catabolism assays (Suppl. Fig. S6)**

**Vanillic acid**

| Modell |
| --- |
| Sqrt_Normal |

| Resid. Df | Resid. Dev | Df | Deviance | Pr(>Chi) | p.value_formatted | Significance |
| --- | --- | --- | --- | --- | --- | --- |
| 33 | 3112.928 |  |  |  | NA |  |
| 29 | 31.72668 | 4 | 3081.201 | 0 | 0.0000000000e+00 | *** |

| contrast | estimate | SE | df | t.ratio | p.value | p.value_formatted | Significance |
| --- | --- | --- | --- | --- | --- | --- | --- |
| Control - F1 | 16.96516 | 0.581916 | 29 | 29.15397 | 3.55E-15 | 3.5527136788e-15 | *** |
| Control - F2 | 16.93554 | 0.581916 | 29 | 29.10306 | 3.55E-15 | 3.5527136788e-15 | *** |
| Control - F54B | 13.01474 | 0.581916 | 29 | 22.36533 | 3.55E-15 | 3.5527136788e-15 | *** |
| Control - F55 | -6.16636 | 0.581916 | 29 | -10.5966 | 1.7E-10 | 1.7043377820e-10 | *** |
| F1 - F2 | -0.02963 | 0.559087 | 29 | -0.05299 | 0.999998 | 9.9999809319e-01 | |
| F1 - F54B | -3.95042 | 0.559087 | 29 | -7.06585 | 8.59E-07 | 8.5936809335e-07 | *** |
| F1 - F55 | -23.1315 | 0.559087 | 29 | -41.3738 | 3.55E-15 | 3.5527136788e-15 | *** |
| F2 - F54B | -3.92079 | 0.559087 | 29 | -7.01285 | 9.88E-07 | 9.8806051341e-07 | *** |
| F2 - F55 | -23.1019 | 0.559087 | 29 | -41.3208 | 3.55E-15 | 3.5527136788e-15 | *** |
| F54B - F55 | -19.1811 | 0.559087 | 29 | -34.3079 | 3.55E-15 | 3.5527136788e-15 | *** |

| Sample | emmean | SE | df | lower.CL | upper.CL | .group |
| --- | --- | --- | --- | --- | --- | --- |
| F1 | 2.468291 | 0.395334 | 29 | 1.381945 | 3.554636 | a |
| F2 | 2.497917 | 0.395334 | 29 | 1.411572 | 3.584263 | a |
| F54B | 6.418712 | 0.395334 | 29 | 5.332367 | 7.505057 | b |
| Control | 19.43345 | 0.42701 | 29 | 18.26007 | 20.60684 | c |
| F55 | 25.59981 | 0.395334 | 29 | 24.51347 | 26.68616 | d |

**Quinic acid**

| Modell |
| --- |
| Normal_log |

| Resid. Df | Resid. Dev | Df | Deviance | Pr(>Chi) | p.value_formatted | Significance |
| --- | --- | --- | --- | --- | --- | --- |
| 33 | 135.6291 |  |  |  | NA |  |
| 29 | 8.179117 | 4 | 127.45 | 1.7E-96 | 1.6963987814e-96 | *** |

| contrast | estimate | SE | df | t.ratio | p.value | p.value_formatted | Significance |
| --- | --- | --- | --- | --- | --- | --- | --- |
| Control - F1 | -0.2296 | 0.295462 | 29 | -0.77709 | 0.935184 | 9.3518365509e-01 | |
| Control - F2 | 4.163179 | 0.295462 | 29 | 14.09042 | 1.71E-13 | 1.7075230119e-13 | *** |
| Control - F54B | 3.271575 | 0.295462 | 29 | 11.07275 | 6.09E-11 | 6.0907723309e-11 | *** |
| Control - F55 | -0.25477 | 0.295462 | 29 | -0.86227 | 0.908239 | 9.0823904767e-01 | |
| F1 - F2 | 4.392779 | 0.28387 | 29 | 15.47459 | 1.85E-14 | 1.8540724511e-14 | *** |
| F1 - F54B | 3.501175 | 0.28387 | 29 | 12.33371 | 4.57E-12 | 4.5713433039e-12 | *** |
| F1 - F55 | -0.02517 | 0.28387 | 29 | -0.08866 | 0.999985 | 9.9998509804e-01 | |
| F2 - F54B | -0.8916 | 0.28387 | 29 | -3.14088 | 0.029219 | 2.9218753008e-02 | * |
| F2 - F55 | -4.41795 | 0.28387 | 29 | -15.5632 | 1.64E-14 | 1.6431300764e-14 | *** |
| F54B - F55 | -3.52634 | 0.28387 | 29 | -12.4224 | 3.84E-12 | 3.8384850853e-12 | *** |

| Sample | emmean | SE | df | lower.CL | upper.CL | .group |
| --- | --- | --- | --- | --- | --- | --- |
| F2 | 3.049245 | 0.200727 | 29 | 2.497664 | 3.600825 | a |
| F54B | 3.940848 | 0.200727 | 29 | 3.389268 | 4.492429 | b |
| Control | 7.212424 | 0.21681 | 29 | 6.616649 | 7.808199 | c |
| F1 | 7.442023 | 0.200727 | 29 | 6.890443 | 7.993604 | c |
| F55 | 7.467191 | 0.200727 | 29 | 6.915611 | 8.018772 | c |

**Catechin**

| Modell |
| --- |
| Sqrt_Normal |
|  |

| Resid. Df | Resid. Dev | Df | Deviance | Pr(>Chi) | p.value_formatted | Significance |
| --- | --- | --- | --- | --- | --- | --- |
| 33 | 4588.359 |  |  |  | NA |  |
| 29 | 607.3698 | 4 | 3980.989 | 5.1E-40 | 5.0951604695e-40 | *** |

| contrast | estimate | SE | df | t.ratio | p.value | p.value_formatted | Significance |
| --- | --- | --- | --- | --- | --- | --- | --- |
| Control - F1 | 10.4932 | 2.546095 | 29 | 4.12129 | 0.002472 | 2.4721800348e-03 | ** |
| Control - F2 | 18.26447 | 2.546095 | 29 | 7.173523 | 6.48E-07 | 6.4778226472e-07 | *** |
| Control - F54B | 22.64927 | 2.546095 | 29 | 8.895688 | 8.5E-09 | 8.4979498993e-09 | *** |
| Control - F55 | -6.01666 | 2.546095 | 29 | -2.36309 | 0.154474 | 1.5447433158e-01 | |
| F1 - F2 | 7.771276 | 2.446209 | 29 | 3.176865 | 0.026842 | 2.6841793723e-02 | * |
| F1 - F54B | 12.15607 | 2.446209 | 29 | 4.969351 | 0.000251 | 2.5057218800e-04 | *** |
| F1 - F55 | -16.5099 | 2.446209 | 29 | -6.74916 | 1.99E-06 | 1.9870383535e-06 | *** |
| F2 - F54B | 4.384794 | 2.446209 | 29 | 1.792486 | 0.397007 | 3.9700729174e-01 | |
| F2 - F55 | -24.2811 | 2.446209 | 29 | -9.92602 | 7.62E-10 | 7.6165440621e-10 | *** |
| F54B - F55 | -28.6659 | 2.446209 | 29 | -11.7185 | 1.58E-11 | 1.5775714068e-11 | *** |

| Sample | emmean | SE | df | lower.CL | upper.CL | .group |
| --- | --- | --- | --- | --- | --- | --- |
| F54B | 3.03322 | 1.729731 | 29 | -1.71994 | 7.786378 | a |
| F2 | 7.418014 | 1.729731 | 29 | 2.664856 | 12.17117 | a |
| F1 | 15.18929 | 1.729731 | 29 | 10.43613 | 19.94245 | b |
| Control | 25.68249 | 1.868323 | 29 | 20.54849 | 30.81648 | c |
| F55 | 31.69914 | 1.729731 | 29 | 26.94598 | 36.4523 | c |

**Gallic acid**

| Modell |
| --- |
| Sqrt_Normal |

| Resid. Df | Resid. Dev | Df | Deviance | Pr(>Chi) | p.value_formatted | Significance |
| --- | --- | --- | --- | --- | --- | --- |
| 32 | 11720.39 |  |  |  | NA |  |
| 28 | 1327.35 | 4 | 10393.04 | 2.74E-46 | 2.7352179723e-46 | *** |

| contrast | estimate | SE | df | t.ratio | p.value | p.value_formatted | Significance |
| --- | --- | --- | --- | --- | --- | --- | --- |
| Control - F1 | 26.7838 | 3.975146 | 28 | 6.737815 | 2.44E-06 | 2.4425422476e-06 | *** |
| Control - F2 | 15.08848 | 3.830547 | 28 | 3.938988 | 0.004164 | 4.1637706008e-03 | ** |
| Control - F54B | 36.8717 | 3.830547 | 28 | 9.625701 | 2.16E-09 | 2.1558602681e-09 | *** |
| Control - F55 | -11.5702 | 3.830547 | 28 | -3.0205 | 0.039312 | 3.9311617064e-02 | * |
| F1 - F2 | -11.6953 | 3.830547 | 28 | -3.05317 | 0.036488 | 3.6488449597e-02 | * |
| F1 - F54B | 10.0879 | 3.830547 | 28 | 2.63354 | 0.091143 | 9.1142782474e-02 | . |
| F1 - F55 | -38.354 | 3.830547 | 28 | -10.0127 | 9.11E-10 | 9.1061502783e-10 | *** |
| F2 - F54B | 21.78322 | 3.68027 | 28 | 5.918919 | 2.12E-05 | 2.1243061886e-05 | *** |
| F2 - F55 | -26.6586 | 3.68027 | 28 | -7.24367 | 6.62E-07 | 6.6243883923e-07 | *** |
| F54B - F55 | -48.4419 | 3.68027 | 28 | -13.1626 | 1.6E-12 | 1.5979439993e-12 | *** |

| Sample | emmean | SE | df | lower.CL | upper.CL | .group |
| --- | --- | --- | --- | --- | --- | --- |
| F54B | 5.195619 | 2.602344 | 28 | -1.97316 | 12.3644 | a |
| F1 | 15.28352 | 2.810853 | 28 | 7.540353 | 23.02668 | a |
| F2 | 26.97884 | 2.602344 | 28 | 19.81006 | 34.14762 | b |
| Control | 42.06732 | 2.810853 | 28 | 34.32415 | 49.81048 | c |
| F55 | 53.63749 | 2.602344 | 28 | 46.46871 | 60.80627 | d |

**Protocatechuic acid**

| Modell |
| --- |
| Sqrt_Normal |

| Resid. Df | Resid. Dev | Df | Deviance | Pr(>Chi) | p.value_formatted | Significance |
| --- | --- | --- | --- | --- | --- | --- |
| 32 | 15830.08 |  |  |  | NA |  |
| 28 | 715.0381 | 4 | 15115.04 | 8.8E-127 | 8.8361955359e-127 | *** |

| contrast | estimate | SE | df | t.ratio | p.value | p.value_formatted | Significance |
| --- | --- | --- | --- | --- | --- | --- | --- |
| Control - F1 | 31.64531 | 2.811465 | 28 | 11.25581 | 6.48E-11 | 6.4817595735e-11 | *** |
| Control - F2 | 38.02123 | 2.811465 | 28 | 13.52364 | 8.22E-13 | 8.2189810513e-13 | *** |
| Control - F54B | 34.61223 | 2.811465 | 28 | 12.3111 | 7.96E-12 | 7.9637407779e-12 | *** |
| Control - F55 | -16.5698 | 2.917595 | 28 | -5.67925 | 4.04E-05 | 4.0355851716e-05 | *** |
| F1 - F2 | 6.375915 | 2.701168 | 28 | 2.360429 | 0.15624 | 1.5624019107e-01 | |
| F1 - F54B | 2.966915 | 2.701168 | 28 | 1.098382 | 0.805864 | 8.0586377217e-01 | |
| F1 - F55 | -48.2151 | 2.811465 | 28 | -17.1495 | 0 | 0.0000000000e+00 | *** |
| F2 - F54B | -3.409 | 2.701168 | 28 | -1.26205 | 0.715762 | 7.1576222706e-01 | |
| F2 - F55 | -54.591 | 2.811465 | 28 | -19.4173 | 0 | 0.0000000000e+00 | *** |
| F54B - F55 | -51.182 | 2.811465 | 28 | -18.2047 | 0 | 0.0000000000e+00 | *** |
|  |  |  |  |  |  |  |  |

| Sample | emmean | SE | df | lower.CL | upper.CL | .group |
| --- | --- | --- | --- | --- | --- | --- |
| F2 | 7.675874 | 1.910014 | 28 | 2.414284 | 12.93746 | a |
| F54B | 11.08487 | 1.910014 | 28 | 5.823284 | 16.34646 | a |
| F1 | 14.05179 | 1.910014 | 28 | 8.790199 | 19.31338 | a |
| Control | 45.6971 | 2.063051 | 28 | 40.01394 | 51.38027 | b |
| F55 | 62.26686 | 2.063051 | 28 | 56.58369 | 67.95003 | c |

**Shikimic acid**

| Modell |
| --- |
| Sqrt_Normal |

| Resid. Df | Resid. Dev | Df | Deviance | Pr(>Chi) | p.value_formatted | Significance |
| --- | --- | --- | --- | --- | --- | --- |
| 32 | 6119.782 |  |  |  | NA |  |
| 28 | 130.4 | 4 | 5989.382 | 3.5E-277 | 3.4981892417e-277 | *** |

| contrast | estimate | SE | df | t.ratio | p.value | p.value_formatted | Significance |
| --- | --- | --- | --- | --- | --- | --- | --- |
| Control - F1 | -5.96584 | 1.200624 | 28 | -4.96895 | 0.000273 | 2.7268077881e-04 | *** |
| Control - F2 | -4.84545 | 1.200624 | 28 | -4.03578 | 0.003242 | 3.2421963230e-03 | ** |
| Control - F54B | 24.20232 | 1.200624 | 28 | 20.15812 | 0 | 0.0000000000e+00 | *** |
| Control - F55 | -15.6824 | 1.245946 | 28 | -12.5868 | 4.7E-12 | 4.7003512194e-12 | *** |
| F1 - F2 | 1.120385 | 1.153522 | 28 | 0.971273 | 0.865719 | 8.6571850977e-01 | |
| F1 - F54B | 30.16816 | 1.153522 | 28 | 26.15309 | 0 | 0.0000000000e+00 | *** |
| F1 - F55 | -9.71659 | 1.200624 | 28 | -8.09296 | 7.91E-08 | 7.9147363663e-08 | *** |
| F2 - F54B | 29.04777 | 1.153522 | 28 | 25.18181 | 0 | 0.0000000000e+00 | *** |
| F2 - F55 | -10.837 | 1.200624 | 28 | -9.02612 | 8.51E-09 | 8.5117921600e-09 | *** |
| F54B - F55 | -39.8848 | 1.200624 | 28 | -33.22 | 0 | 0.0000000000e+00 | *** |

| Sample | emmean | SE | df | lower.CL | upper.CL | .group |
| --- | --- | --- | --- | --- | --- | --- |
| F54B | 3.364866 | 0.815663 | 28 | 1.117927 | 5.611805 | a |
| Control | 27.56719 | 0.881017 | 28 | 25.14022 | 29.99416 | b |
| F2 | 32.41264 | 0.815663 | 28 | 30.1657 | 34.65958 | c |
| F1 | 33.53302 | 0.815663 | 28 | 31.28609 | 35.77996 | c |
| F55 | 43.24962 | 0.881017 | 28 | 40.82265 | 45.67659 | d |
